# Isolation of phenolic compounds from eco‐friendly white bee propolis: Antioxidant, wound‐healing, and anti‐Alzheimer effects

**DOI:** 10.1002/fsn3.3888

**Published:** 2023-12-07

**Authors:** Adem Necip, Ibrahim Demirtas, Seçil Erden Tayhan, Mesut Işık, Sema Bilgin, İsmail Furkan Turan, Yaşar İpek, Şükrü Beydemir

**Affiliations:** ^1^ Department of Pharmacy Services, Vocational School of Health Services Harran University Şanlıurfa Türkiye; ^2^ Department of Pharmaceutical Chemistry, Faculty of Pharmacy Ondokuz Mayıs University Samsun Türkiye; ^3^ Department of Pharmaceutical Biotechnology, Faculty of Pharmacy Tokat Gaziosmanpasa University Tokat Türkiye; ^4^ Department of Bioengineering, Faculty of Engineering Bilecik Seyh Edebali University Bilecik Türkiye; ^5^ Department of Medical Laboratory Techniques, Vocational School of Health Services Gaziosmanpasa University Tokat Türkiye; ^6^ Plant Research Laboratory‐B, Department of Chemistry, Faculty of Science Cankiri Karatekin University Cankiri Türkiye; ^7^ Department of Biochemistry, Faculty of Pharmacy Anadolu University Eskişehir Türkiye

**Keywords:** anti‐Alzheimer, antioxidant capacity, isolation of phenolics, phytochemical, propolis, wound‐healing

## Abstract

This study presents the first findings regarding extraction, isolation, enzyme inhibition, and antioxidant activity. The oral mucosal wound‐healing process was investigated using propolis water extract (PWE) incubation with gingival fibroblast cells and concluded that propolis was effective on the oral mucosal wound‐healing pattern compared to untreated controls. Additionally, phenolic compounds (fraxetin, apigenin, galangin, pinobanksin, chrysin, etc.) were isolated from propolis, and their chemical structures were elucidated using comprehensive spectroscopic methods. The antioxidant and anti‐Alzheimer potential activities of PWE and some isolated compounds were screened and revealing their inhibitory effects on acetylcholinesterase (AChE) with IC_50_ values ranging from 0.45 ± 0.01 to 1.15 ± 0.03 mM, as well as remarkable free‐radical scavenging and metal reduction capacities. The results suggest that these compounds and PWE can be used as therapeutic agents due to their antioxidant properties and inhibitory potential on AChE. It can also be used for therapeutic purposes since its wound‐healing effect is promising.

## Graphical abstract

Isolated compounds from the propolis and PWE can be utilized as therapeutic agents due to their antioxidant properties and inhibitory potential on AChE. In addition, since its wound‐healing effect is promising, it can be used for treatment purposes.

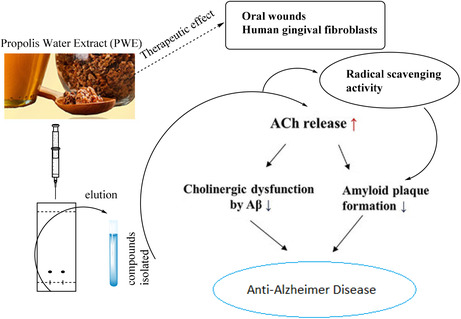

## 1 INTRODUCTION

Wound‐healing is the body's general response to repairing tissue damage and restoring tissue integrity following an injury. After an injury occurs, an inflammatory response is triggered, and fibroblast cells play a crucial role in producing collagen for the renewal of connective tissue. Wound‐healing is a dynamic and systematic process that can be divided into four distinct stages: inflammation, hemostasis, proliferation, and tissue remodeling (Gonzalez et al., 2016; Sharma et al., 2022). Gingival fibroblasts, as oral connective tissue cells, are responsible for collagen deposition during the wound‐healing process in dentistry (Eslami et al., 2017). Oral mucosal wounds are open lesions that occur as a result of various disorders occurring in the mouth. Infections may develop due to invasion of microorganisms or contaminants that can be life‐threatening. These wounds occur frequently, and healing of these lesions is very important (Gulinelli et al., 2008).

The wound‐healing process can be accelerated by applying natural products that have medicinal properties. Extensive research has been conducted on the wound‐healing properties of these natural products, characterized by their anti‐inflammatory, antioxidant, antibacterial, and pro‐collagen synthesis properties. These medicinal qualities are often attributed to bioactive phytochemical constituents originating from various chemical families, including alkaloids, essential oils, flavonoids, tannins, terpenoids, saponins, and phenolic compounds (Thakur et al., 2011; Zhao et al., 2023). Each of these bioactive agents may have different functions in the context of wound‐healing. For example, while saponins have the capacity to increase pro‐collagen synthesis (Chandel & Rastogi, 1980), tannins and flavonoids exhibit antiseptic and antibacterial activities (Harbone, 1973). These phytochemicals have the capacity to modulate one or more stages of the complex wound‐healing process. Furthermore, they are easily absorbed by the superficial layers of the skin. Given these remarkable properties, natural products and their associated phytochemicals assume pivotal roles in the field of wound‐healing and serve as valuable bases in the development of new synthetic compounds specifically for this purpose (Biswas & Mukherjee, 2003; Ibrahim et al., 2018).

Natural products have been used in folk medicine around the world for thousands of years. Among these products, propolis stands out and is used in the treatment of various systemic diseases (Aksit et al., 2014; Soleimani et al., 2021). Secondary metabolites reveal many biological activities (Boulechfar et al., 2022; Elmastas et al., 2018). A large number of drugs contain natural products as the main active ingredients due to their bioactive compounds, and the isolation and identification of the corresponding compounds have attracted great attention recently (Koldaş et al., 2015; Shen et al., 2023). Propolis contains high polyphenolic compounds and is used to treat various disorders. In many countries, propolis is used in traditional medicine, cosmetics, and food industries (Omer et al., 2023; Viuda‐Martos et al., 2008). It has also many pharmacological activities such as antioxidants (Ding et al., 2021), antimicrobial (Abdullah et al., 2020), antiamoebic (Zullkiflee et al., 2023), antidiabetic, antihypertensive (Farida et al., 2023), antiviral (Ożarowski & Karpiński, 2023), anti‐inflammatory (El‐Guendouz et al., 2017), anti‐allergic, hepatoprotective (Kolankaya et al., 2002), analgesic–anesthetic activity (Orsatti & Sforcin, 2012), immuno‐modulatory (Zullkiflee et al., 2022), and anticancer properties (Campoccia et al., 2021). Propolis is a functional food so many researchers focus on propolis attributing to its biological properties (Busch et al., 2017). The major active components of propolis are caffeic acid phenyl ester (CAPE) flavonoids such as chrysin, drupanin, cardanol, pinobanksin, galangin, and pinocembrin (Yen et al., 2017).

It has been reported in many studies that oxidation damages in many biomolecules such as proteins, lipids, and DNA cause many diseases such as diabetes, Alzheimer's, cancer, or atherosclerosis (Heinecke, 1997; Paz‐Elizur et al., 2008; Takım & Işık, 2020). As a result, it is known that antioxidants, which play an important role in preventing oxidation, reduce the risk of a wide variety of diseases. Recent studies have shown that compounds such as flavonoids, phenolics, or terpenes isolated from natural products show strong antioxidant activity (Abdullah et al., 2020; Necip & Mesut, 2019; Nieto et al., 1993; Rocha et al., 2023). Many antioxidants, which play a significant role in reducing oxidative stress in the treatment of Alzheimer's disease (AD), also have an inhibitory effect on acetylcholinesterase (AChE). It is known that these inhibitors simultaneously protect cells from oxidative damage and enhance antioxidant production (Chaudière & Ferrari‐Iliou, 1999; Kavaz Yüksel et al., 2021).

Propolis is a viscous, balsamic resin material that honeybees use to build and protect their hives (Al‐Khayri et al., 2022; Kavaz et al., 2022; Sommez et al., 2005). This natural material consists of resin (50%), wax and essential aromatic oils (30%), saliva of bees (10%), pollen (5%), and various substances such as amino acids, minerals, and bioflavonoids (5%). Some of these components may also play significant biomodulatory functions in connective tissue activity and collagen synthesis. Therefore, propolis has shown promising potential for various dental applications, including the prevention of dental caries, reduction of oral mucositis caused by chemotherapy, oral pathology, gum, and periodontal diseases (Gulinelli et al., 2008; Kłósek et al., 2021; LS, 2014; Yang et al., 2022).

Furthermore, it is important to note that propolis can have certain side effects, with the most common one being an allergic reaction to the resinous wax‐like material. People who are sensitive or allergic to bee products may experience adverse reactions when using propolis. Additionally, propolis preparations often contain high levels of alcohol, which can potentially lead to severe systemic disorders, especially when consumed in excessive amounts (Burdock, 1998; Ebadi & Fazeli, 2021). Since raw propolis is a resinous substance, it should be ground into powder and then extracted with ethanol or water. Since most bioactive compounds are lipophilic and soluble in ethanol, they are widely used in the extraction process (Ebadi & Fazeli, 2021; Zullkiflee et al., 2023). It was found that most of the active ingredients in propolis were extracted from the ethanol/water mixture at a concentration of 70%–80%. Liquid extracts prepared in this way have disadvantages such as alcohol intake, intolerance to alcohol in some people, and unsuitability for use in pediatrics due to high ethanol concentration (Park & Ikegaki, 1998). Therefore, it is medically important to use water instead of ethanol in the preparation of propolis.

In this context, the use of environmentally friendly natural propolis is very effective in the treatment of oral mucosal wounds due to its various therapeutic advantages such as low cost and biocompatibility with human cells. Therefore, the main components of commercially available white propolis are flavonoids, terpenoids, and aromatic carboxylic acids which are also used as food supplements by other companies. Additionally, this study elucidated the biological effects of aqueous propolis extract based on the widespread use of alcoholic extracts frequently found in the existing literature. This distinction represents a significant and noteworthy contribution.

Therefore, there is a growing interest in studying and identifying new compounds with bioactive properties derived from natural substances. These studies aim to discover and understand the potential benefits of these natural antioxidants in combating oxidative stress, reducing cellular damage, and improving overall health. Within the scope of this work, the wound‐healing process was investigated by the in vitro scratch assay, which is an easy, inexpensive, and well‐developed method for analysis of in vitro cell migration and proliferation using white PWE. The phytochemical study of Turkish PWE has led to the isolation of phenolic derivatives. Their chemical structures were elucidated using comprehensive spectroscopic methods. AChE inhibitory potential and antioxidant activity of the PWE and phenolic compounds isolated from it were screened.

## 2 MATERIALS AND METHODS

### 2.1 Chemicals and solvents

Commercial reagents were purchased from standard chemical suppliers (Merck and Sigma). Human gingival fibroblast cell line was kindly provided by Gaziosmanpasa University, Faculty of Dentistry. All reagents used for mouth sores and human gingival fibroblasts were of high purity and were purchased from Merck, Fluka, and/or Sigma‐Aldrich.

### 2.2 Isolation and identification of phenolic contents from Turkish white propolis

The mass spectrometry (MS) spectra were obtained with HPLC‐TOF/MS spectrum recorded in the negative ion mode on an Agilent 6210 ESI‐TOF mass spectrometer (Agilent Technologies). Silica gel (200–300 mesh, Merck) and Sephadex LH‐20 (Sigma) were used for column chromatographies. Pre‐coated silica gel GF254 (Merck) plates were used for thin‐layer chromatography. Spots were detected under UV light, and the plates were sprayed with anisaldehyde/sulfuric acid and then heated at 110°C. NMR spectra were recorded at 600 MHz (1H) and 150 MHz (13C) from Agilent Technologies; residual solvent peaks were used as internal references. Phenolic derivatives were isolated from the extracts for the phytochemical investigation of white Turkish propolis collected from Samsun (Yakakent), Turkey. The isolated compounds are as follows: fraxetin (1), ferulic acid (2), 3, 4‐dimethoxycinnamic acid (3), tectochrysin (4), pinostrobin (5), caffeic acid (6), pinobanksin (7), chrysin (8), pinocembrin (9), genkwanin (10), 3,7‐di‐O‐methylquercetin (11), 3‐methyl kaempferol (12), 3‐O‐methylquercetin (13), apigenin (14), galangin (15), rhamnetin (16), p‐coumaric acid (17), and rhamnocitrin (18). Their chemical structures were elucidated using comprehensive spectroscopic methods such as HREI‐MS and 1D and 2D NMR (see Figures S1 and Tables S1).

**FIGURE 1:**
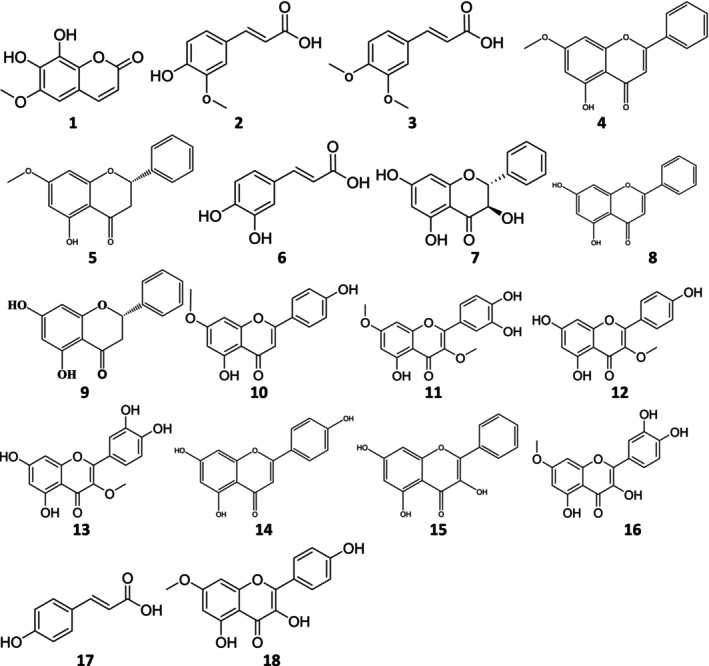
Molecular structures of isolated phenolic compounds from Turkish White Propolis Molecular structures of isolated phenolic compounds from Turkish White Propolis (**1**) Fraxetin; (**2**) Ferulic acid; (**3**) 3,4‐Dimethoxycinnamic acid; (**4**) Tectochrysin; (**5**) Pinostrobin; (**6**) Caffeic acid; (**7**) Pinobanksin; (**8**) Chrysin; (**9**) Pinocembrin; (**10**) Genkwanin; (**11**) 3,7‐Di‐*O*‐methylquercetin; (**12**) 3‐Methyl kaempferol; (**13**) 3‐*O*‐methylquercetin; (**14**) Apigenin; (**15**) Galangin; (**16**) Rhamnetin; (**17**) *p*‐Coumaric acid; (**18**) Rhamnocitrin.

8.9 g of white Turkish propolis collected from Samsun was extracted with water and fractionated with a previously developed isolation process (Ipek et al., 2017). PWE was subjected to a 3:1 methanol‐chloroform solvent system in Sephadex LH‐20 column packing material to obtain seven main fractions. From these fractions, the fraction A (0.73 g), fraction B (3.95 g), fraction C (1.76 g), fraction D (1.79 g), fraction E (0.34 g), fraction F (0.17 g), and fraction G (0.13 g)‐fused fractions (C–E) were again fractionated to obtain 18 phenolic compounds (Figure 1).

Figure S1 shows the 1H, 13C, and HSQC NMR spectra of fraction A as a mixture of volatile contents such as fatty acids. Proton NMR spectra of the fraction and subfractions of fraction B which exhibit five major peaks: 4.12, 2.32, 1.65, 1.28, and 0.91 ppm (Figure S2). The triplets at 4.12 and 2.32 are diagnostic for methylene groups attached to the O‐ and carbonyl moieties of an ester group, respectively. The very strong peak at 1.28 indicates the presence of the hydrocarbon chain. Finally, the peak at 1.65 ppm represents the linkage or CH_2_ groups that connect the hydrocarbon chains to the ester‐bound methylene. Peak integrations are consistent with a total chain length of 45–50 methylene groups. Also, note the strong similarity of the sample spectrum with the reference spectrum of butyl palmitate shown in Figure S2. Although there are some rare weak bands indicating low levels of other molecules, the strong signal and sharp peaks in Figure S3 indicate that the sample consists primarily of one or more chemically similar, long‐chain esters such as palmitate. This is again indicative of a natural wax. For example, beeswax consists largely of myricylpalmitate, CH_3_(CH_2_)_29_OC=O(CH_2_)_14_CH_3_; this is consistent with chain length estimates obtained from both peak shifts and peak integration data. The 2D COSY‐NMR spectrum shown in Figure S4 provides further confirmation of this assignment, as it clearly shows that the methylene groups at 1.65 ppm bind to both the ester peaks at 4.06 and 2.296 ppm and the main peak at 1.28 ppm. The recent peaks are not linked.

From these obtained fractions, fraction C was separated by Sephadex LH‐20 column chromatography in a 5:5:1 diethyl ether–methanol–hexane solvent system to obtain the subfraction. Subfractions of fraction C were subjected again to Sephadex LH‐20 column chromatography in a 5:5:1 ethyl acetate–methanol–hexane solvent system to give pure compounds. As a result of the chromatographic treatment of the fraction C, pure compounds were isolated and purified as compound 1 (fraxetin, Figure S5) from the subfraction C‐5/5, compound 2 (ferulic acid, Figure S6) from subfraction C‐4/3, and compound 3 (3,4‐dimethoxy cinnamic acid, Figure S7) from subfraction C‐3/6. Compound 4 (tectochrysin, Figure S8) and compound 5 (pinostrobin, Figure S9) were also separated from fraction C‐3/4 used in silica gel column chromatography in hexane–ethyl acetate solvents. The other D fraction was fractionated by Sephadex LH‐20 column chromatography in a 5:5:1 diethyl ether–methanol–hexane solvent system. Subfractions of fraction D were subjected to a Sephadex LH‐20 column in a 5:5:1 ethyl acetate–methanol–hexane solvent system to obtain pure compounds. Pure compounds were isolated as compound 6 from fraction D‐4/4 (caffeic acid, Figure S10), compound 7 from fraction D‐3/5 (pinobanksin, Figure S11), and compound 8 from fraction D‐3/3 (chrysin, Figure S12). Additionally, subfraction D‐3/2 was fractionated to yield compound 9 (pinocembrin, Figure S13), compound 10 (genkwanin, Figure S14), compound 11 (3,7‐di‐O‐methylquercetin, Figure S15), compound 12 (3‐O‐methyl kaempferol, Figure S16), and compound 13 (3‐O‐methylquercetin, Figure S17), with silica column chromatography increasing the polarity from hexane to ethyl acetate. Similarly, fraction E was fractionated by Sephadex LH‐20 column chromatography in a 5:5:1 diethyl ether–methanol–hexane solvent system to obtain the subfraction. This subfraction was further subfractionated to obtain pure compounds in a 5:5:1 ethyl acetate–methanol–hexane solvent system with a Sephadex LH‐20 column. Compound 14 from fraction E‐5/4 (apigenin, Figure S18), compound 15 from fraction E‐3/1 (galangin, Figure S19), compound 16 from fraction E‐6/4 (rhamnetin, Figure S20), compound 17 from fraction E‐5/5 (*p*‐coumaric acid, Figure S21), and compound 18 from fraction E‐4/2 (rhamnocitrin, Figure S22) were isolated and purified. The structures of all isolated compounds were determined using NMR spectroscopy and confirmed with literature data (Tables S1).

### 2.3 In vitro wound‐healing assay

Two‐dimensional in vitro cell migration is used to investigate the re‐colonization ability of cell populations (Johnston et al., 2016). During this experiment, cells are placed on a culture dish and growth cell monolayer. An artificial wound is then created with a p200 pipette tip, and images of collective cell spreading resulting from combined cell migration and proliferation are captured for 24 and 48 h (Khurshid et al., 2017). In this study, the wound‐healing effect of PWE was preliminary investigation by in vitro scratch assay. This assay is most commonly used when measuring migration rate because it provides a simple and economical setup in the hands of experienced users. The first step was to create an artificial wound in the cell monolayer. Image capture was then performed at baseline and at regular intervals during cell migration. Finally, the migration rate of the cells was quantified by comparing the cell micrographs (Alizadeh et al., 2011). In this study, an oral mucosal wound‐healing model was established using human gingival fibroblasts (hGFs) in vitro.

In this context, first, MTT (3‐(4,5‐dimethylthiazol‐2‐yl)‐2,5‐diphenyltetrazolium bromide) assay was performed with hGFs to determine effective dose for propolis. hGFs were suspended in fresh culture medium (DMEM supplemented with 10% FBS), and the cell suspension was transferred into 96 well plates at an initial density of 5 × 10^3^ cells/well. This test was performed at 24 concentrations (from 500 μg/mL to 0.058 ng/mL), and the cells were left in contact with propolis for 48 h. A stock solution of propolis was prepared in DMSO (<0.1% in culture medium) and filter sterilized before adding it to the culture plate. After incubation, 100 μL of MTT solvent (5 mg/mL) was added to each well and then incubated at 37°C for 3 h. For viability analysis, MTT was removed, the formazan product was dissolved in 100 μL of DMSO, and absorbance was measured at 570 nm with a multimode microplate reader.

According to the results obtained from this analysis, for in vitro scratch assay, the cell monolayer was scraped with sterile p200 pipette tip to create a scratch in a straight line. By washing the cells with culture medium, debris was removed and the scratch edge was smoothed (Moraes et al., 2011). Cells were then incubated with media containing teucrioside at a 50 μg/mL as determined by MTT assay. After 0, 24, and 48 h incubation, cell images were captured with a phase contrast inverted microscope and analyzed quantitatively. Finally, the wound‐healing percentage was calculated, and the results were plotted.

### 2.4 FRAP and CUPRAC methods as metal reduction

The ferric cyanide (Fe^3+^) reducing antioxidant power (FRAP) assay was performed using the modified method of the previously described Oyaizu method (Oyaizu, 1986). The complex is formed when the method is reduced to the ferrous (Fe^2+^) ion to ferric tripyridyl triazene (Fe^3+^‐TPTZ) complex at 700 nm. It is based on the spectrophotometric measurement of the complex. The reduction capacity for cupric ions (Cu^2+^) was determined by cupric ions reducing assay (CUPRAC) as previously described. A volume of 0.25 mL neocuproine (7.5 mM) in ethanol, 0.25 mL NH_4_Ac (1 M), and 0.25 mL CuCl_2_ (0.01 M) were mixed with isolated compounds, PWE, and different amounts of standards (10, 20, and 40 μg/mL).

### 2.5 DPPH and ABTS radical scavenging activities

The DPPH scavenging activities of PWE and isolated compounds were determined according to the method described by Blois (1958). In the method, the stable DPPH radical is removed by the free‐radical scavenging activity of the sample. Briefly, ethanol extract (10, 20, and 40 μg/mL) was prepared from the sample, and then the volume was adjusted to 3 mL with ethanol. Then, the prepared DPPH solution (1 mL, 0.1 M) was added, followed by incubation for 30 min in the dark. After incubation, the DPPH elimination activity of the sample was measured spectrophotometrically (Necip et al., 2021).

ABTS also forms a relatively stable free‐radical that removes color in its non‐radical form (Shirwaikar et al., 2006). Spectrophotometric analysis of ABTS˙^+^ scavenging activity was determined according to the Re method. In this method, the sample is added to the previously formed ABTS radical solution, and the amount of ABTS˙^+^ remaining after a fixed time is spectrophotometrically at 734 nm. Then, 1 mL of ABTS˙^+^ solution at different concentrations (10, 20, and 40 μg/mL) was added to the sample. The absorbance was recorded 30 min after mixing, and the percentage of radical scavenging was calculated for each concentration compared to the blank sample without scavenger (Necip & Durgun, 2022; Re et al., 1999).

### 2.6 Measurement of AChE activity

The inhibitory effects of propolis and the isolated compounds on AChE were tested by Ellman's spectrophotometric method (Ellman et al., 1961). Briefly, 50 μL of 5,5′‐dithio‐bis(2‐nitro‐benzoic) acid compound (DTNB) and 100 μL of Tris–HCl solution (1 M, pH 8.0) and 50 μL AChE from electrophorus electricus (5.32 × 10^−3^ U) solution was incubated and stirred at 30°C for 15 min. Finally, the reaction was started by adding 50 μL of acetylthiocholine iodide (AChI), which was used as a substrate. Enzymatic hydrolysis of the substrate was recorded spectrophotometrically at a wavelength of 412 nm (Işık et al., 2020; Wright & Plummer, 1973). In the study, the inhibitory effect of propolis and the isolated compounds on AChE at various concentrations was investigated. Percent inhibition values and IC_50_ values were determined using a graph (Kilic et al., 2020).

### 2.7 Statistical study

Analysis of the data was performed using GraphPad Prism version 6 (GraphPad Software). Results are shown as mean ± standard deviation (95% confidence intervals). Differences between datasets were considered statistically significant when the *p*‐value was less than .05.

## 3 RESULTS

White propolis is a novel purified propolis extract that contains the effective components of propolis (Figure 1) in a form that can be easily absorbed and used in vivo, as well as having relatively high quality and satisfactory processability, color, flavor, taste, and properties. It has mucosal wound‐healing activity and is completely free of dark‐colored impurities.

**FIGURE 2:**
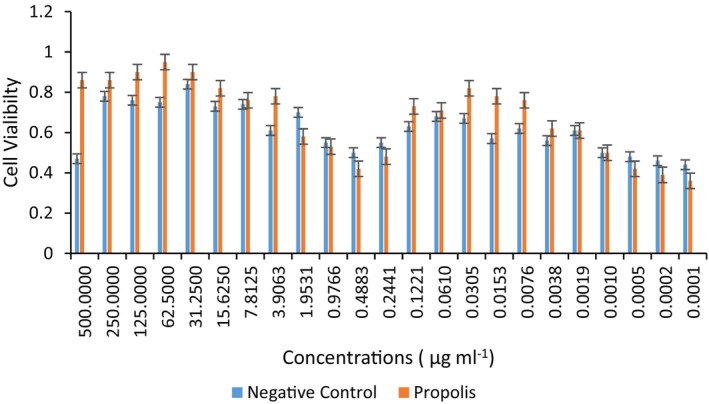
Effects of water extract of propolis on the proliferation of hGF cell line. The red‐boxed concentration (62.5 μg/mL) is determined as an effective dose for scratch assay.

This new type of extraction method also showed remarkable activity against mucosal wound‐healing and free from the highly negative value of organic solvent residues. As shown in Figure 2, it is both safe and easy to recognize single water‐based propolis from its colors. For this reason, white propolis is the extraction of propolis containing high amounts of heat‐resistant phenols and flavones with only ultrapure water and attracts attention. It is preferred in food supplements due to its biocompatibility, green approach, and eco‐friendly nature.

To determine the wound‐healing effect of propolis in vitro, hGF cells were incubated with this natural product. First, an MTT assay was performed, and cell viabilities were calculated to determine the effective dose for the scratch assay. As a result, hGF cells incubated with propolis solution at a concentration of 62.5 μg/mL for 48 h showed the highest cell viability versus the negative control (Figure 2).

According to the MTT assay results, in vitro scratch assay was carried out with hGFs (Figure 4a) and propolis solution at a concentration of 62.5 μg/mL.

**FIGURE 3:**
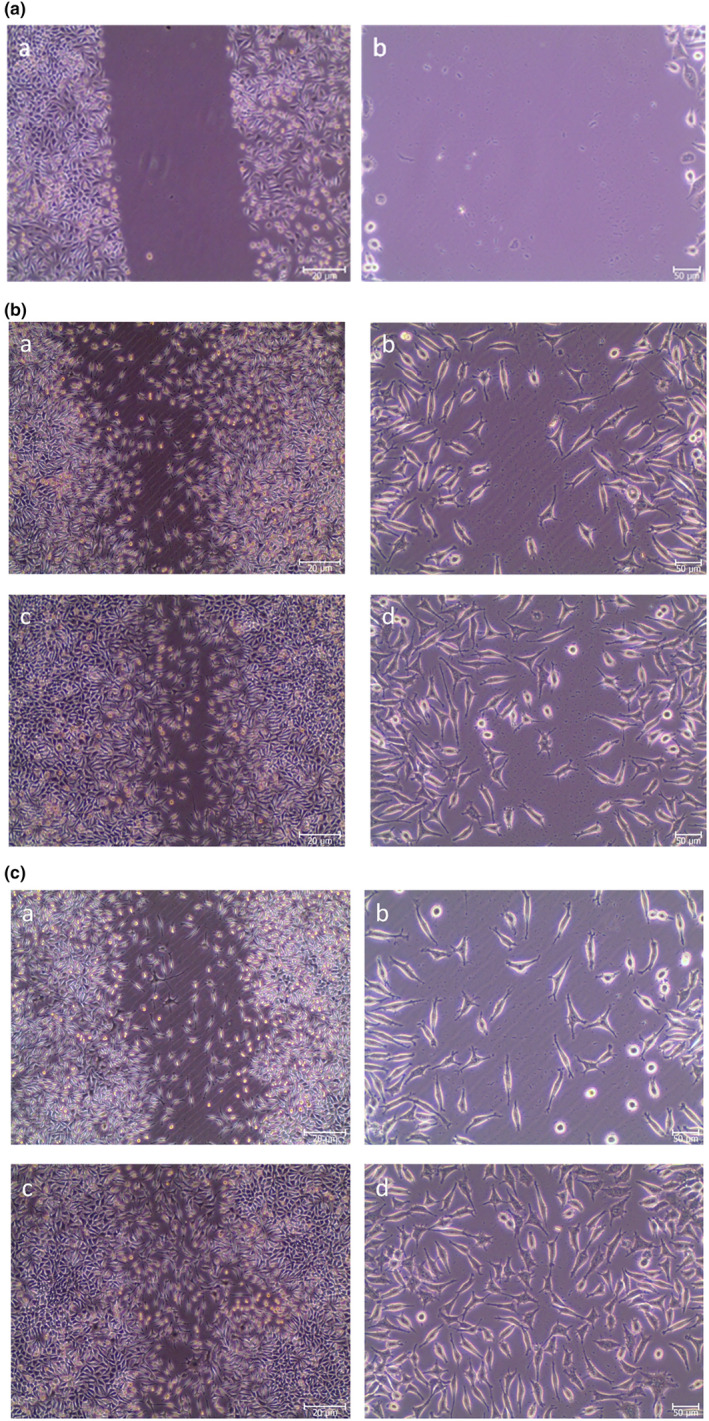
(a) Inverted light microscope images of hGFs after scratching (0 h). Images (a and b) are representing 4× and 10× magnifications, respectively. The scale bars indicated 20 μm (a) and 50 μm (b); (b) Inverted light microscope images of hGFs after scratching which were cultured in negative control wells. Images (a, c and b, d) are representing 4× and 10× magnifications, respectively. Figure a and b are pointing out 24 h and also c and d are showing 48 h after scratching. The scale bars indicated 20 μm (a, c) and 50 μm (b, d); (c) Inverted light microscope images of hGFs after scratching which were cultured in propolis incubation wells. Images (a, c and b, d) are representing 4× and 10× magnifications, respectively. Figure a and b are pointing out 24 h and also c and d are showing 48 h after scratching. The scale bars indicated 20 μm (a, c) and 50 μm (b, d).

**FIGURE 4:**
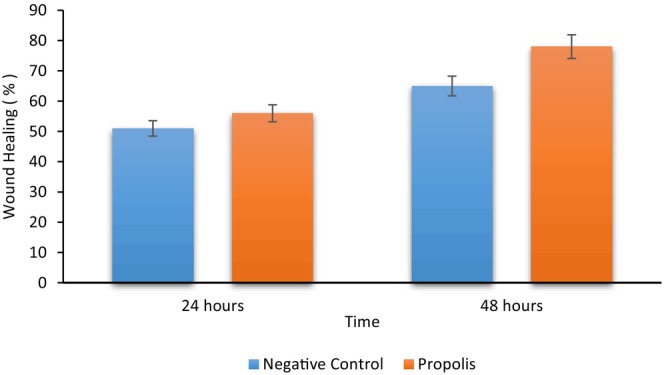
Wound‐healing effects of propolis against negative control at 24 and 48 h.

It was clearly seen that after 24 and 48 h of incubation of propolis with hGF cells, the wound‐healing percentage was calculated as 55.9% and 79.3%, respectively (Figure 3b,c). When these data were compared to untreated controls at 24 and 48 h (51% and 65%, respectively), it was concluded that the water extract of propolis was effective in an in vitro oral mucosal wound‐healing model (Figure 4).

**FIGURE 5:**
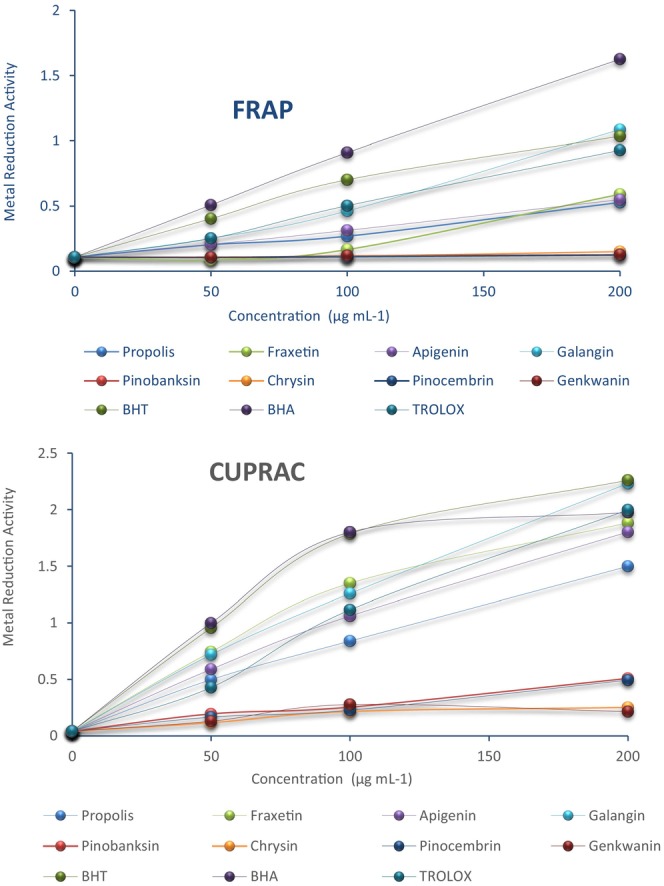
Metal‐reducing capacity (FRAP and CUPRAC assays) of propolis and compounds isolated from propolis at different concentrations (50, 100, and 150).

DPPH, ABTS, radical scavenging assays, FRAP, and CUPRAC methods showing metal reduction capacity were used to evaluate the antioxidant activities of isolated compounds. Effects of fraxetin **(1)**, pinobanksin **(7),** chrysin **(8)**, pinocembrin **(9)**, genkwanin **(10)**, apigenin **(14)**, and galangin **(15)** on DPPH and ABTS radical scavenging activities at 0.2 mg/mL are presented in Table 1. The compounds (**1**, **14**, and **15)** exhibited strong free‐radical scavenging activity close to standard antioxidants such as BHT, BHA, and trolox, over 60%, while the compounds (**8–10)** showed low scavenging activity. Moreover, compound **1** had the highest radical scavenging activities, which were stronger than standard antioxidants (BHT and BHA). The results of the metal reduction capacity at 0.2 mg/mL are examined. According to FRAP assays, compound **15** exhibited high metal reduction activity, while propolis and compounds **1** and **14** had moderate metal reduction capacity (Figure 5). According to CUPRAC analysis, compound **15** also has high metal reduction activity. Propolis and its compounds (**1**, **14**, and **15**) show a metal reduction capacity close to the standards (Figure 5). This effect is low in other compounds.

**TABLE 1:** The radical scavenging and AChE inhibitory potential of propolis water extract and compounds isolated from propolis.

Compounds	DPPH (0.2 mg/mL)^a^	ABTS (0.2 mg/mL)^a^	AChE (IC_50_)
Propolis	65.62 ± 1.2	64.59 ± 2.7	0.14 ± 0.01 mg/mL
Fraxetin	81.11 ± 1.7	87.44 ± 3.8	0.73 ± 0.04 mM
Apigenin	79.02 ± 2.2	59.41 ± 1.5	0.71 ± 0.03 mM
Galangin	44.73 ± 1.8	62.01 ± 1.9	0.79 ± 0.02 mM
Pinobanksin	3.55 ± 0.6	15.63 ± 0.9	0.45 ± 0.01 mM
Chrysin	Inactive	4.35 ± 0.7	0.64 ± 0.02 mM
Pinocembrin	Inactive	26.32 ± 1.3	1.10 ± 0.04 mM
Genkwanin	Inactive	8.95 ± 0.8	1.15 ± 0.03 mM
BHT^b^	51.57 ± 1.5	58.06 ± 2.4	NT
BHA^b^	80.20 ± 2.9	93.62 ± 3.6	NT
Trolox^b^	90.37 ± 3.8	89.99 ± 3.2	NT

*Note*: Data are mean ± standard deviation (*n* = 3).

Abbreviation: NT, not tested.

^a^ The percent (%) of ABTS and DPPH radical scavenging activity.

^b^ Standard antioxidants.

The inhibitory effect of propolis and its compounds (**1**, **7**, **8**, **9, 10**, **14**, and **15**) on AChE was determined by the activities in Table 1. We determined that IC_50_ values for propolis were 0.143 ± 0.01 mg/mL, 0.73 ± 0.04, 0.71 ± 0.03, 0.79 ± 0.02, 0.45 ± 0.01, 0.64 ± 0.02, 1.1 ± 0.04, and 1.15 ± 0.03 mM for isolated compounds (Table 1).

## 4 DISCUSSION

The main focus of the past research evaluating the effect of propolis extract on wound‐healing has been observed to increase healing performance with the ethanolic extract of propolis (Ebadi & Fazeli, 2021; Ocakci et al., 2006). In addition, the effect of different alcoholic propolis solutions on fibroblast cultures has been evaluated in many studies, and the results have shown that low propolis concentration is more effective and nontoxic for fibroblasts (Oda et al., 2016). Additionally, studies using an in vitro oral wound‐healing model have shown that propolis has an oral mucosal wound‐healing effect (Eslami et al., 2017; Furukawa et al., 2021; Gulinelli et al., 2008; Jongjitaree et al., 2022; Vo et al., 2021). On the other hand, some study groups have shown that it has no significant effect on fibroblast expansion (Al‐Shaher et al., 2004). In this study, incubating hGF cells with propolis solution at a concentration of 62.5 μg/mL for 48 h demonstrated the highest cell viability compared to the negative control. In addition, it was determined that the wound‐healing rate was high after 24 and 48 h of incubation with propolis. These data indicate the effectiveness of PWE on the in vitro oral mucosal wound‐healing model compared to untreated controls at 24 and 48 h. These results are consistent with findings reported in the literature regarding alcohol extracts.

Free‐radicals formed as a result of increased oxidative stress can damage cell macromolecules. In particular, modification of genetic material may represent the first step in carcinogenesis, mutagenesis, and aging. DNA damage products caused by oxidative stress‐induced double‐ or single‐stranded purine, pyrimidine, deoxyribose modifications, or DNA breaks and DNA cross‐links. Many studies have found free‐radical‐induced DNA damage in various cancer tissues (Paz‐Elizur et al., 2008; Sainz et al., 2012). Therefore, it has been hypothesized that radical scavenging antioxidants may prevent or limit the damage caused by free‐radicals (Valko et al., 2007). At the same time, antioxidants used to remove reactive oxygen species (ROS) formed during food processing (mechanical pressures, heating, and irradiation) also play an important role in protecting foods during storage and extending their shelf life.

It is well known that propolis has a strong antioxidant activity, containing substances such as polyphenols, terpenoids, steroids, carbohydrates, amino acids, vitamins, and minerals (Touzani et al., 2018). Pinocembrin, pinobanksin, chrysin, and galanging were detected in the propolis sample taken from the Populus spp. (poplar) plant species distributed in Europe, Asia, and North America (Bankova & Kuleva, 1989; Bankova et al., 1992; Jiaping et al., 1996; Garcia‐Viguera et al., 1993; Nagy et al., 1986), which have been reported as the main components. In another study, it was reported that the main components of the propolis sample taken from the Betula verrucosa (birch) plant species distributed in Northern Russia were apigenin and rhamnocitrin (Bankova et al., 2002; Popravko, 1978). Pinocembrin, pinobanksin, chrysin, galanging, apigenin, and rhamnocitrin were isolated as the main components of the propolis we used in the study. A study on 16 propolis from different regions showed that propolis from China, Hungary, Australia, and New Zealand had a strong DPPH free‐radical scavenging activity of over 60% (Kumazawa et al., 2004). In another study, the free‐radical scavenging percentage of a propolis from Mexico was found to be 64.8% (Vargas‐Sánchez et al., 2014). In another study, ABTS and DPPH assays showed free‐radical scavenging activity of 94.34% and 85%, respectively (Asgharpour et al., 2018).

In this study, an isolation study was carried out due to the high total phenolic content of propolis, which is considered an indicator of antioxidant capacity, as stated in many literatures. The antioxidant activity of propolis and some isolated compounds was evaluated using DPPH, ABTS˙^+^, and metal‐reducing power methods with BHA, BHT, and trolox as controls. In the DPPH and ABTS methods, standard antioxidants showed a strong DPPH and ABTS radical scavenging activity (range 51.6%–90.4%) at a concentration of 200 μg/ mL. The radical scavenging activity of propolis and Fraxetin **(1)**, Apigenin **(14)**, and Galangin **(15)** exhibited 59.4%–87.4% for ABTS and 44.7%–81.1% for DPPH at 200 μg/mL, respectively. The compounds (**1, 14**, and **15)** have greater radical scavenging activity than some standard compounds. The compounds (**1, 14**, and **15**) also have high metal‐reducing capacity. In a study on the scavenging activity of isolated compounds, the free‐radical scavenging activity of triterpenoids and flavonoids isolated from propolis was determined by xanthine–xanthine oxidase analyses and DPPH. While flavonoids showed high levels of DPPH scavenging activity in the range of 48%–83%, isolated triterpenoids and flavonoids showed significant superoxide anion activity (O_2_˙^−^, 50%–75%) (El‐Hady et al., 2013). The compounds (**1, 14**, and **15**) isolated in our study showed higher radical scavenging activity than the compounds of the above‐mentioned study.

It is known that AChE inhibitors used in the symptomatic treatment of the disease protect cells from oxidative damage and increase antioxidant production (Chaudière & Ferrari‐Iliou, 1999; Kavaz Yüksel et al., 2021). AChE, found in cholinergic neurons and the brain, hydrolyzes the neurotransmitter acetylcholine (ACh), causing neurodegenerative diseases. The change in AChE activity has an important role in determining the degree of neurotoxicity. Increased activity plays a role in the formation of *β*‐amyloid (Aβ), which accumulates in extracellular toxic plaques in Alzheimer's brains. Therefore, AChE inhibitors are important in the treatment of AD by slowing or stabilizing ACh hydrolysis. There are many synthetic drugs containing AChE inhibitors, such as rivastigmine, tacrine, and donepezil, which are widely used in the treatment of AD. According to literature results, it is known that commonly used AChE inhibitors have many side effects such as gastrointestinal discomfort and hepatotoxicity (Akocak et al., 2021; Işık & Beydemir, 2021; Oh et al., 2004; Yapar et al., 2021). In this study, we investigated the inhibitory activity of propolis and compounds isolated from propolis, a natural resource, on AChE. While propolis shows inhibitory activity against AChE at a concentration of 0.143 ± 0.01 mg/mL, fraxetin, apigenin, galangin, pinobanksin, chrysin, pinocembrin, and genkwanin inhibit AChE at IC_50_ range of 0.45 ± 0.01 mM to 1.15 ± 0.03 mM (Table 1). Pinobanksin exhibited the highest activity with IC_50_ = 0.45 ± 0.01 mM. In this context, a previous study has shown that treatment with antioxidant vitamins E and C significantly reduced AChE activity in the hippocampus of adult rats (Monteiro et al., 2007). In another study, it was reported that cycloartane–triterpenoid compounds with antioxidant effects significantly inhibit AChE (Areche et al., 2009). Additionally, 3β‐cycloartenol showed moderate inhibition on AChE with an IC_50_ value of 3.6 ± 0.1 μM and 59% free‐radical scavenging activity (El‐Hady et al., 2013).

As a result, compounds isolated from propolis have the potential to neutralize free‐radicals, convert cupric to cuprous and ferric to ferrous forms, and therefore show higher reducing activity. It is thought that these compounds can be used in the treatment of Alzheimer's patients because they are naturally induced AChE inhibitors.

## 5 CONCLUSION

In conclusion, our study findings suggest that application of white propolis at appropriate concentrations may be effective in promoting oral mucosal wound‐healing in vivo. These results are consistent with existing research in this field and, in particular, this study represents the first investigation of the oral mucosal wound‐healing properties of water extracts of propolis. It is also important to underline that there is an urgent need for further research in the fields of dentistry, medicine, and food preservation, taking advantage of the biological properties of this natural bee product. Both propolis and its isolated compounds exhibit remarkable abilities to scavenge radicals and reduce metals. The results of this study highlight their potential use as therapeutic agents due to their antioxidative properties and inhibitory effects on AChE. In fact, propolis and its isolated compounds have the potential to serve as natural antioxidants that alleviate oxidative stress, which is a key factor in the development of various diseases. Further research and applications in these areas are promising in exploiting the therapeutic potential of propolis and its components.

## AUTHOR CONTRIBUTIONS

**Adem Necip:** Investigation (equal); methodology (equal). **Ibrahim Demirtas:** Funding acquisition (equal); methodology (equal); supervision (equal); writing – original draft (equal). **Seçil Erden Tayhan:** Investigation (equal); methodology (equal). **Mesut Işık:** Investigation (equal); methodology (equal); writing – original draft (equal); writing – review and editing (equal). **Sema Bilgin:** Investigation (equal); methodology (equal). **İsmail Furkan Turan:** Methodology (equal). **Yaşar İpek:** Methodology (equal). **Şükrü Beydemir:** Supervision (equal); writing – review and editing (equal).

## FUNDING INFORMATION

This study did not receive any grant from funding agencies in the public, commercial, etc.

## CONFLICT OF INTEREST STATEMENT

The authors declare no conflict of interest.

## Supporting information


Appendix S1.

## Data Availability

The data that support the findings of this study are available from the corresponding author upon reasonable request.
