# Supplementary material for: Isolation of phenolic compounds from eco‐friendly white bee propolis: Antioxidant, wound‐healing, and anti‐Alzheimer effects
Source: Food Sci Nutr. 2023 Dec 7;12(3):1928–39. doi: 10.1002/fsn3.3888 (PMC10916560; doi:10.1002/fsn3.3888)
Supplement: Supplementary file 1 — Appendix S1. [file FSN3-12-1928-s001.docx]

**Isolation of phenolic compounds from eco-friendly white bee propolis: Antioxidant, wound healing and anti-Alzheimer effects**

Adem Necip^a^, Ibrahim Demirtas^b^, Seçil Erden Tayhan^c^, Mesut Işık^d,*^ Sema Bilgin^e^, İsmail Furkan Turan^c^, Yaşar İpek^f^, Şükrü Beydemir^g^

^a^*Department of Pharmacy Services, Vocational School of Health Services, Harran University, Şanlıurfa, 63300, Turkey*

*^b^Ondokuz Mayis University,Faculty of Pharmacy, Department of Pharmaceutical Vocational Sciences,Department of Pharmaceutical Chemistry, 55030, Samsun, Turkey*

^c^ *Department of Pharmaceutical Biotechnology, Faculty of Pharmacy, Tokat Gaziosmanpasa University, 60250, Tokat, Turkey*

^d^ *Department of Bioengineering, Faculty of Engineering, Bilecik Seyh Edebali University, Bilecik, 11230, Turkey*

^e^ *Department of Medical Laboratory Techniques, Vocational School of Health Services,*

*Gaziosmanpasa University, 60250, Tokat, Turkey*

*^f^ Plant Research Laboratory-B, Department of Chemistry, Faculty of Science, Cankiri Karatekin University, Cankiri, 18200, Turkey*

*^g^Department of Biochemistry, Faculty of Pharmacy, Anadolu University, 26470, Eskişehir, Turkey*

^⁎^Corresponding author:

Phone: +90 228 214 2119; *E-mail address:* [mesut.isik@bilecik.edu.tr](mailto:mesut.isik@bilecik.edu.tr); mesutisik16@gmail.com

**
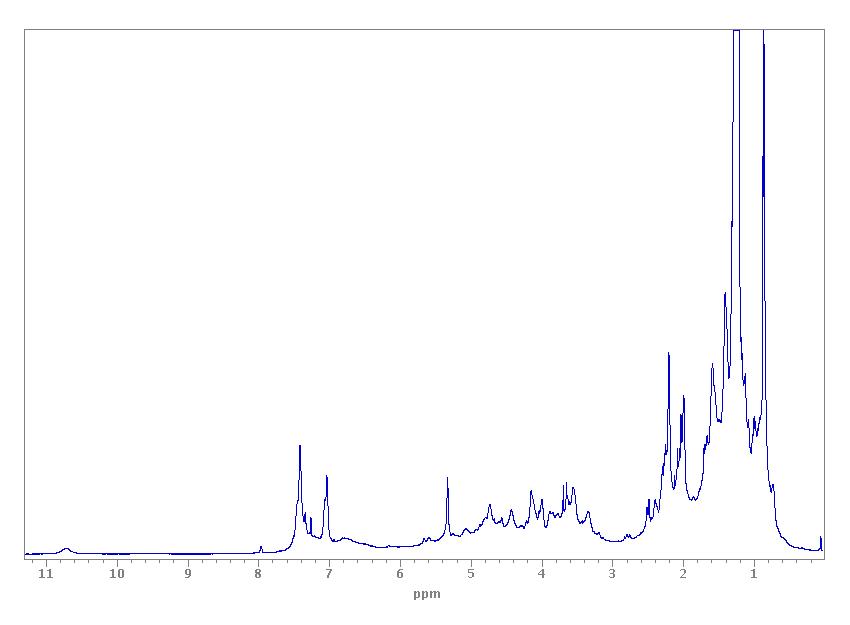
(a)**

**
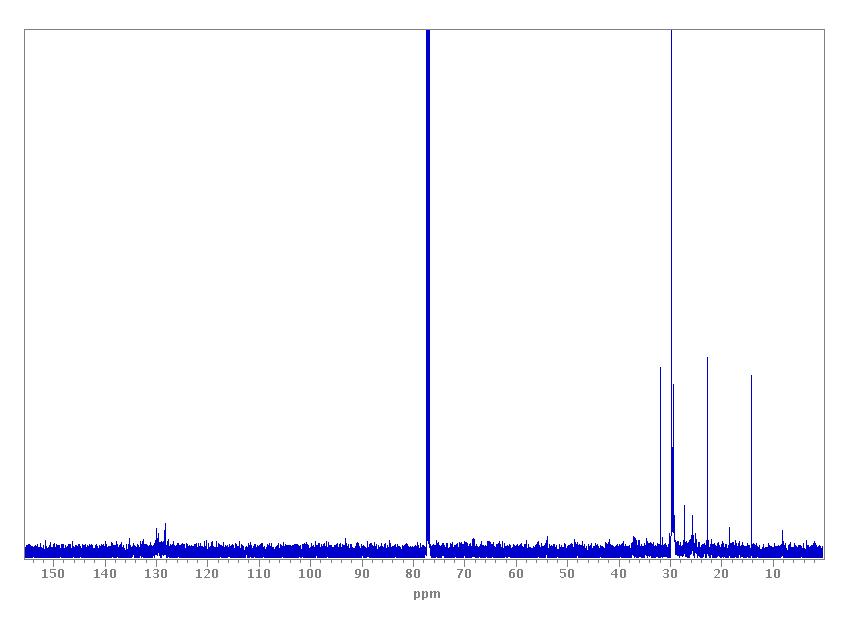
(b)**

**
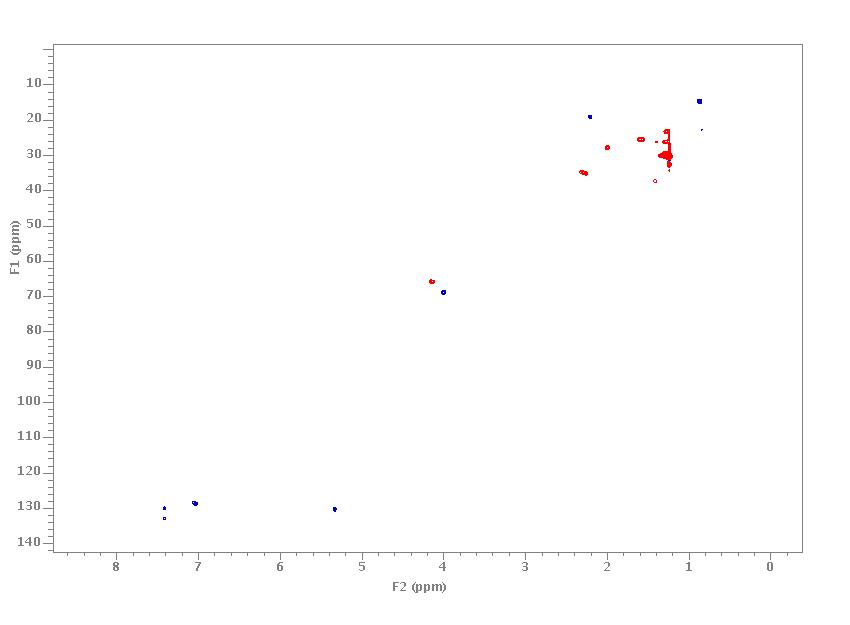
(c)**

**Figure S1.** Proton (a), carbon (b) and HSQC NMR spectra of fraction A as mixture of fatty acids and volatile contents.

**
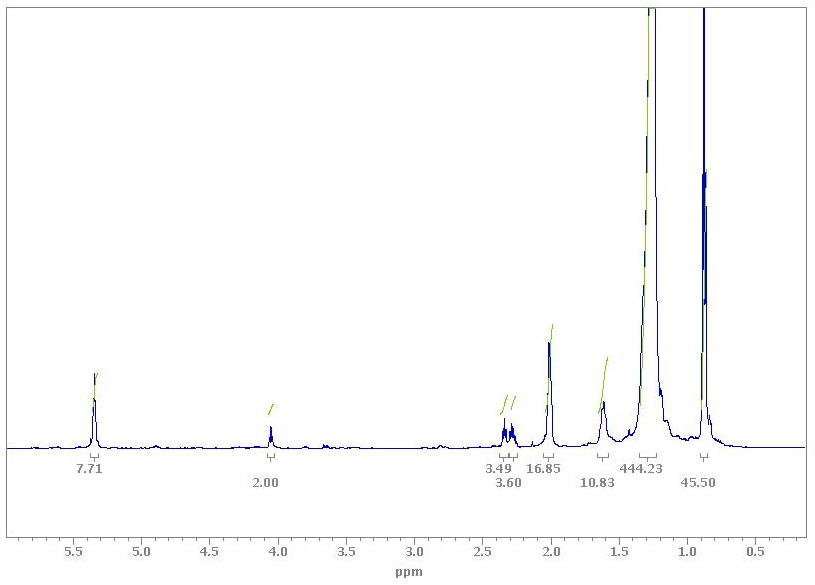
(a)**

**
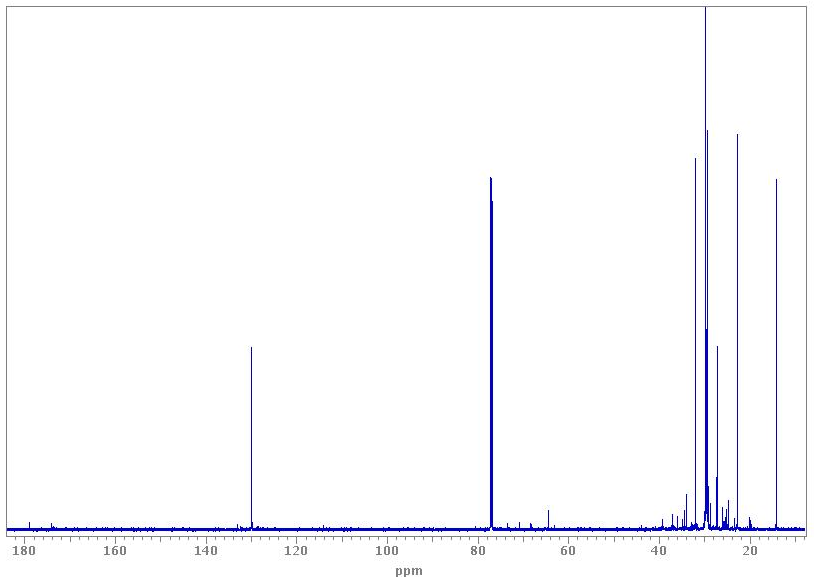
(b)**

**
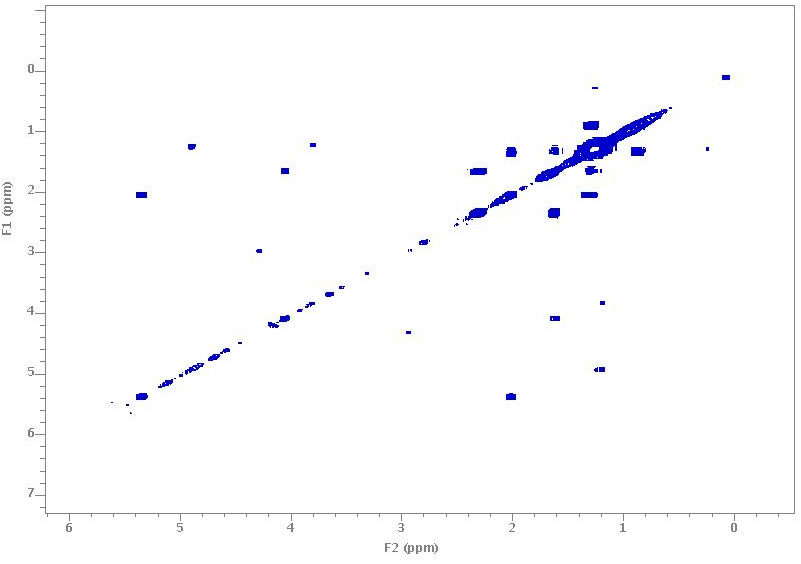
(c)**

**Figure S 2.** NMR spectra of proton (a), carbon and COSY (c) obtained from second sub-fraction of fraction B.


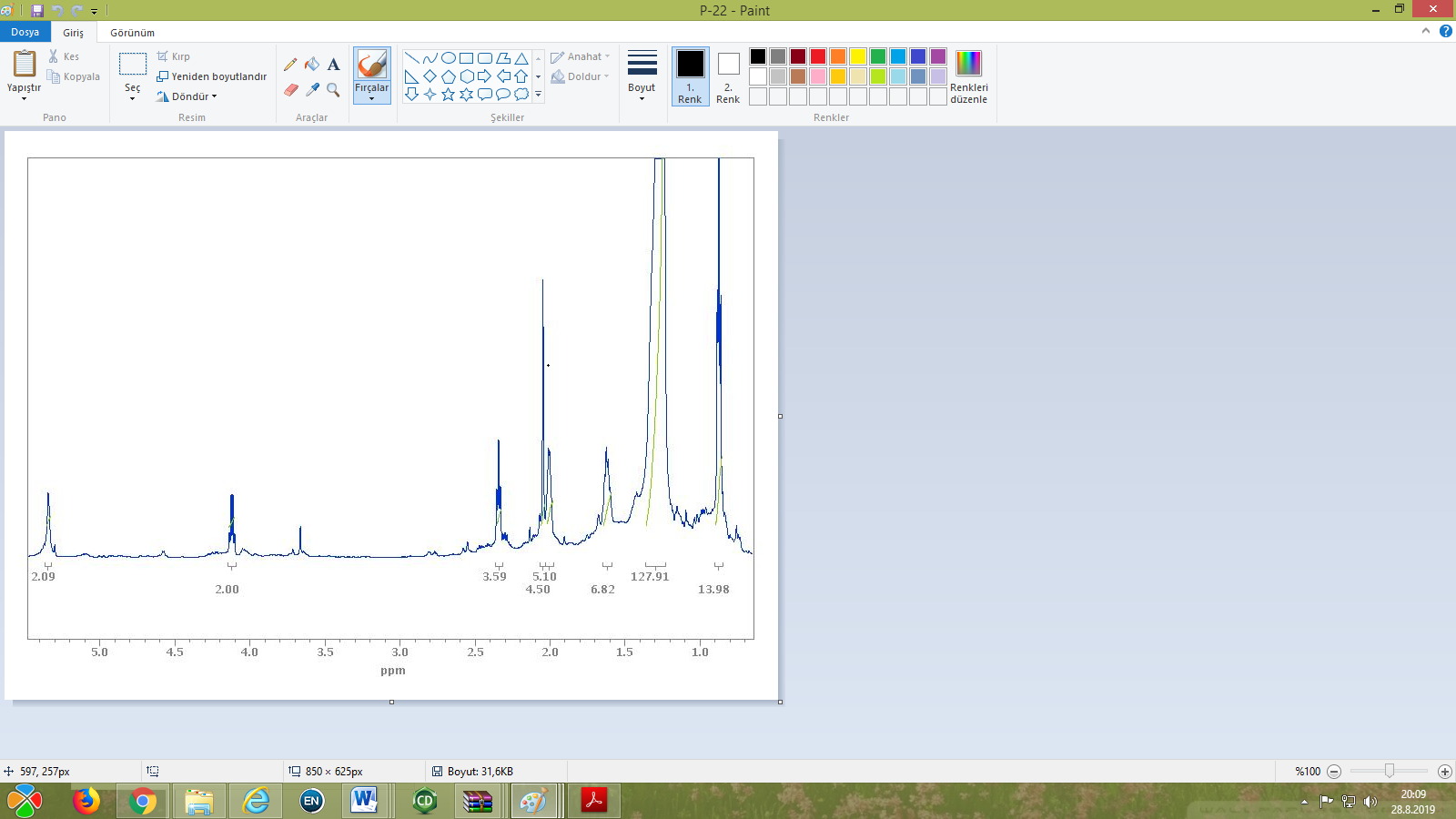


**Figure S 3.** ^1^H-NMR spectrum of butylpalmitate obtained from Fraction 2.2.


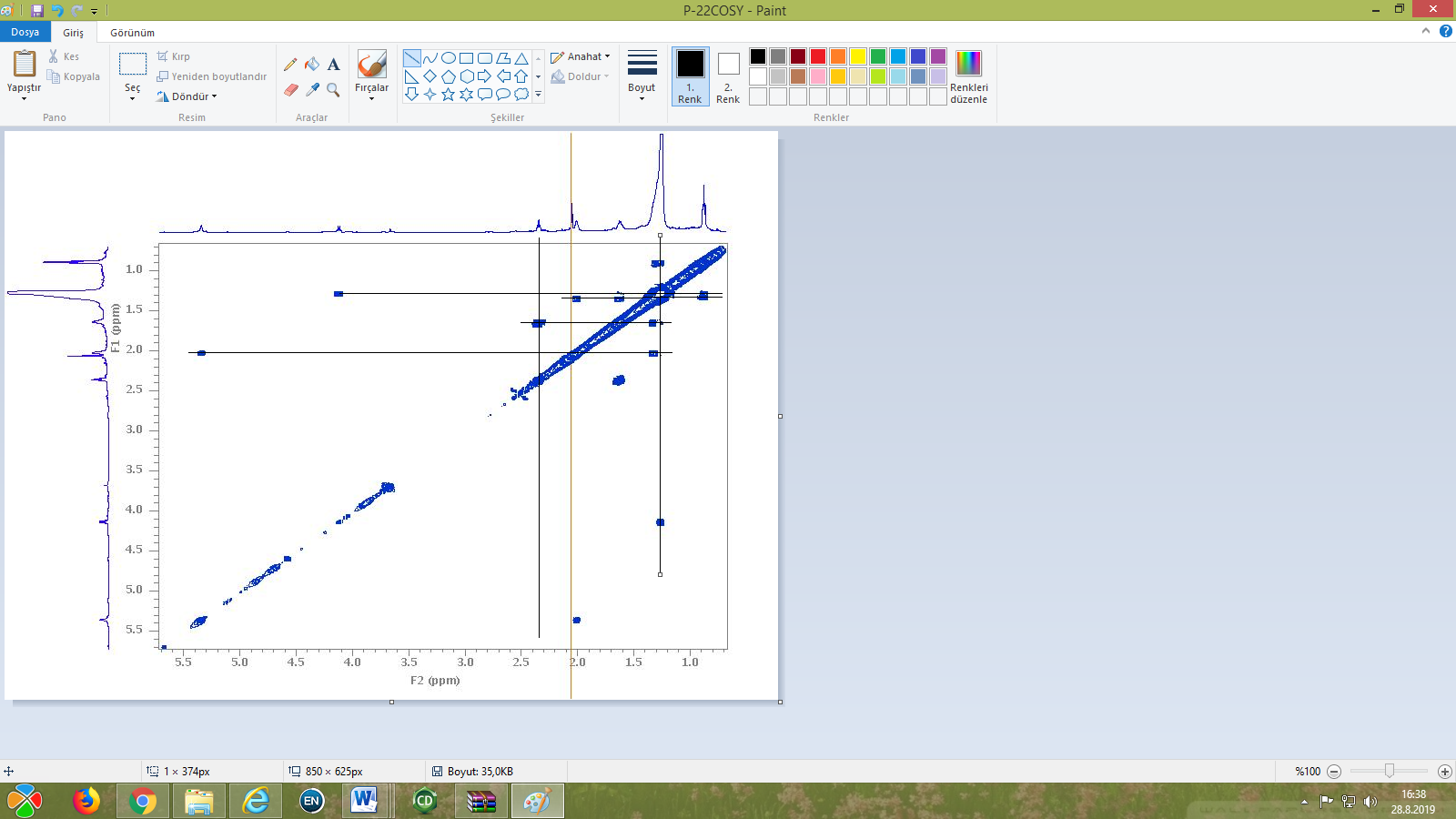


**Figure S 4.** COSY-NMR spectrum of Fraction 2.2.


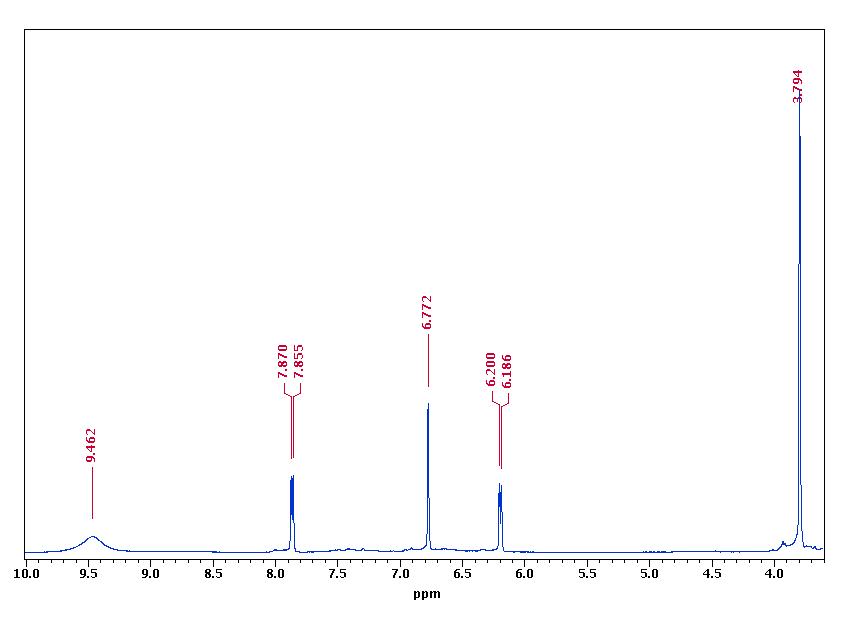


**^1^H-NMR 600 MHz**


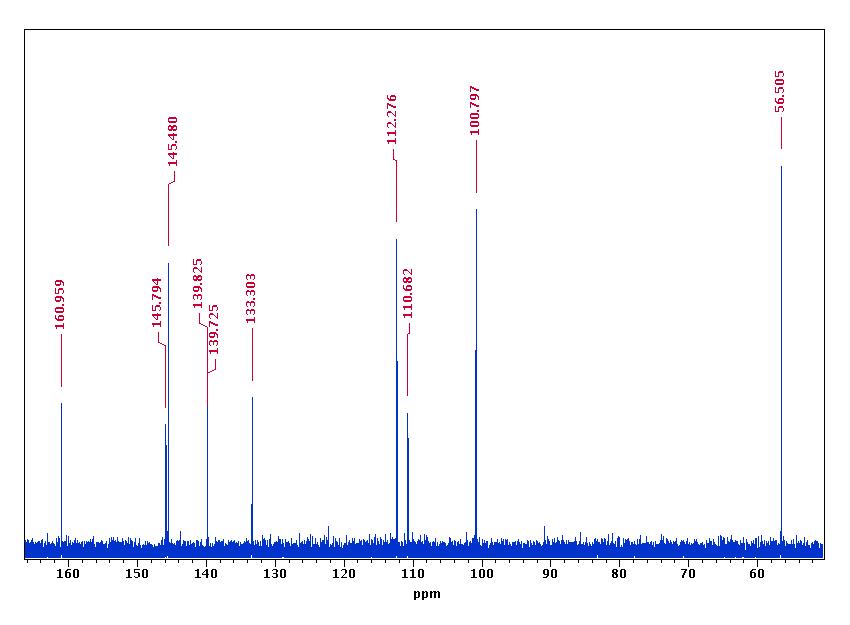


**^13^C-NMR 150 MHz**


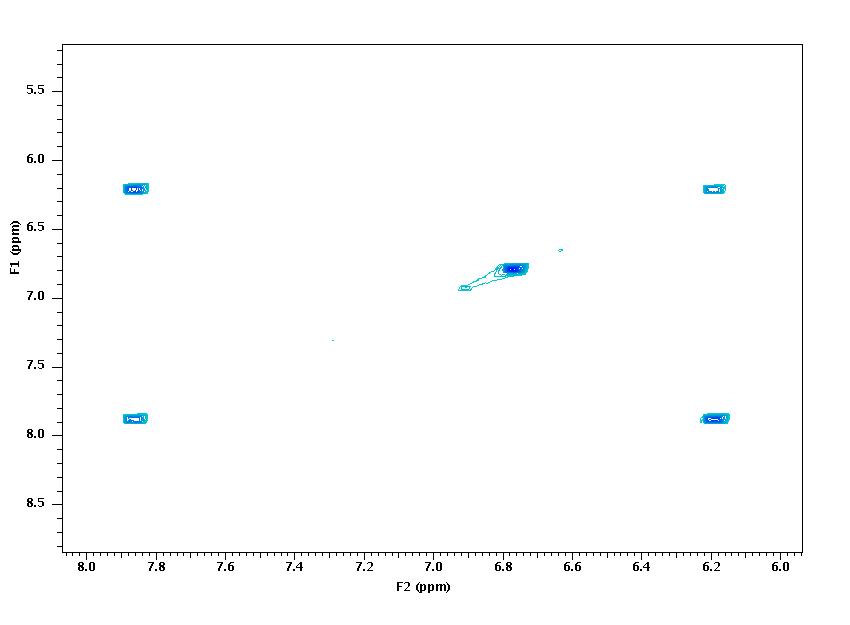


**COSY-NMR**


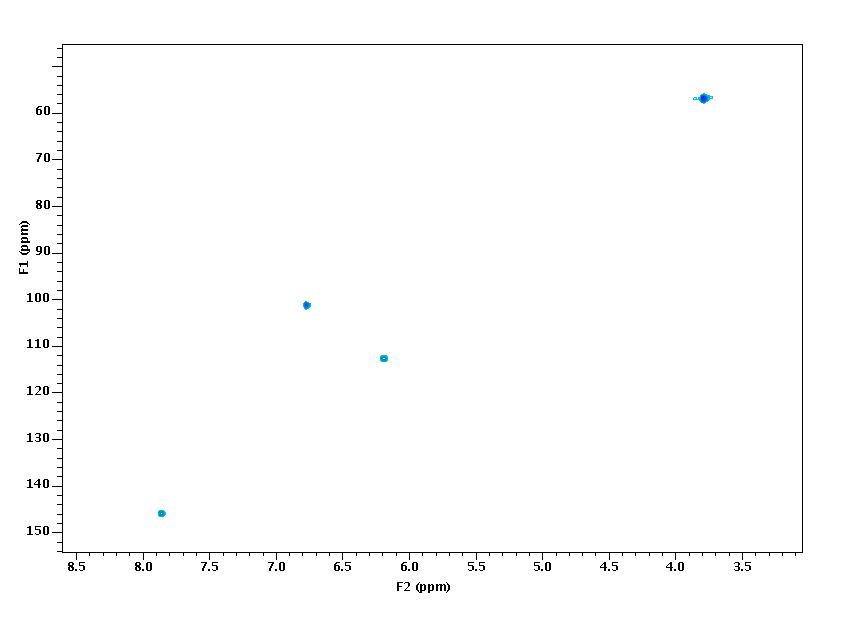


**HSQC-NMR**


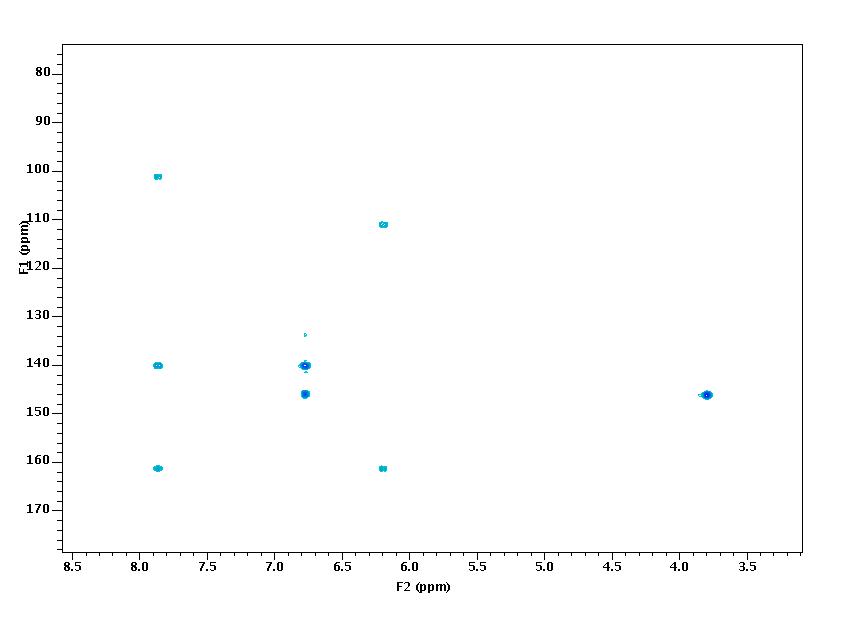


**HMBC**

**Figure S 5.** NMR spectra of fraxetin


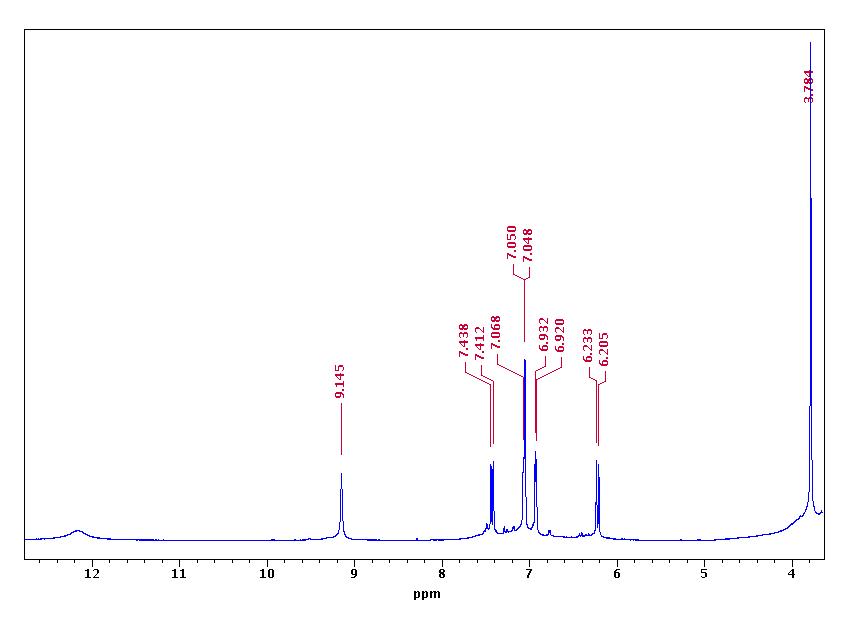


**^1^H-NMR**


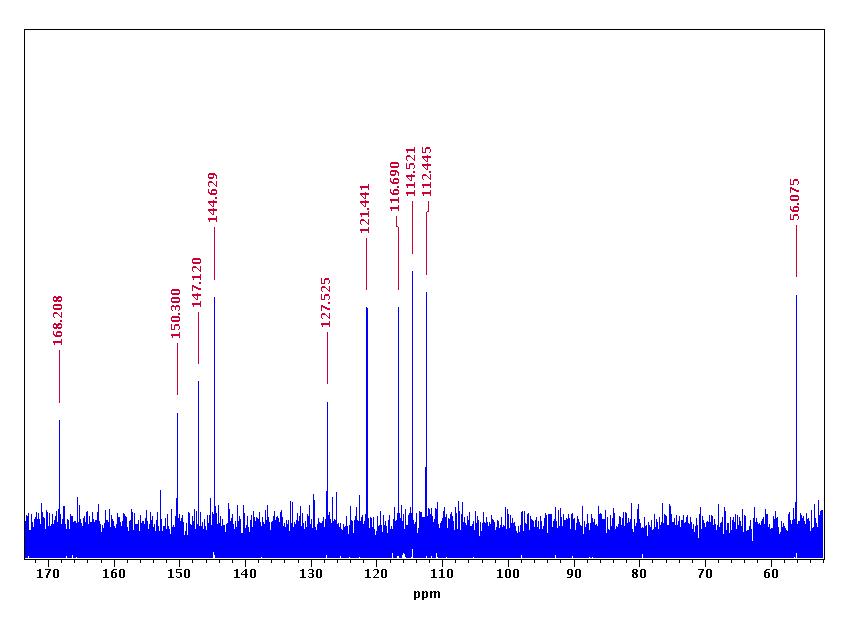


**^13^C-NMR**


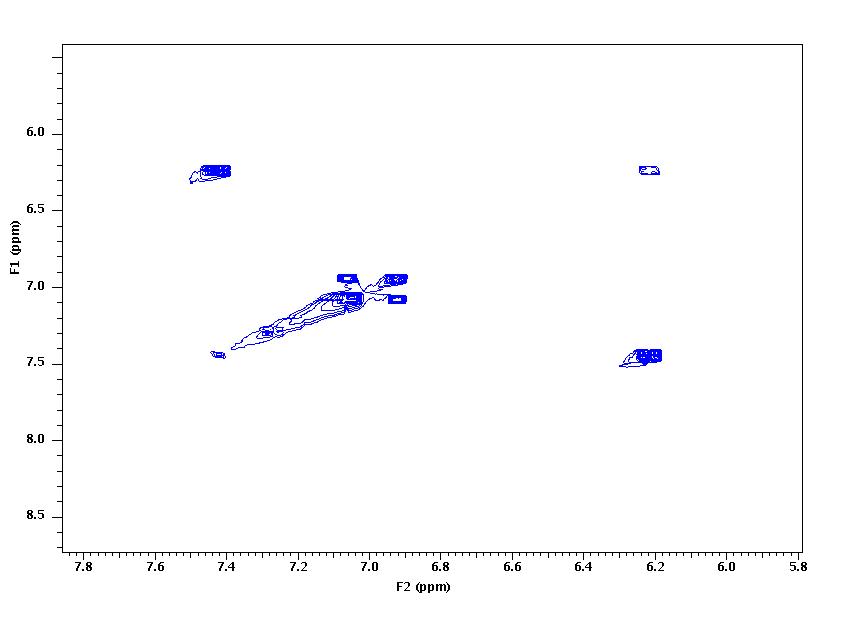


**COSY-NMR**


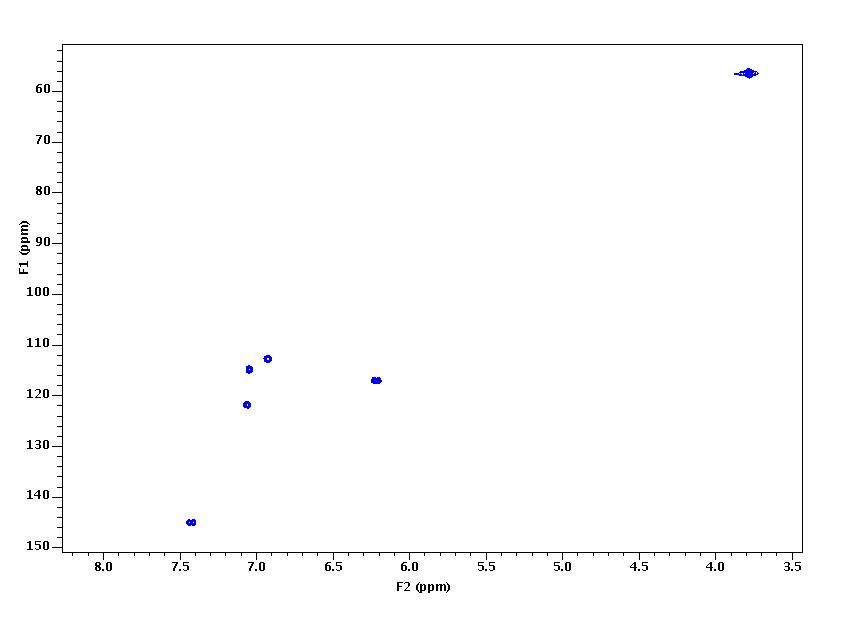


**HSQC-NMR**


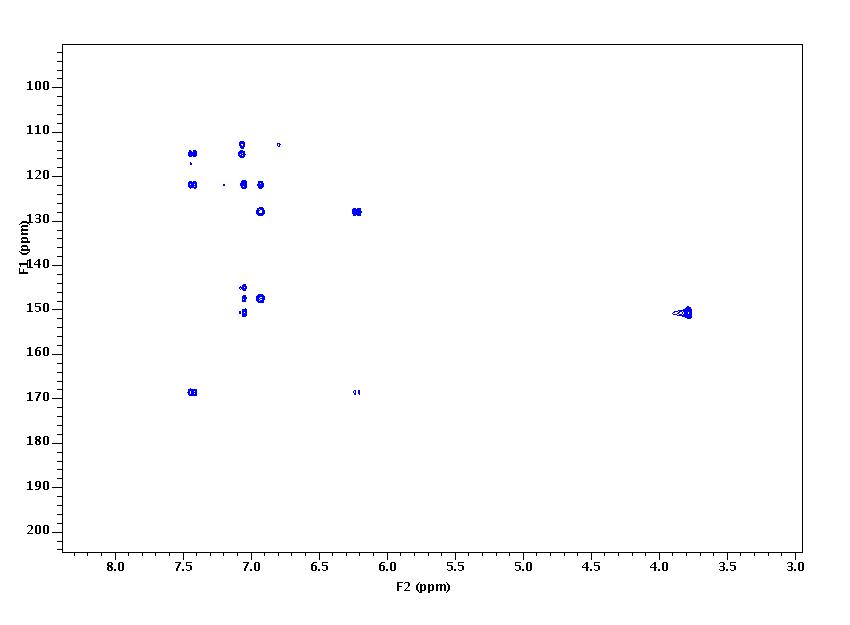


**HMBC-NMR**

**Figure S 6.** NMR spectra of ferulic acid

**
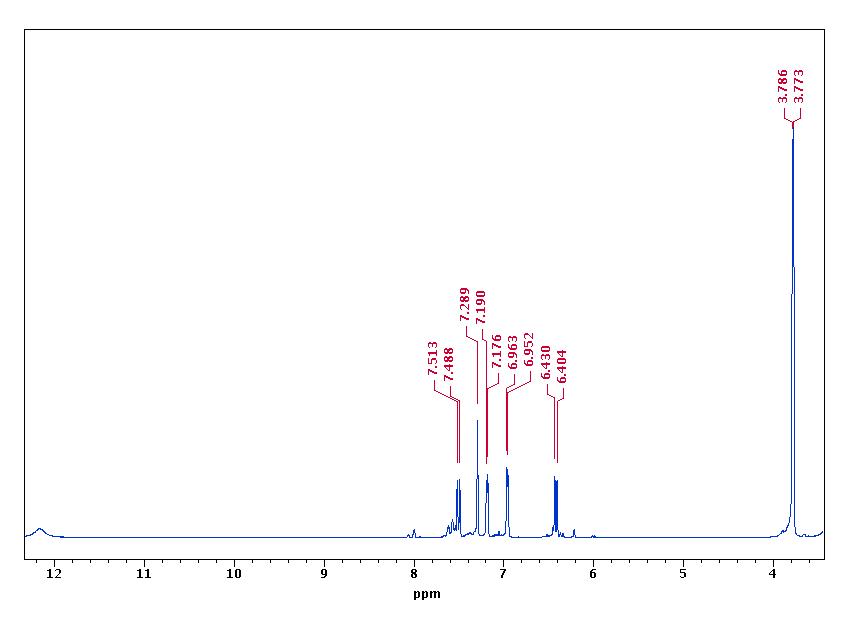
**

**^1^H-NMR 600 MHz**

**
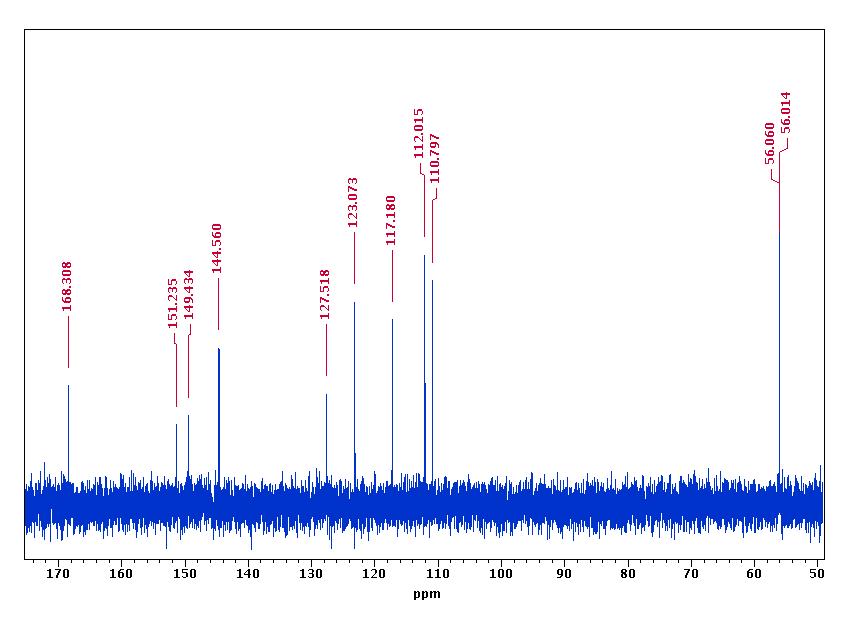
**

**^13^C-NMR 150 MHz**

**
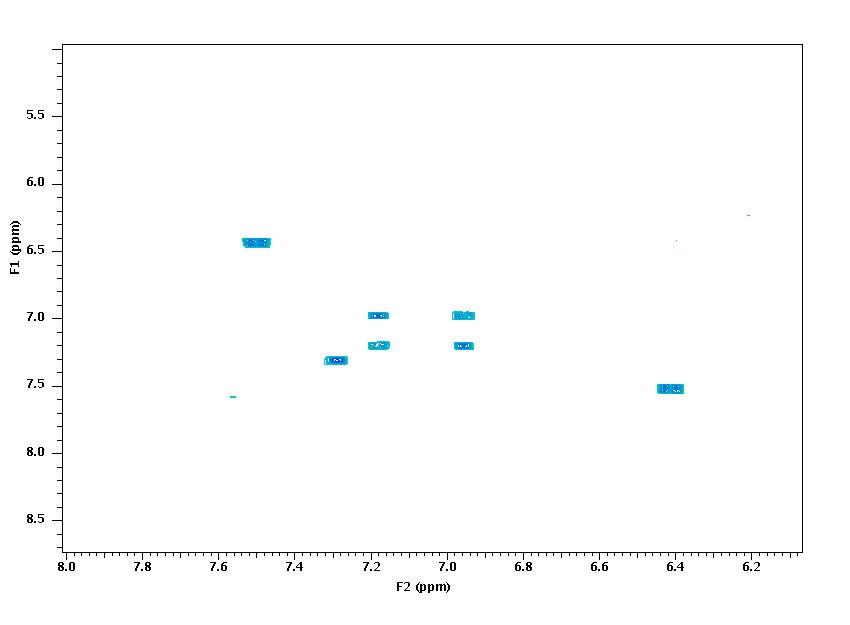
**

**COSY-NMR**

**
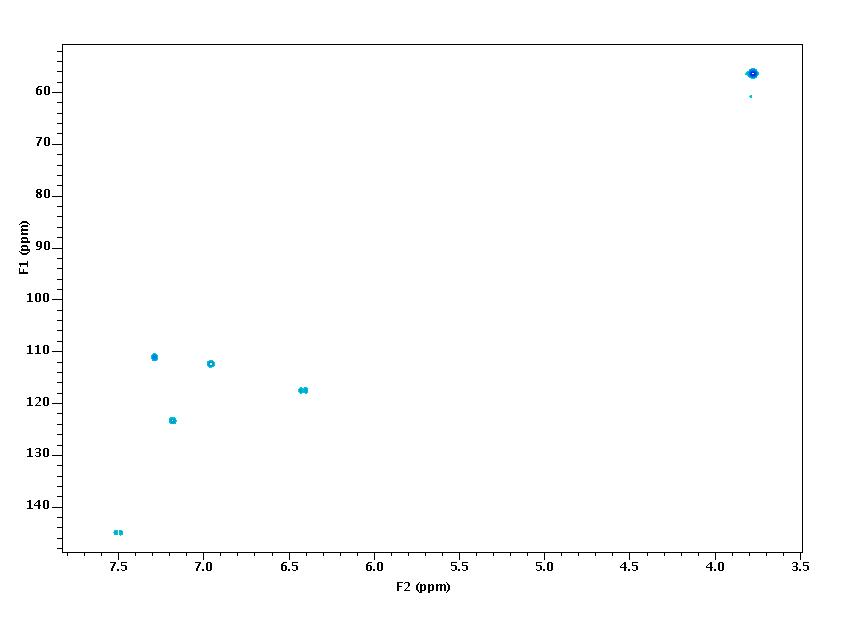
**

**HSQC-NMR**

**
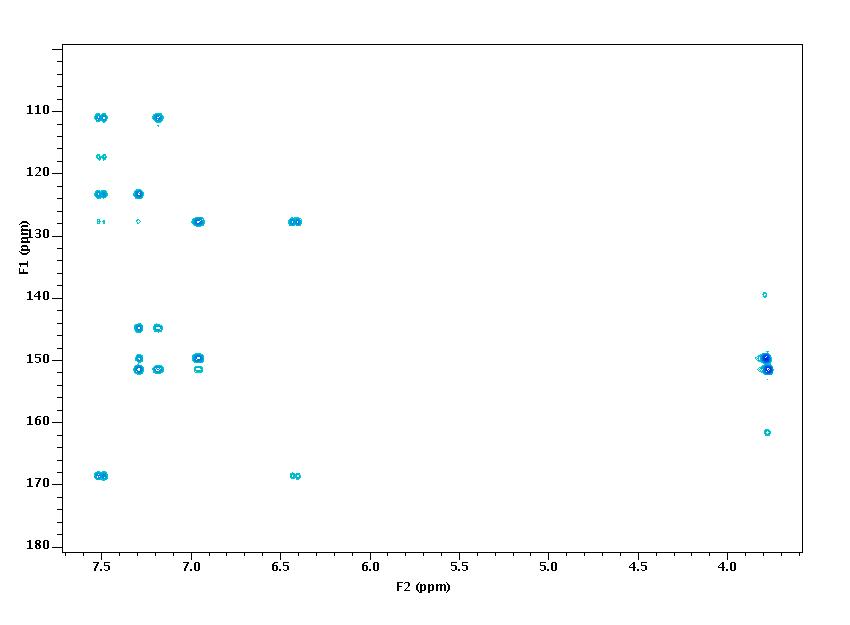
**

**HMBC**

**Figure S 7.** NMR spectra of 3,4-dimethoxy cinnamic acid


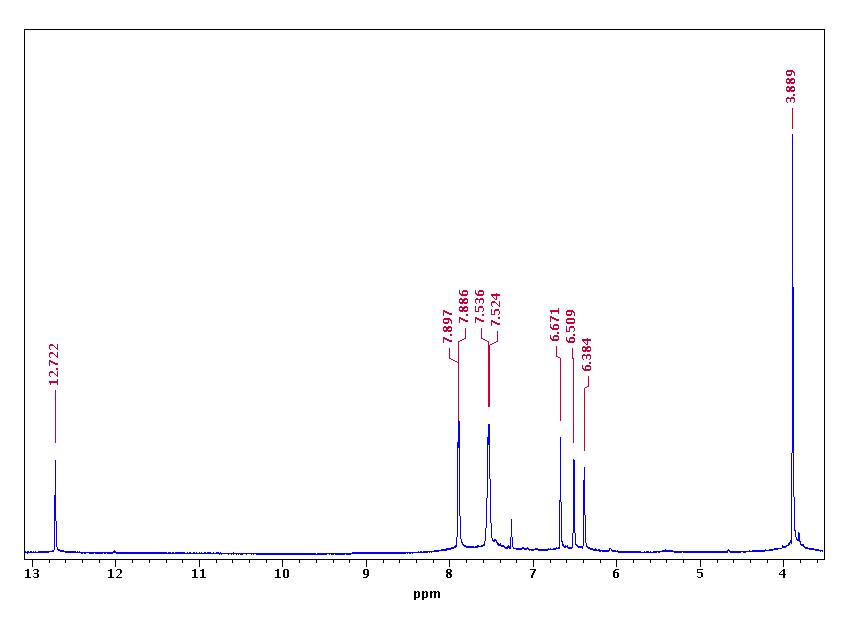


**^1^H-NMR**


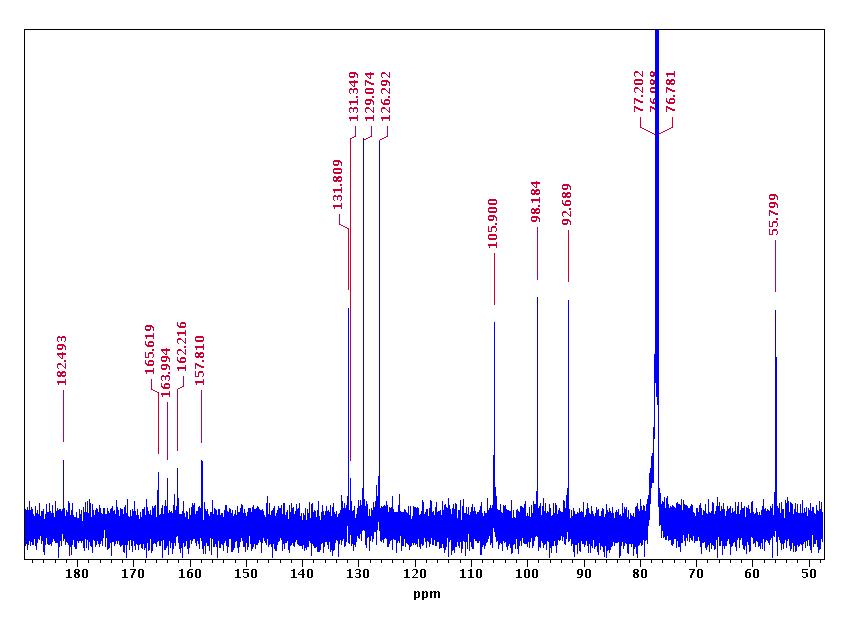


**^13^C-NMR**


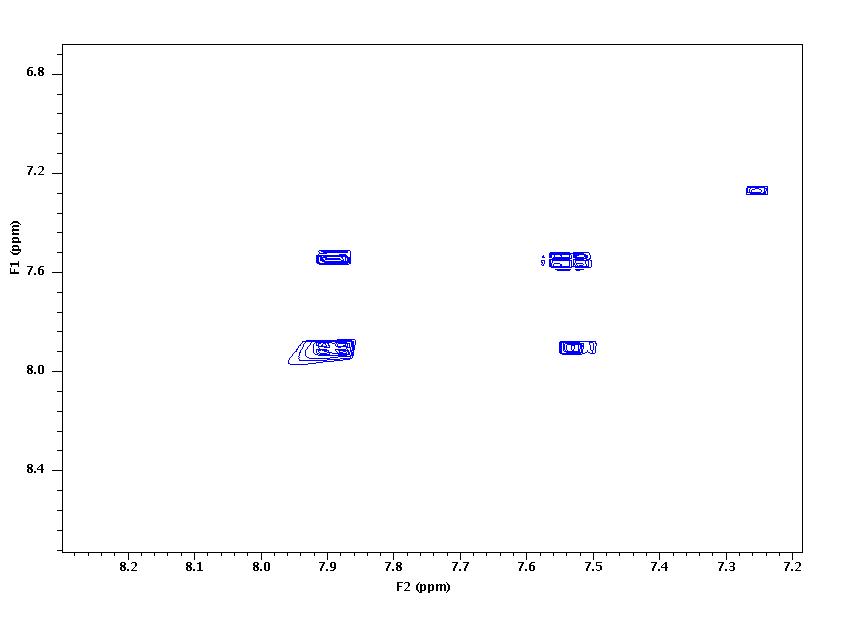


**COSY-NMR**


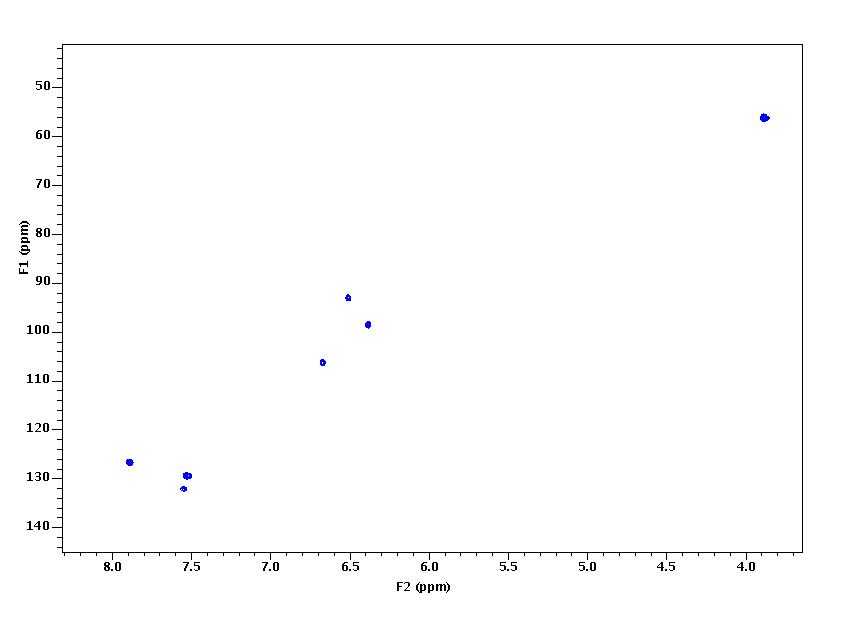


**HSQC-NMR**


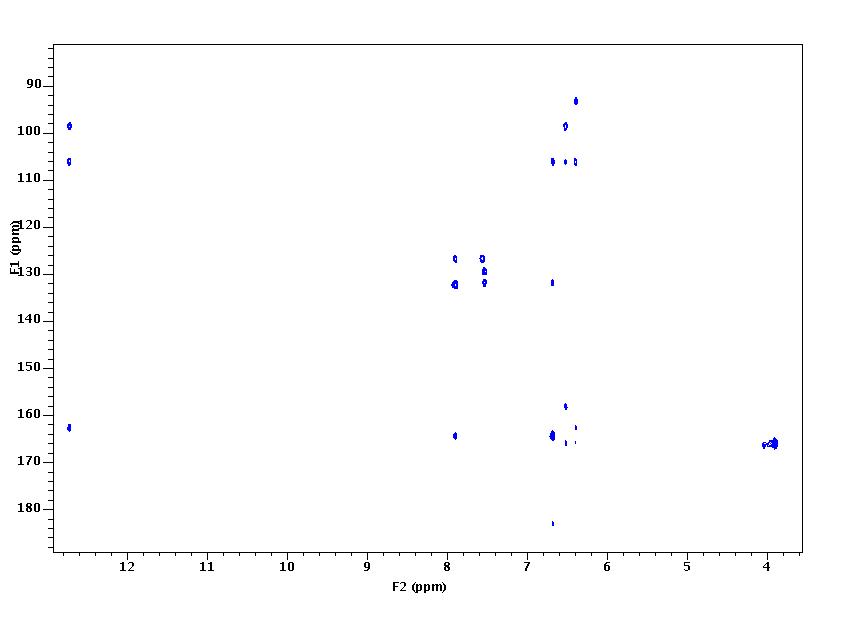


**HMBC-NMR**

**Figure S 8.** NMR spectra of tectochrysin


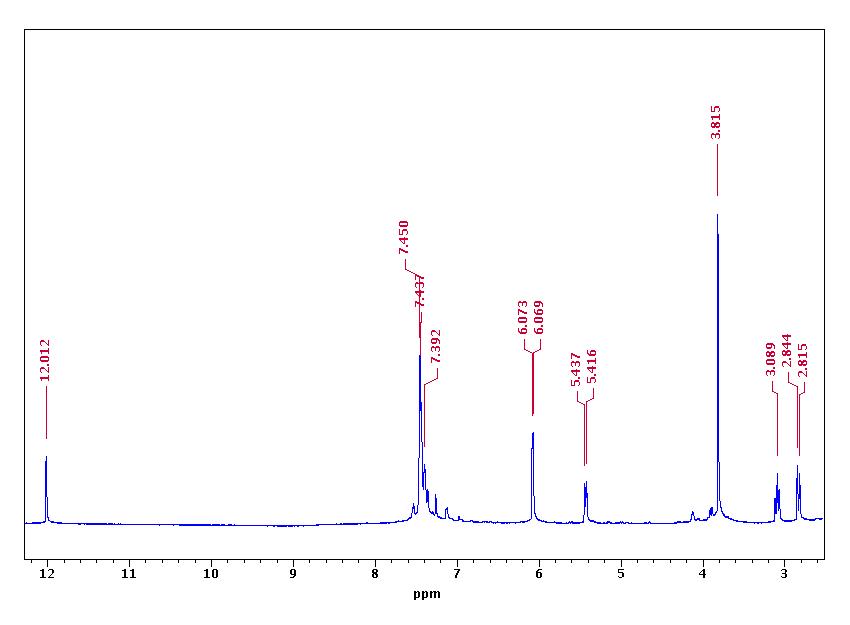


**^1^H-NMR**


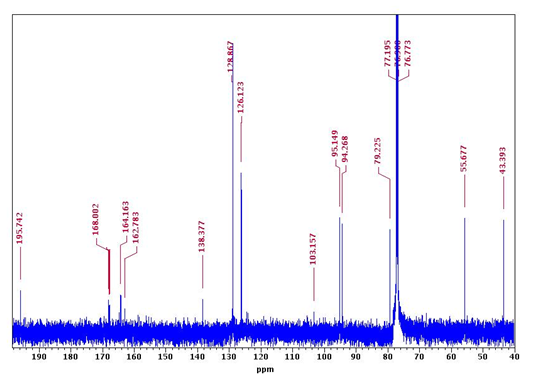


**^13^C-NMR**


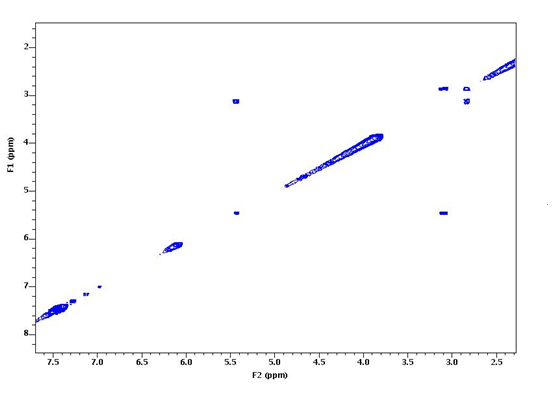


**COSY-NMR**


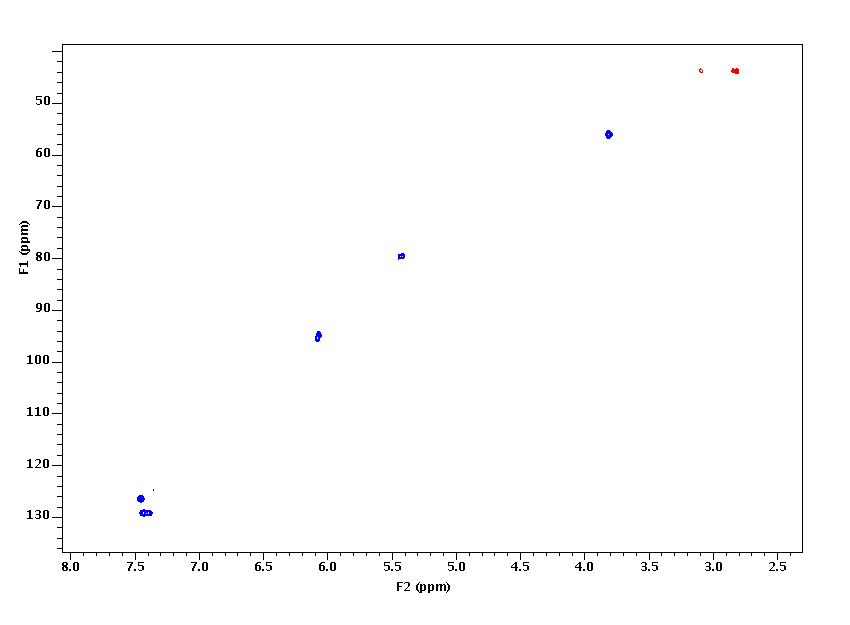


**HSQC-NMR**


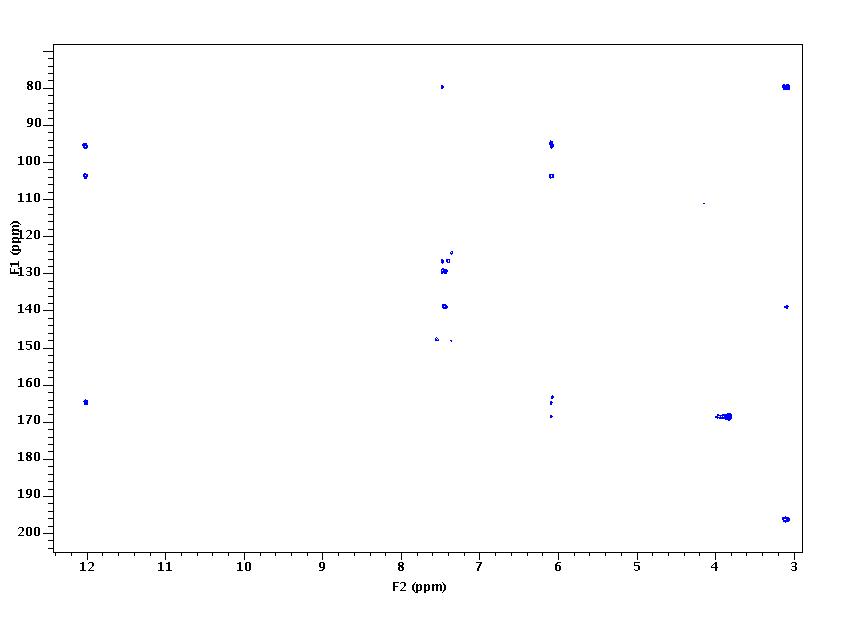


**HMBC-NMR**

**Figure S 9.** NMR spectra of pinostrobin


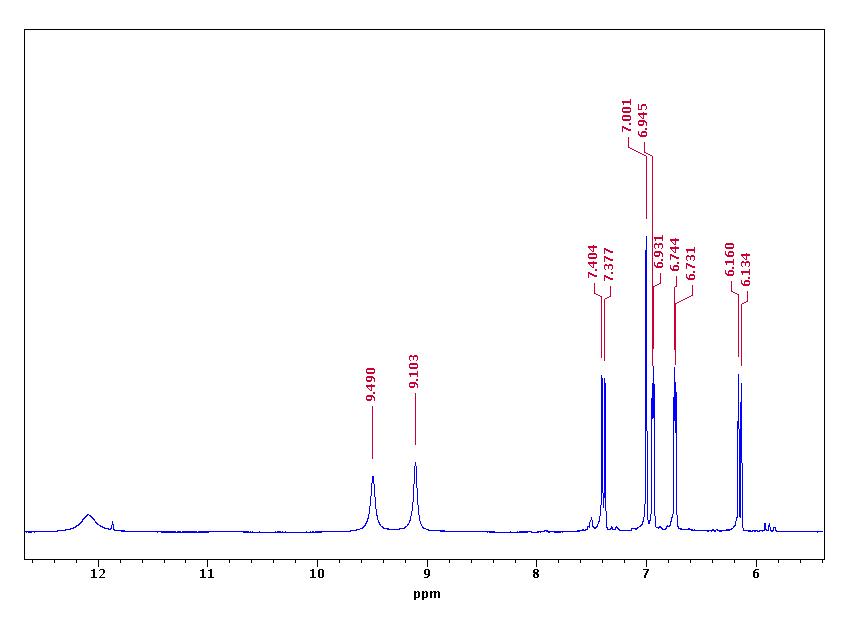


**^1^H-NMR**


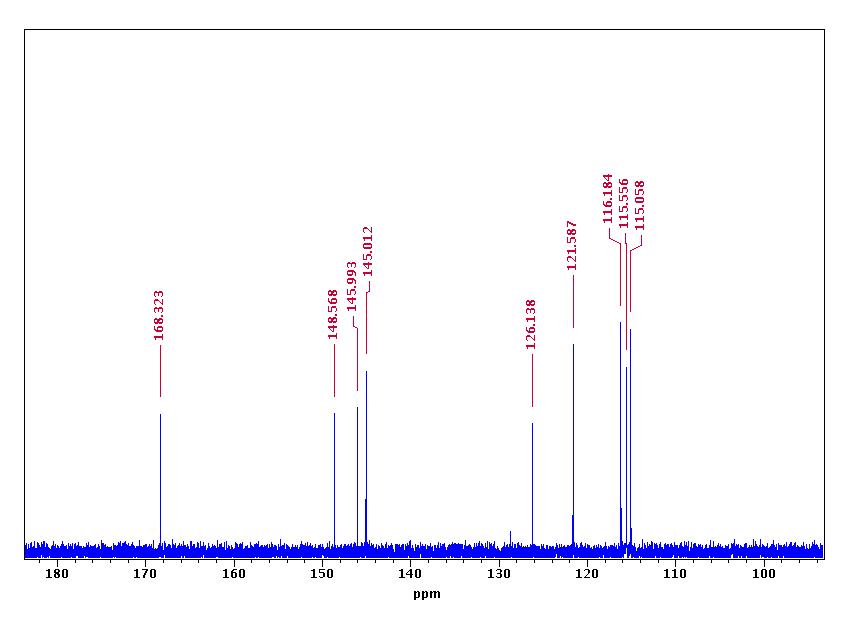


**^13^C-NMR**


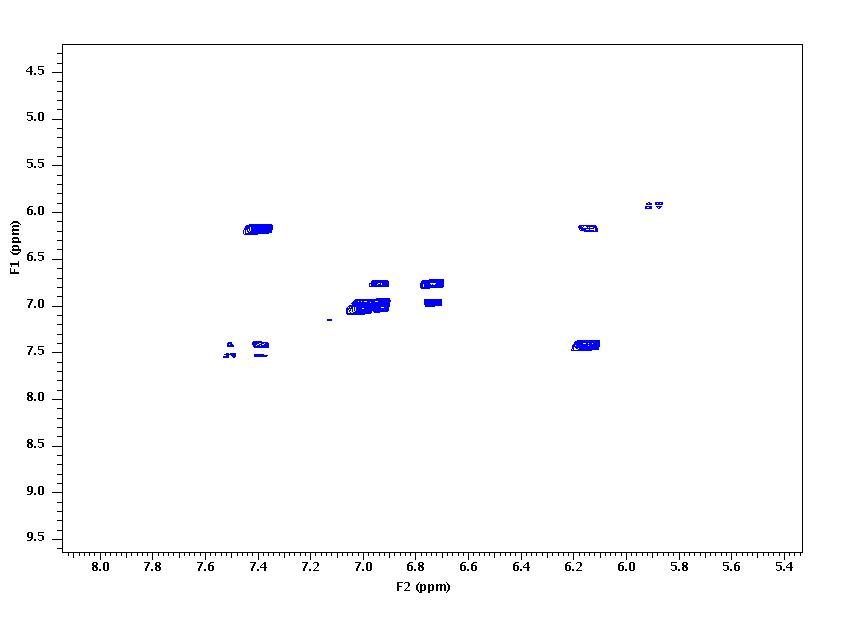


**COSY-NMR**


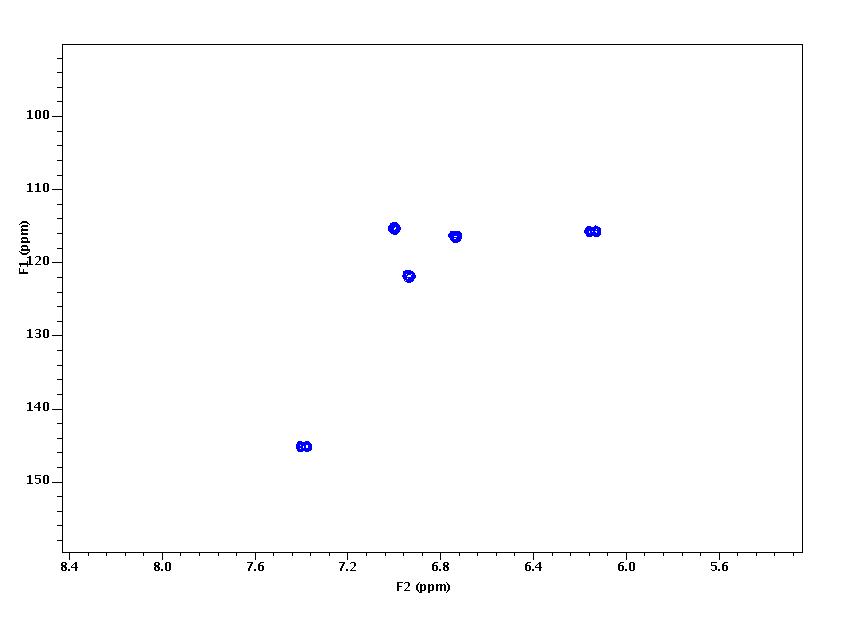


**HSQC-NMR**


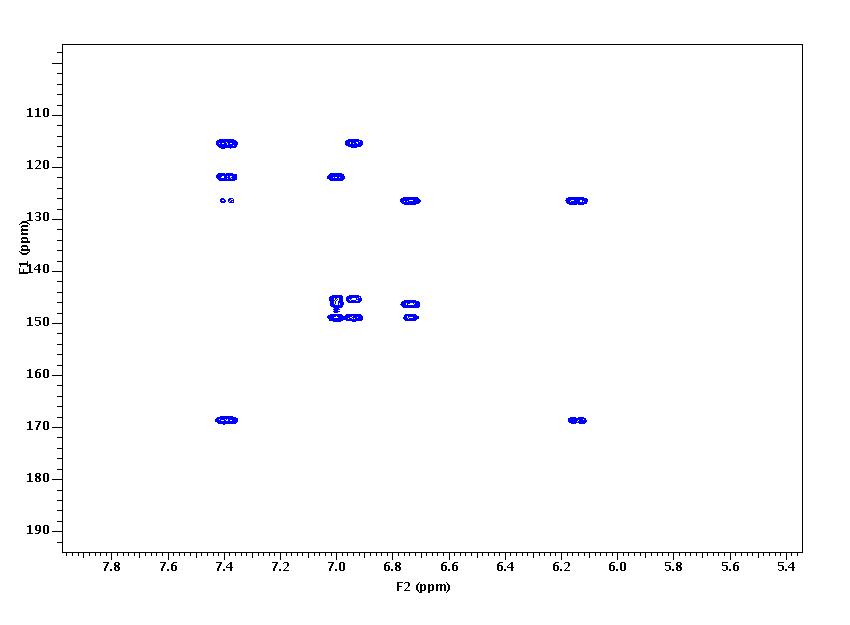


**HMBC-NMR**

**Figure S 10.** NMR spectra of caffeic acid

**
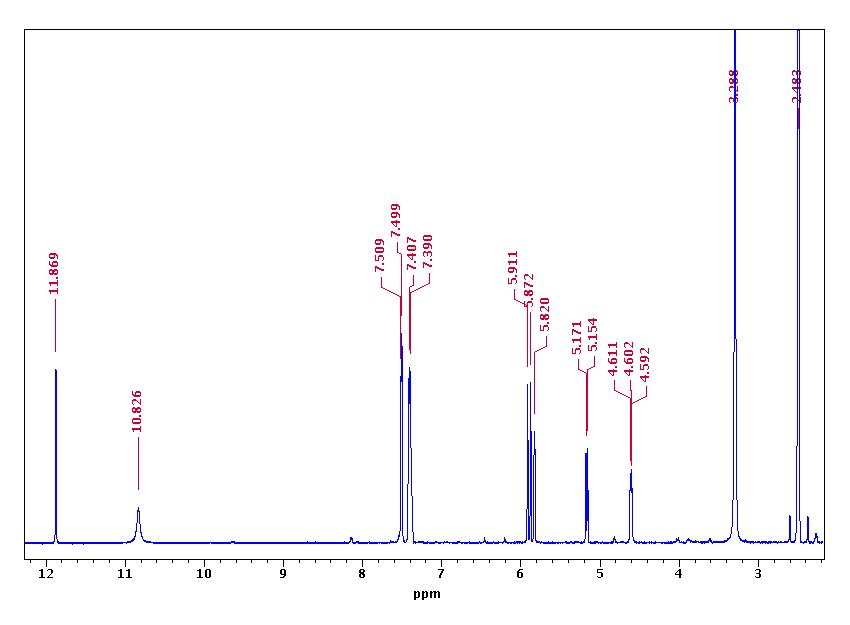
**

**^1^H-NMR 600 MHz**

**
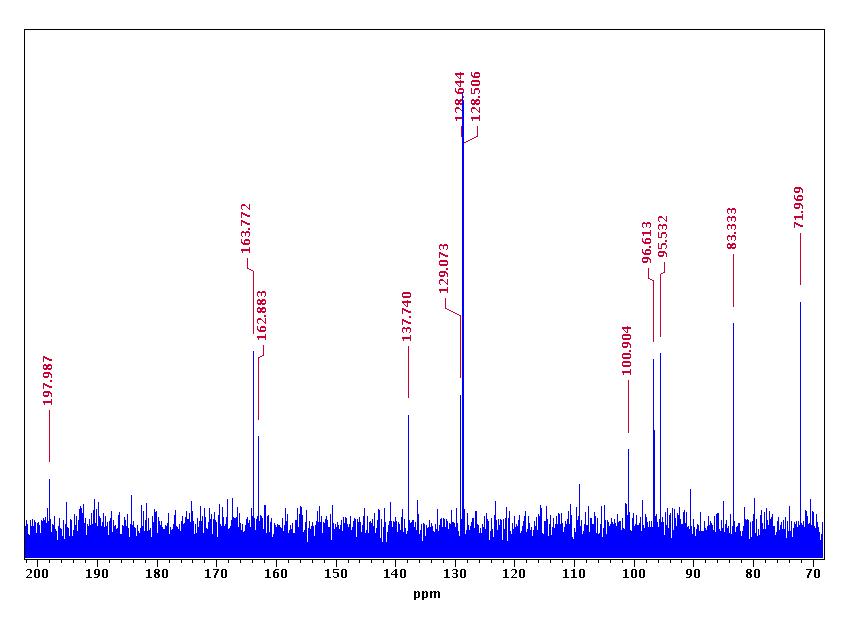
**

**^13^C-NMR 150 MHz**

**
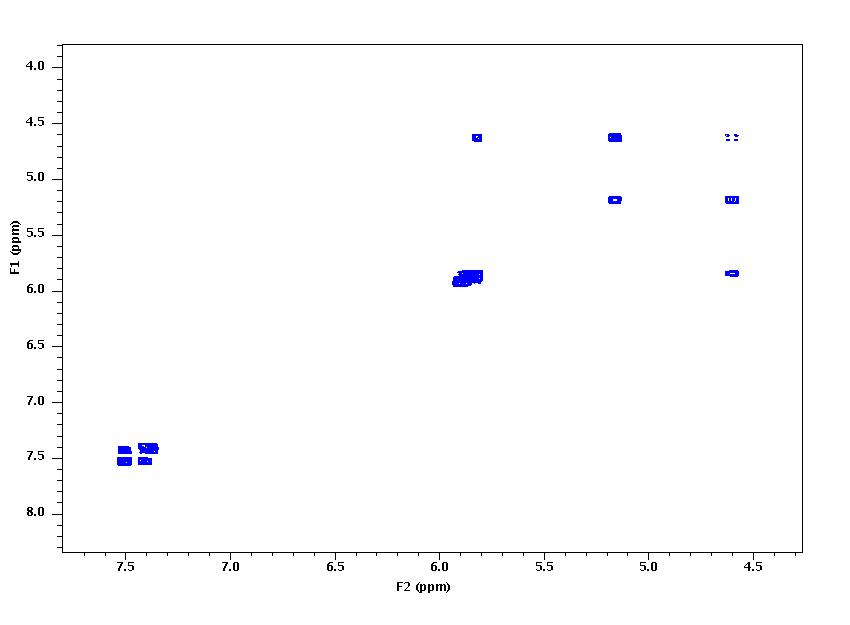
**

**COSY-NMR**

**
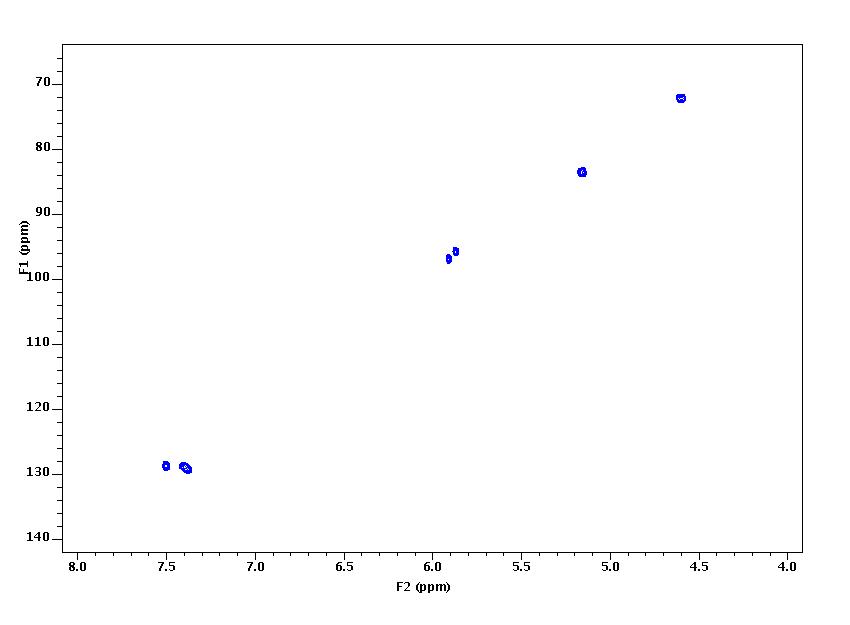
**

**HSQC-NMR**

**
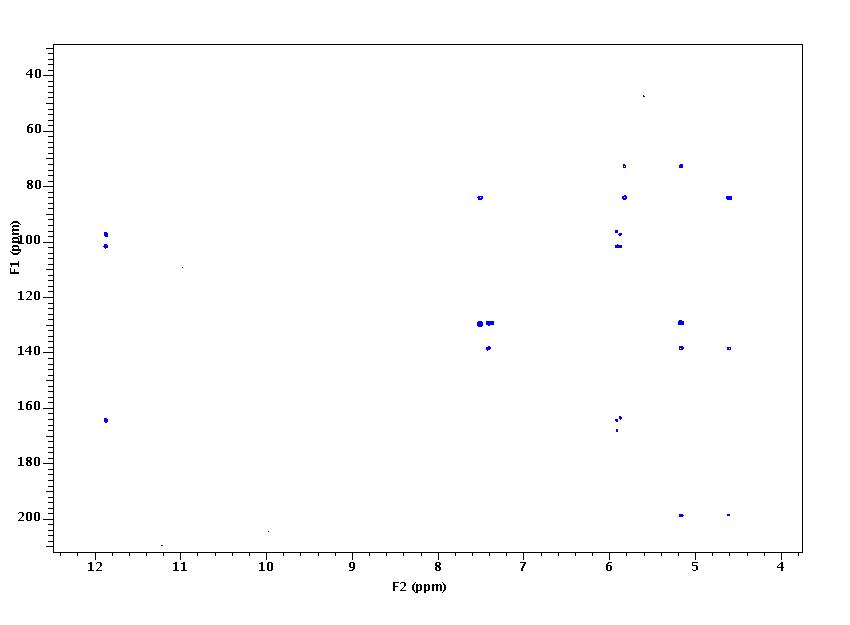
**

**HMBC-NMR**

**PINOBANKSIN**

**Figure S 11.** NMR spectra of pinobanksin

**
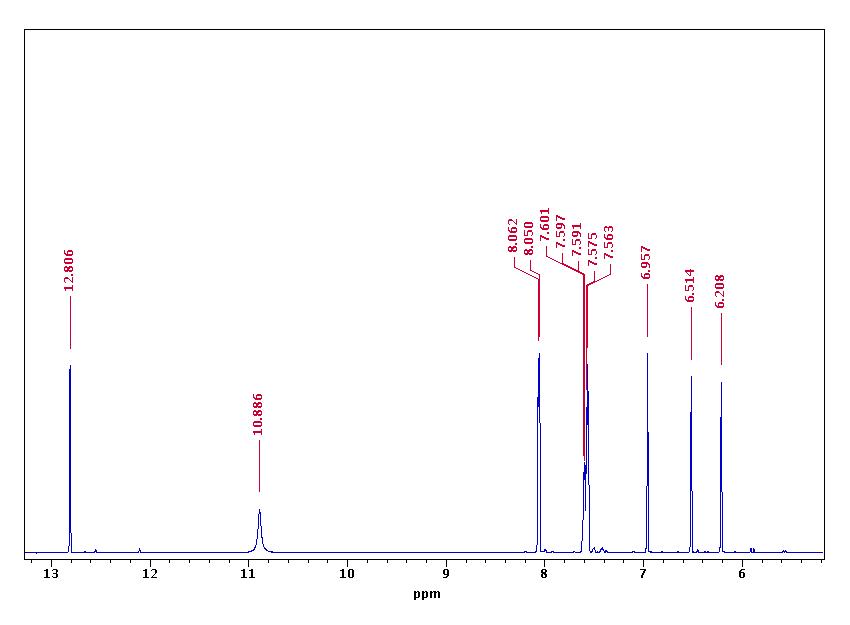
**

**^1^H-NMR 600 MHz**

**
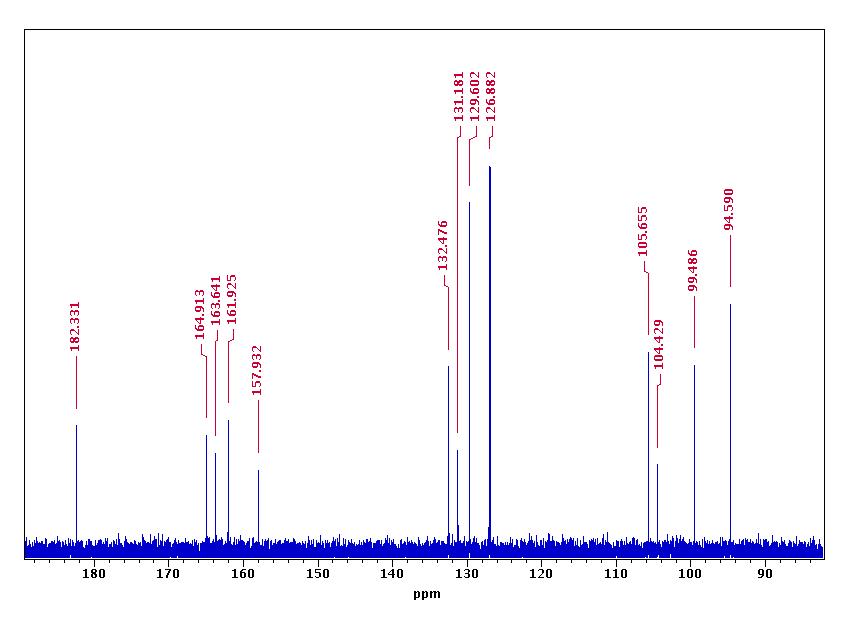
**

**^13^C-NMR 150 MHz**

**
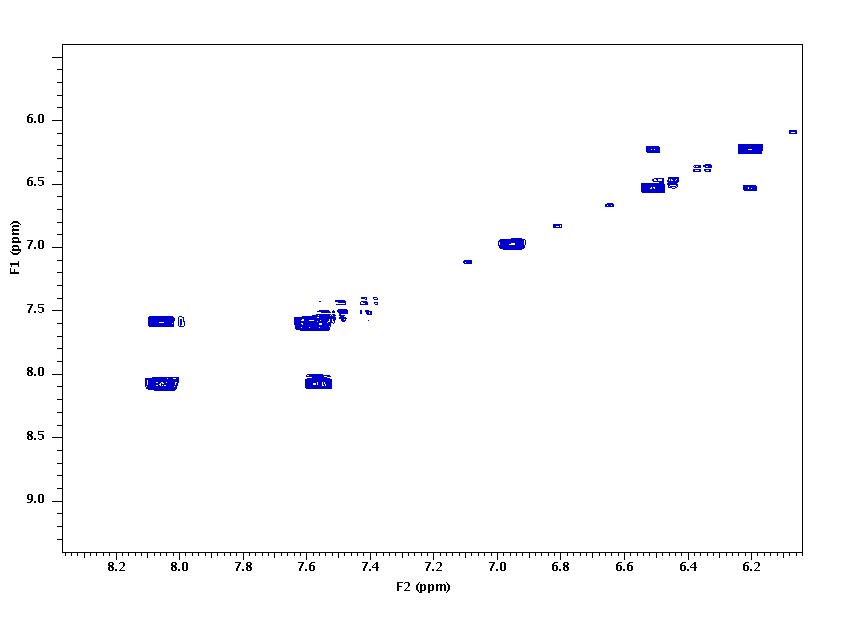
**

**COSY-NMR**

**
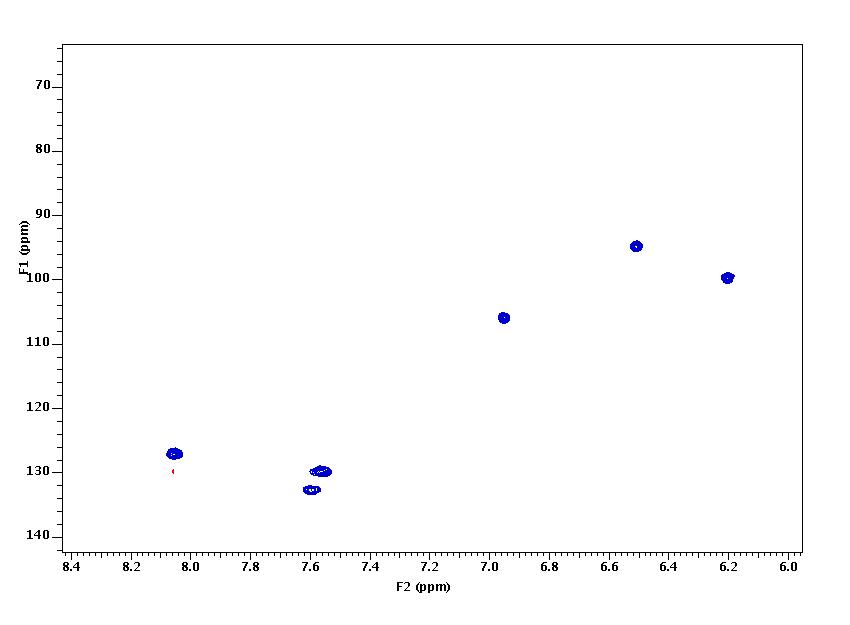
**

**HSQC-NMR**

**
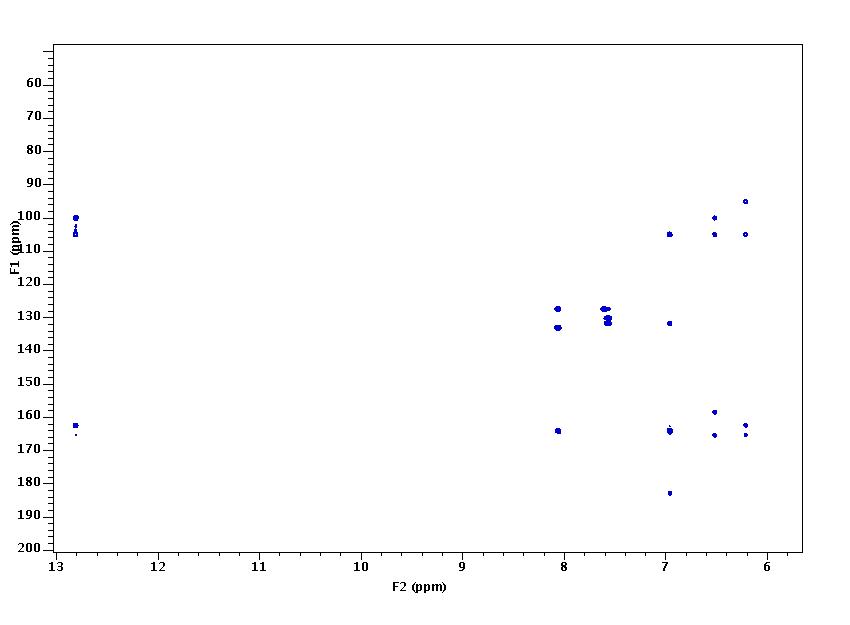
**

**HMBC-NMR**

**Figure S 12.** NMR spectra of chrysin


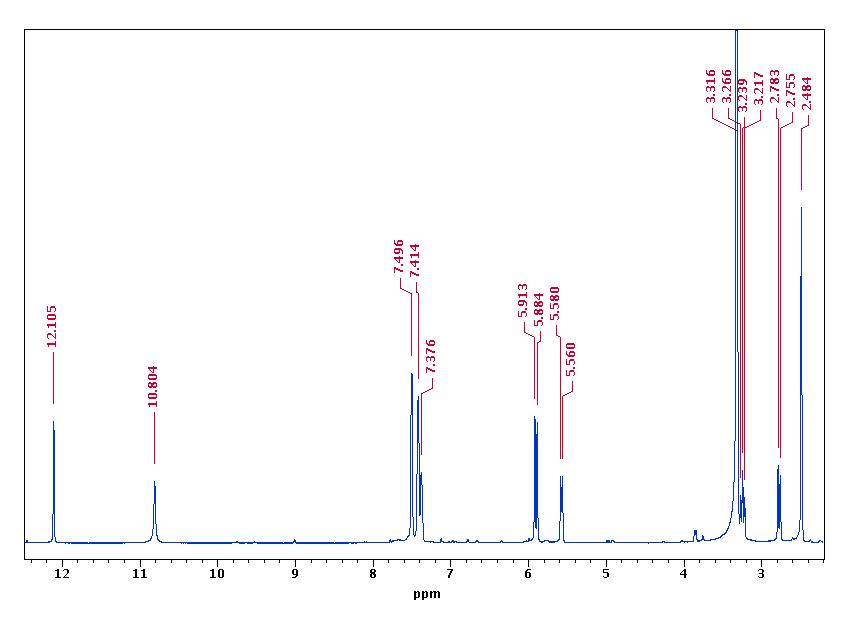


**^1^H-NMR**


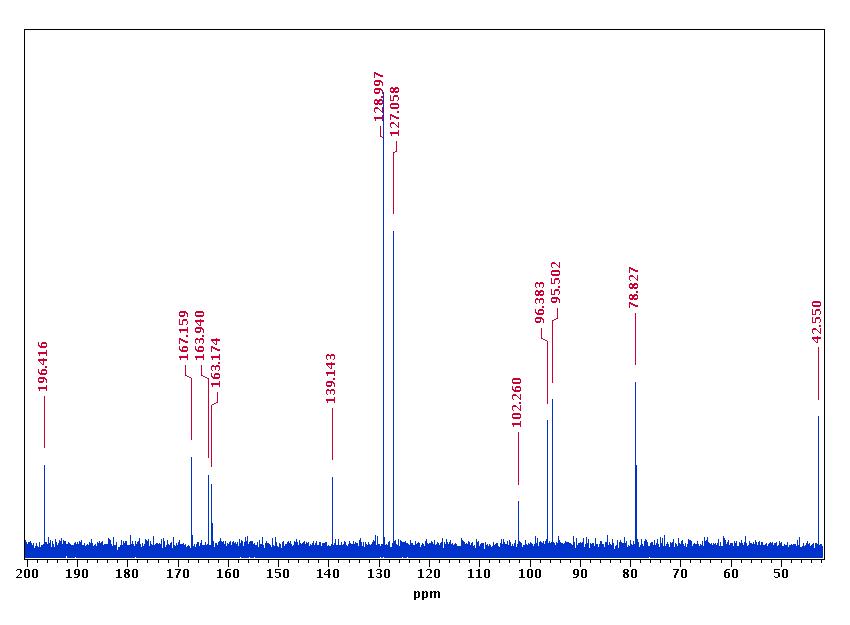


**^13^C-NMR**


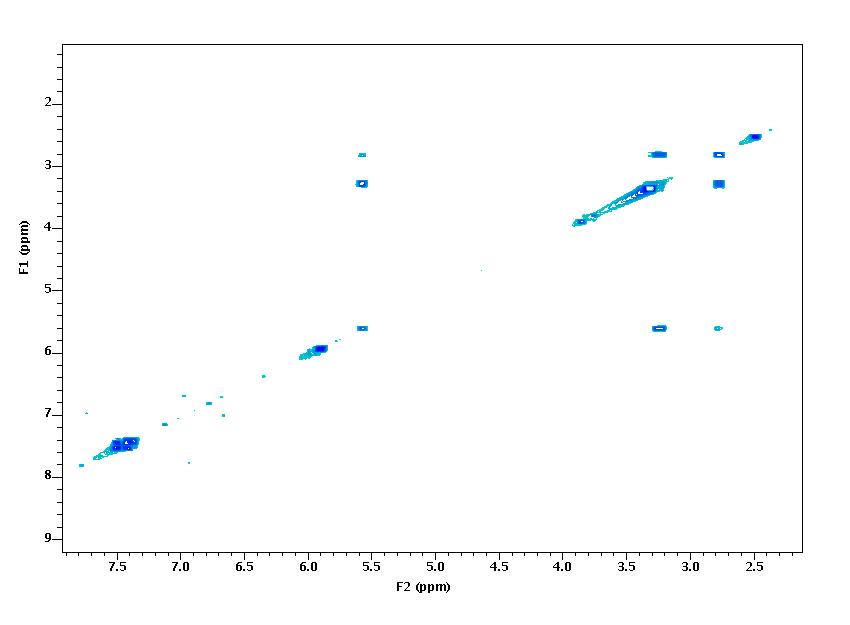


**COSY-NMR**


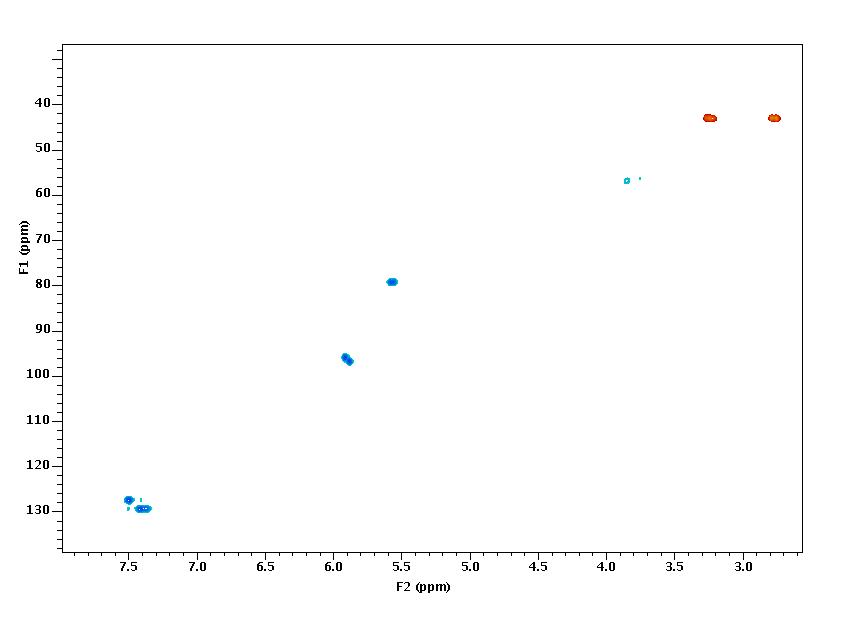


**HSQC-NMR**


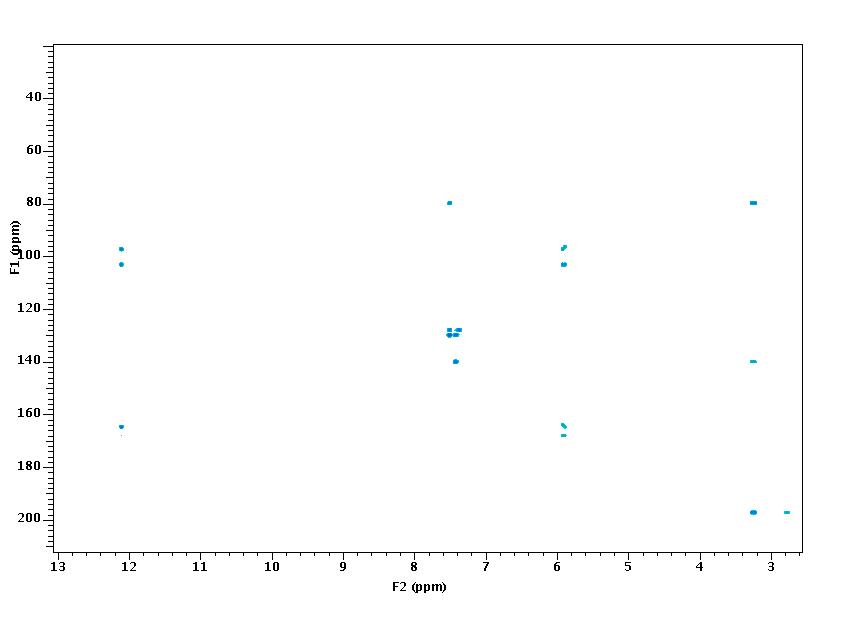


**HMBC-NMR**

**Figure S 13.** NMR spectra of pinocembrin


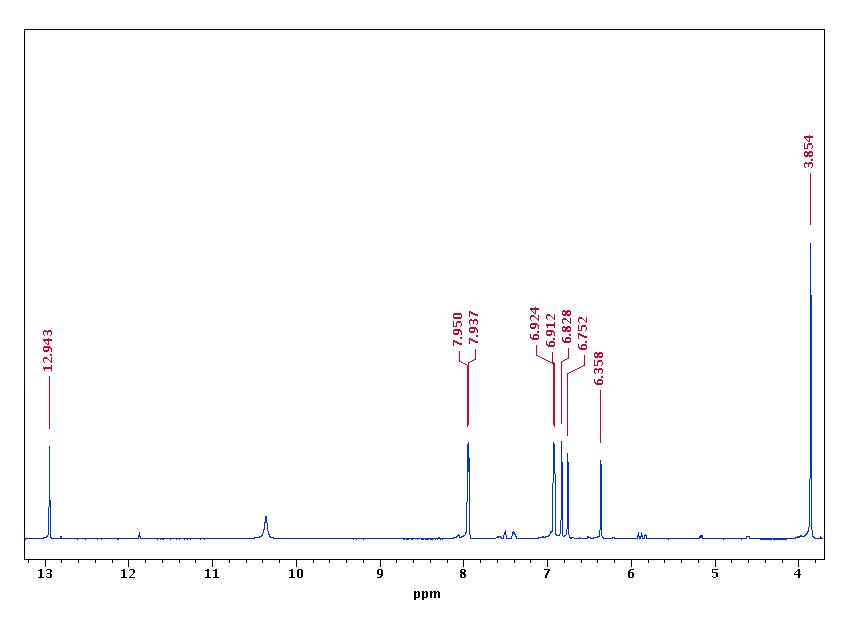


**^1^H-NMR**


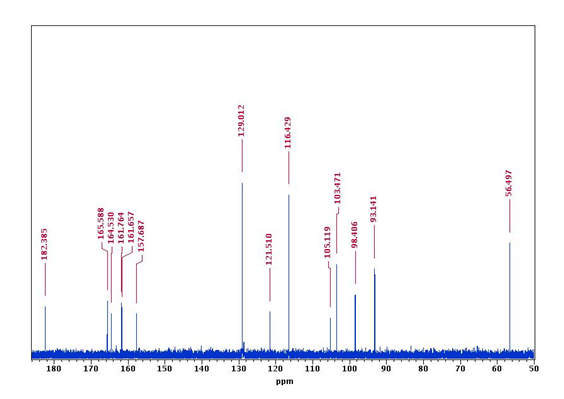


**^13^C-NMR**


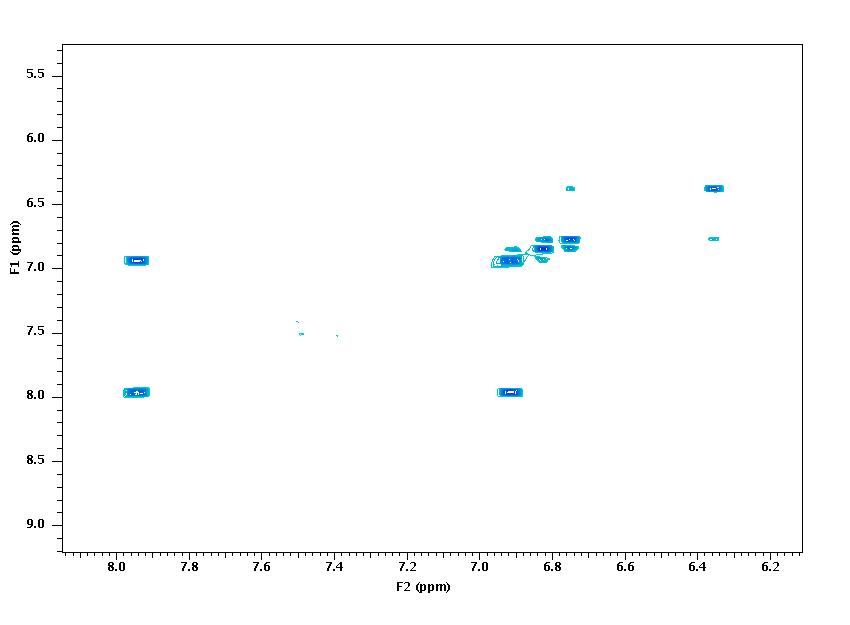


**COSY-NMR**


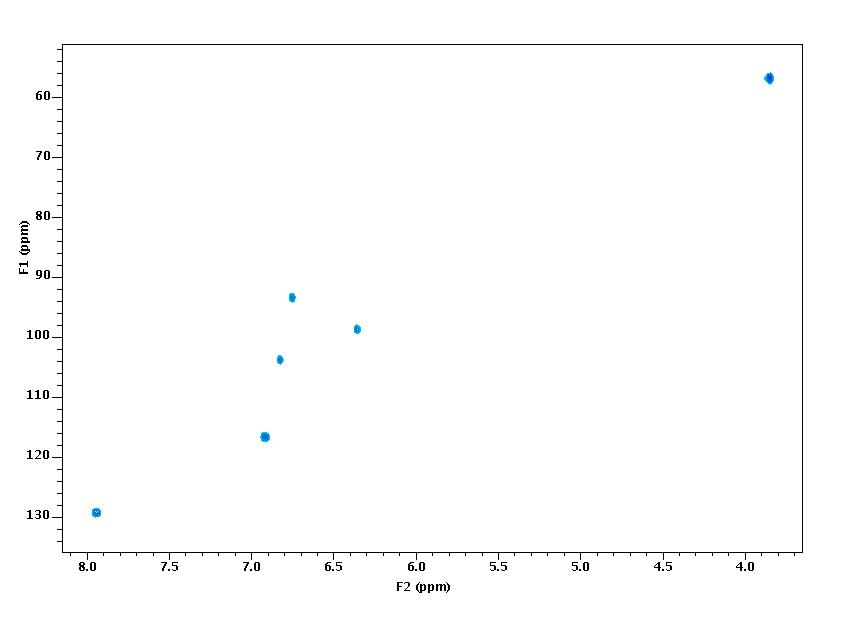


**HSQC-NMR**


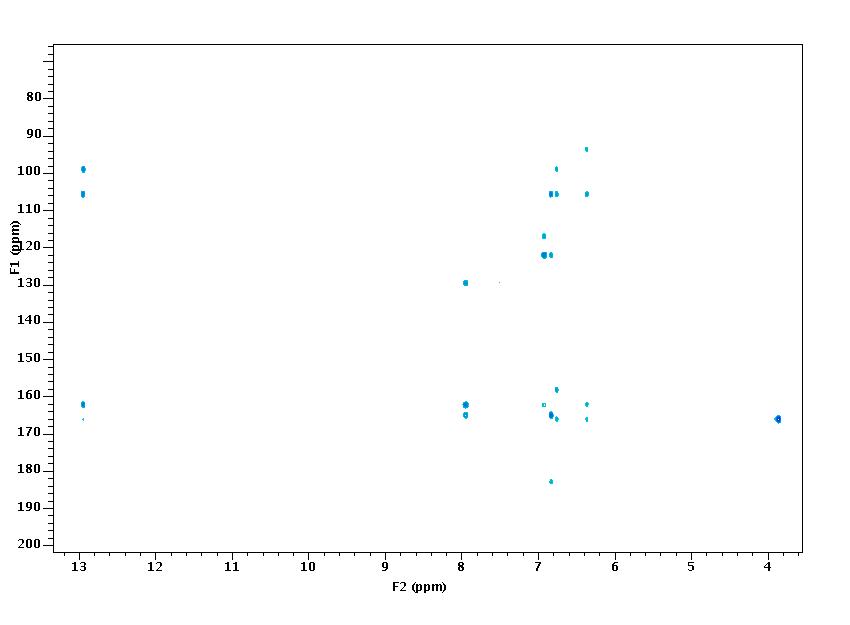


**HMBC-NMR**

**Figure S 14.** NMR spectra of genkwanin


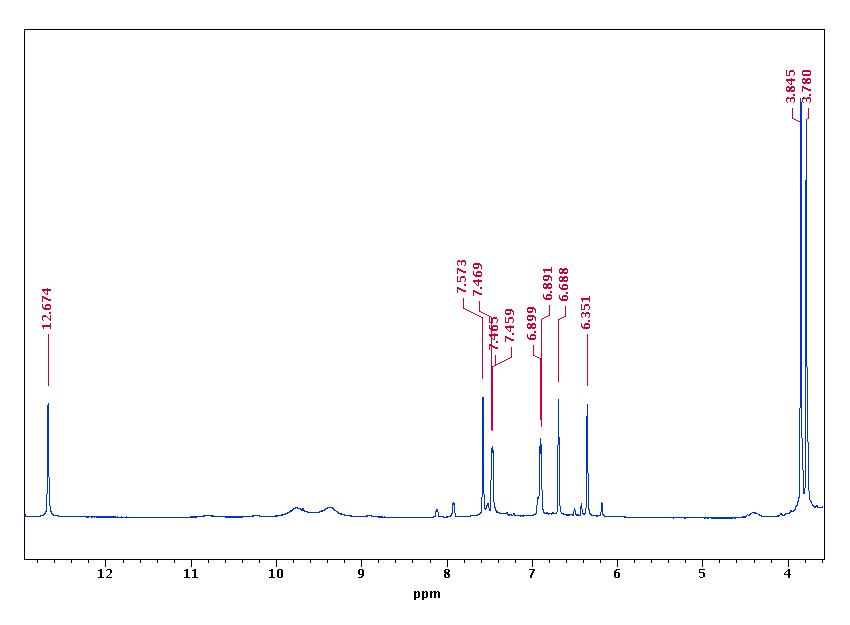


**^1^H-NMR**


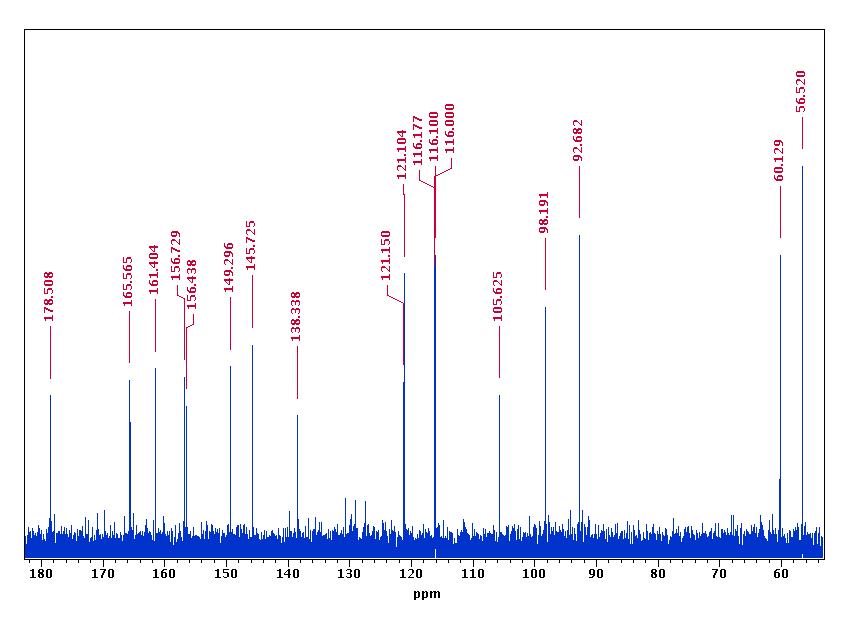


**^13^C-NMR**


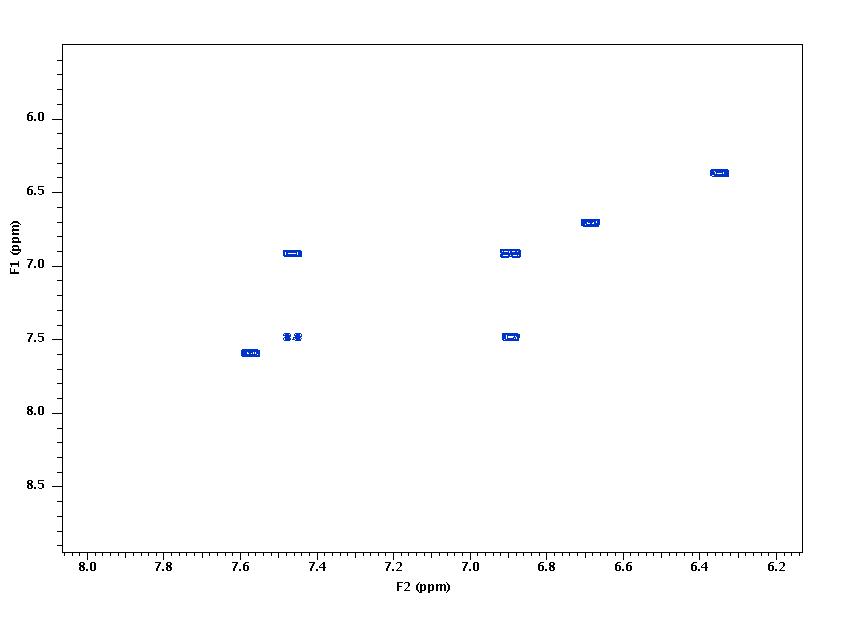


**COSY-NMR**


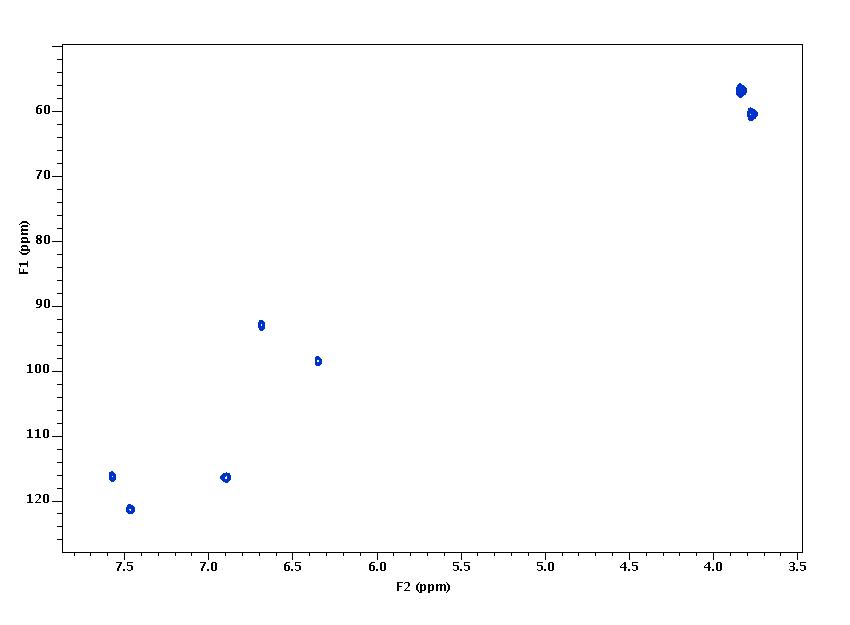


**HSQC-NMR**


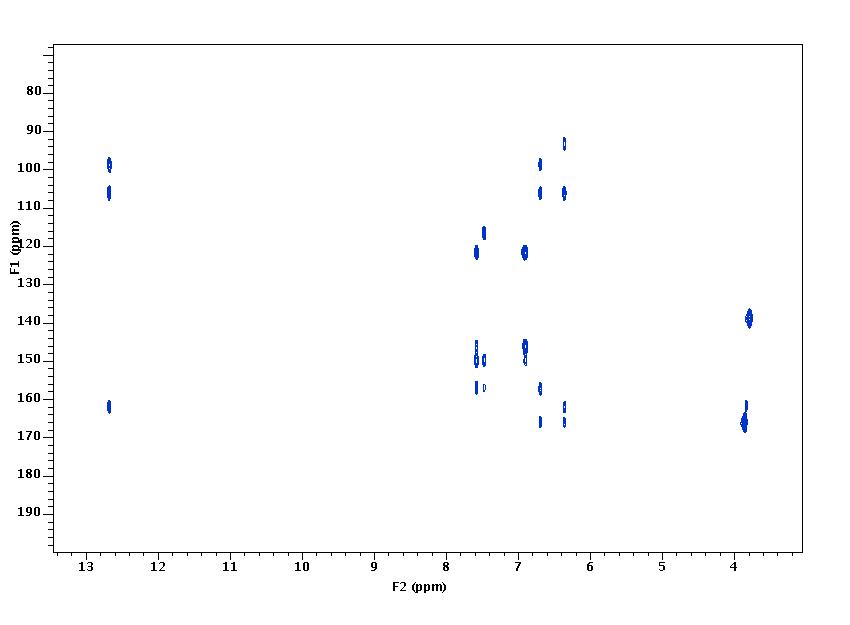


**HMBC-NMR**

**Figure S 15.** NMR spectra of 3,7-di-*O*-methylquercetin


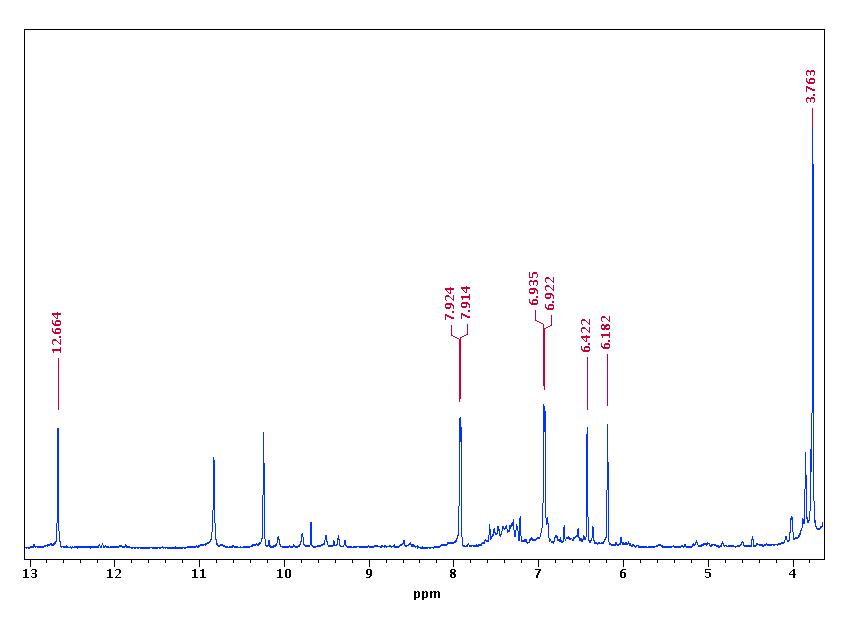


**^1^H-NMR**


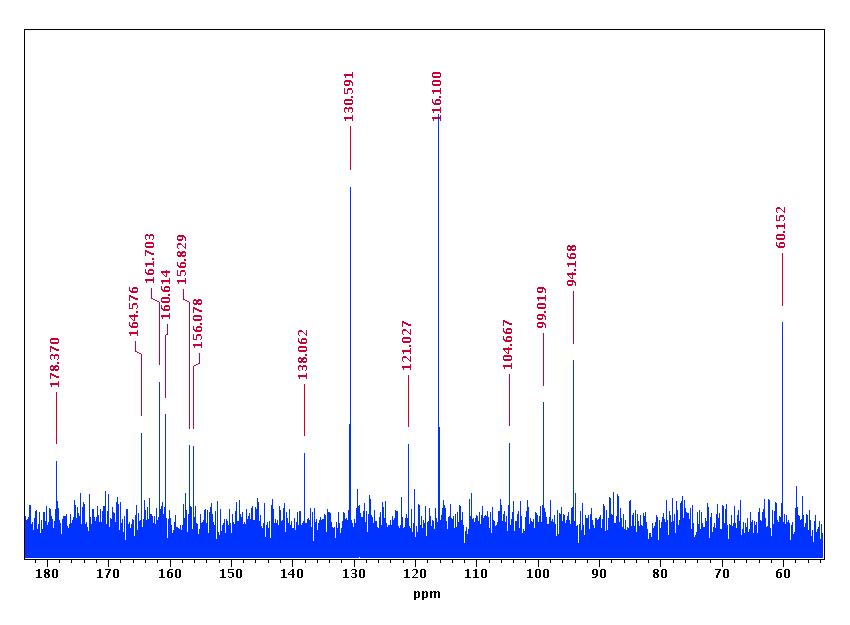


**^13^C-NMR**


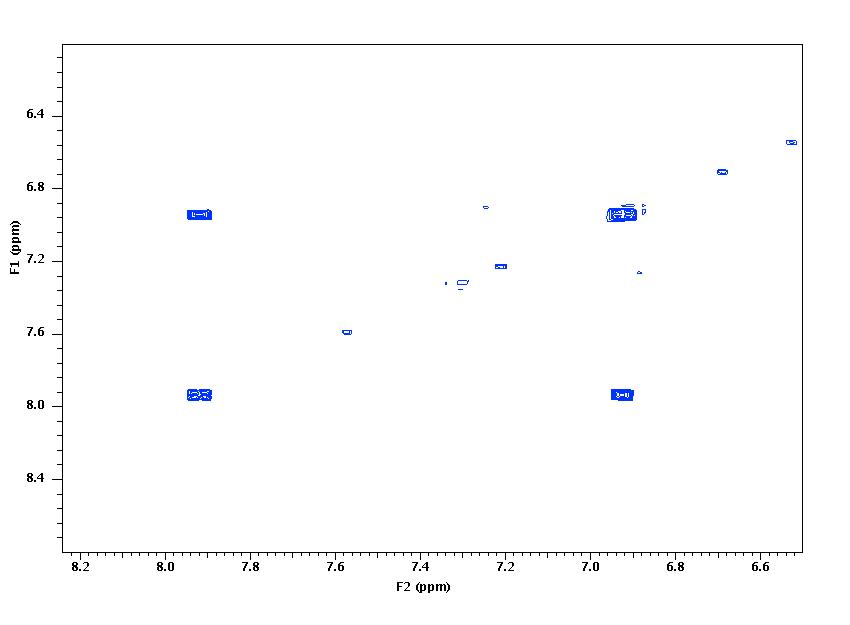


**COSY-NMR**


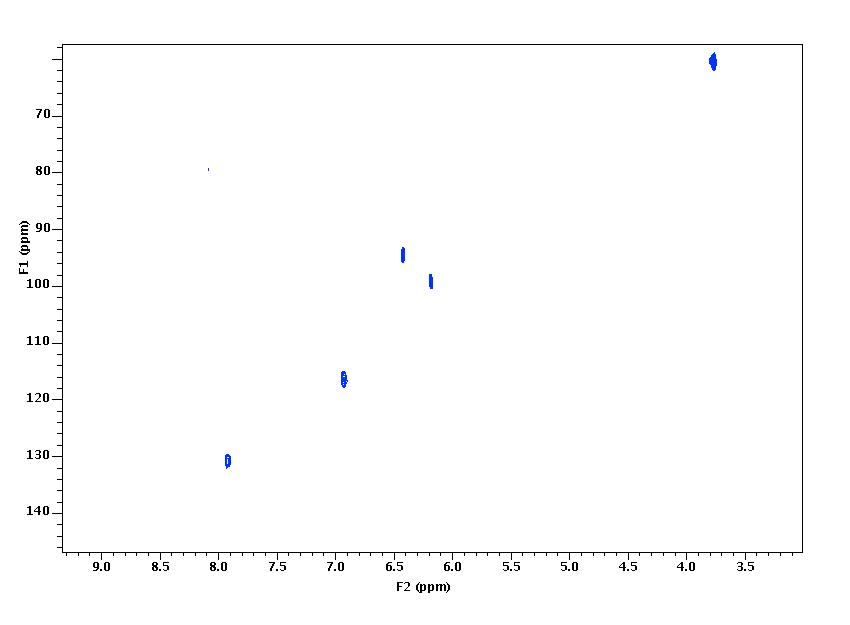


**HSQC-NMR**


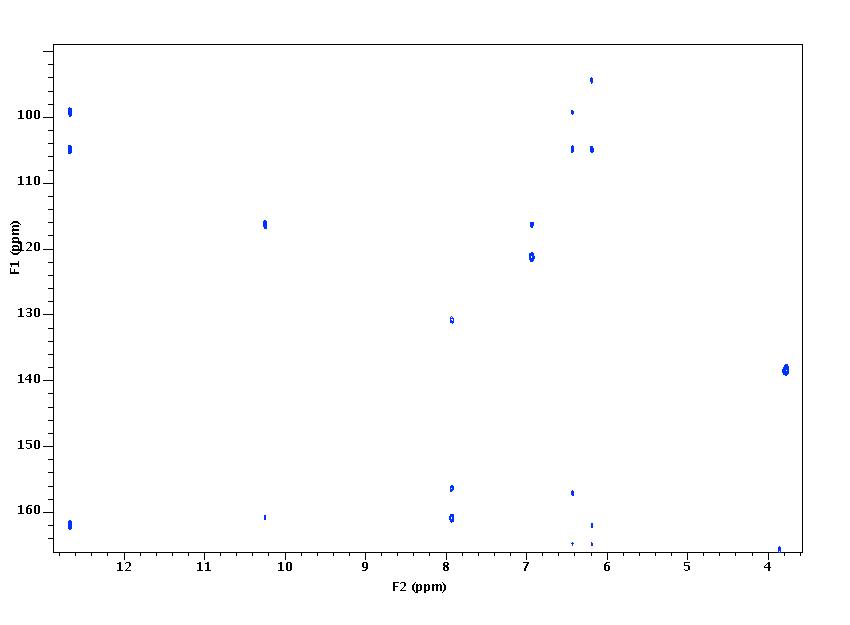


**HMBC-NMR**

**Figure S 16.** NMR spectra of 3-methoxy kaempferol


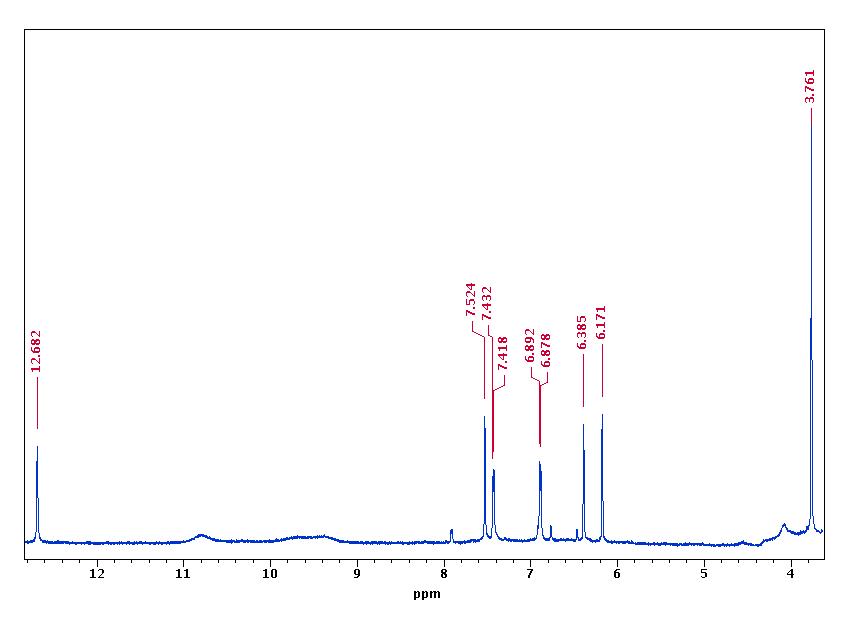


**^1^H-NMR**


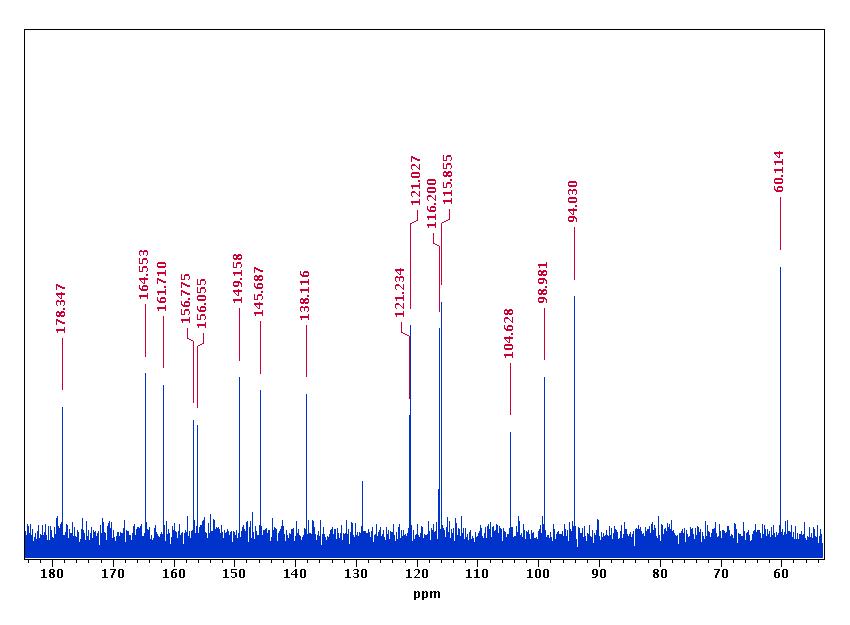


**^13^C-NMR**


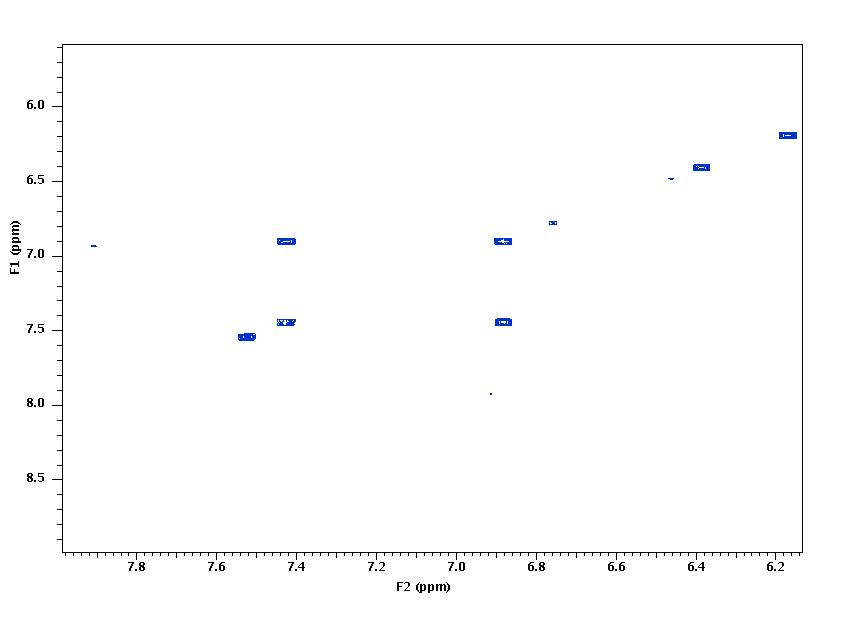


**COSY-NMR**


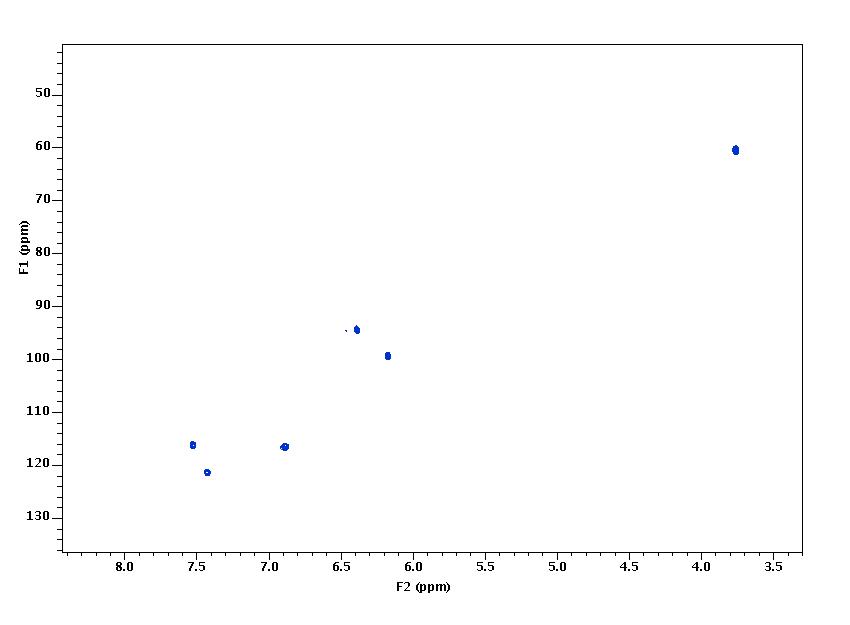


**HSQC-NMR**


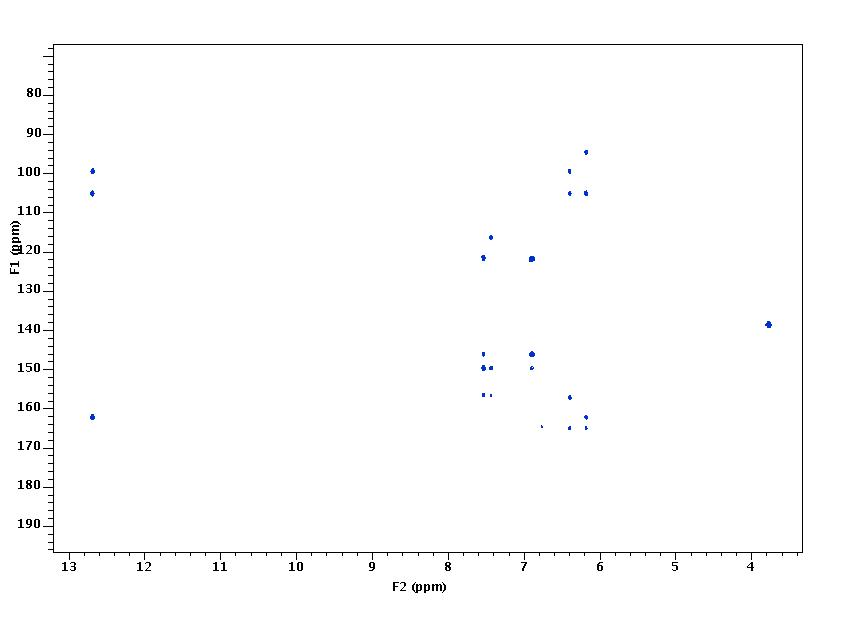


**HMBC-NMR**

**Figure S 17.** NMR spectra of 3-*O*-methylquercetin

**
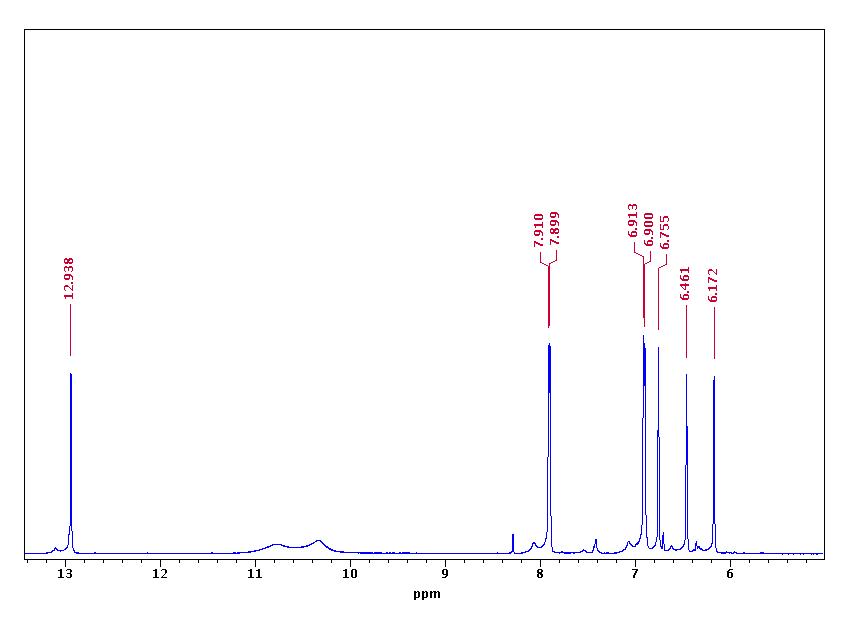
**

**^1^H-NMR 600 MHz**

**
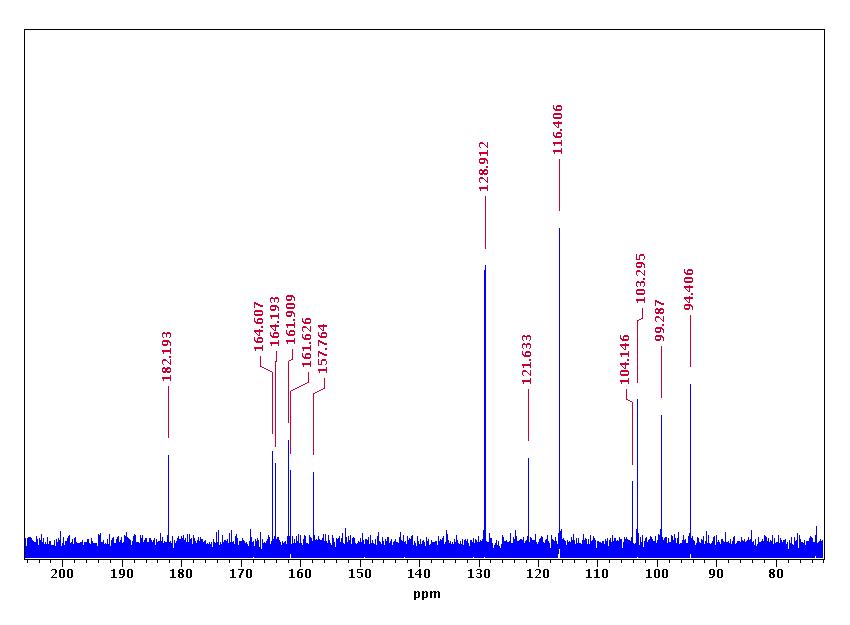
**

**^13^C-NMR 150 MHz**

**
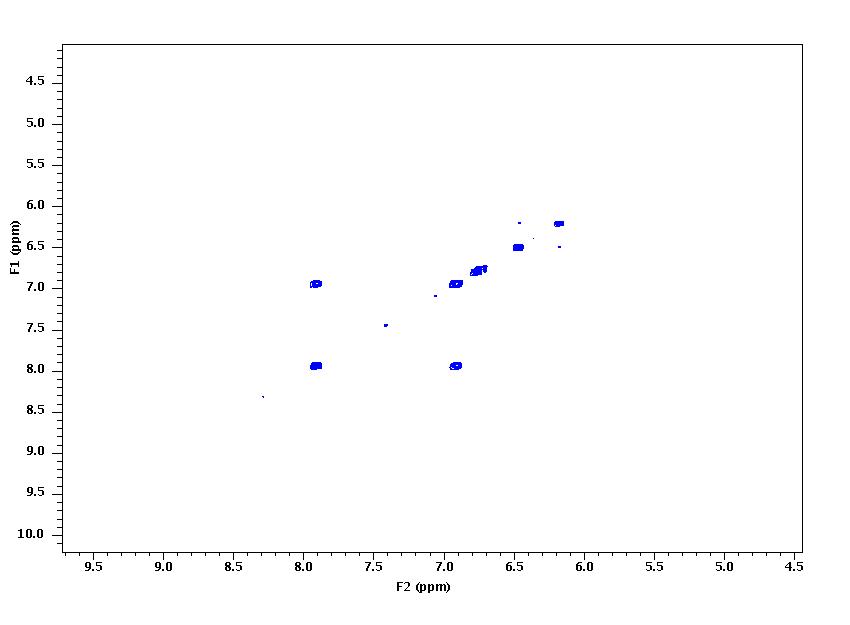
**

**COSY-NMR**

**
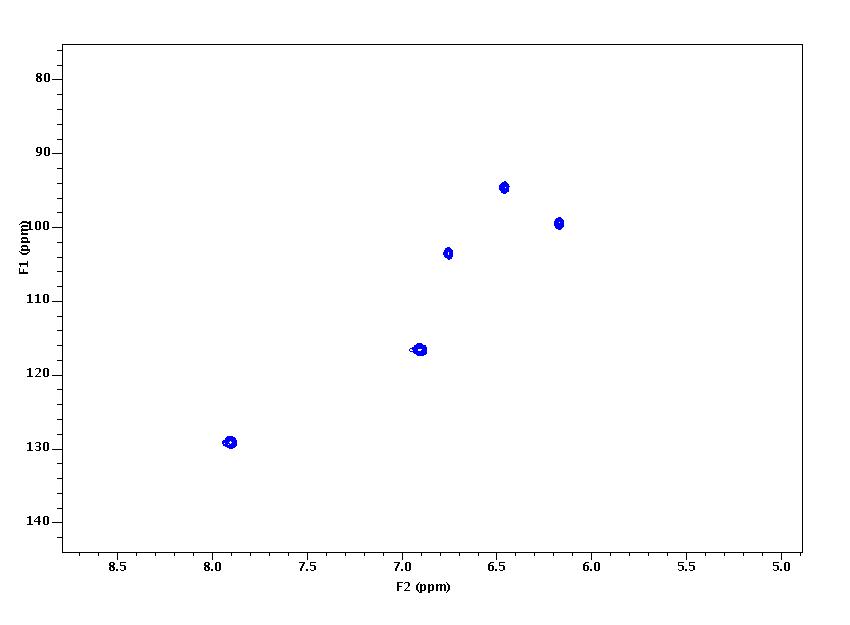
**

**HSQC -NMR**

**
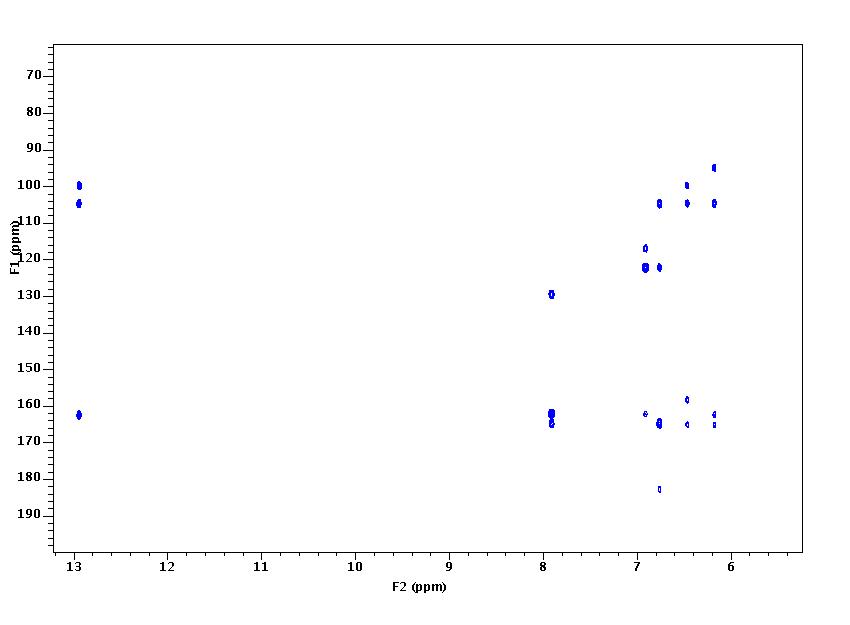
**

**HMBC-NMR**

**Figure S 18.** NMR spectra of apigenin

**
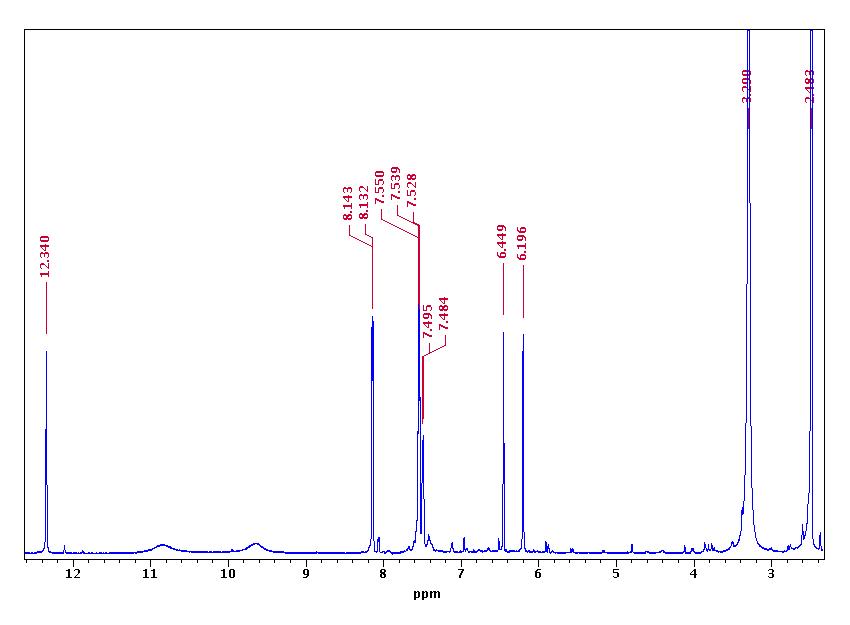
**

**^1^H-NMR 600 MHz**

**
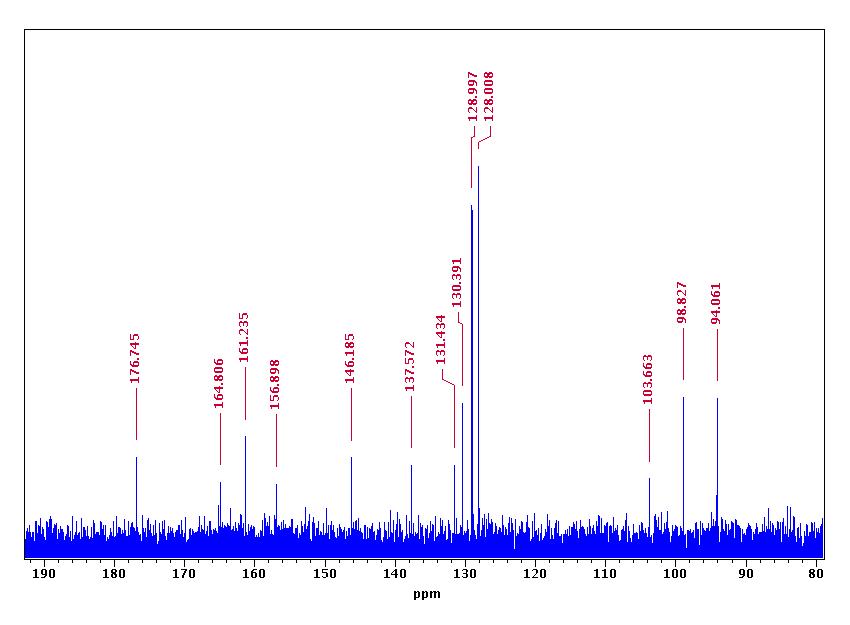
**

**^13^C-NMR 150 MHz**

**
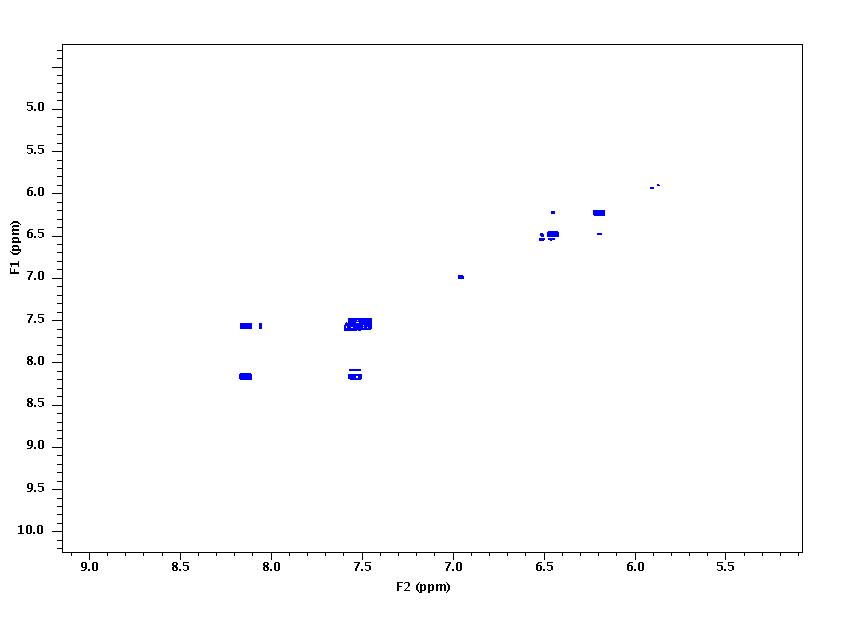
**

**COSY-NMR**

**
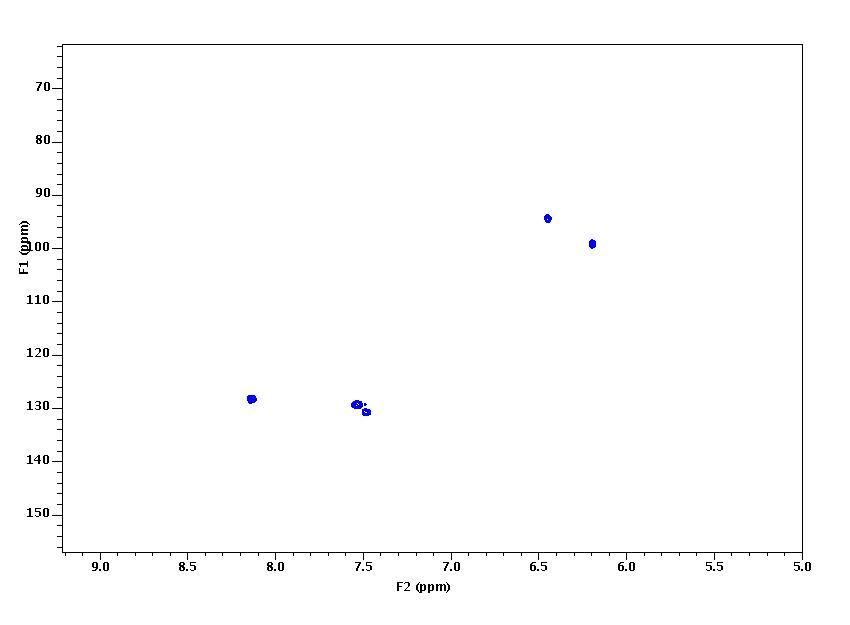
**

**HSQC-NMR**

**
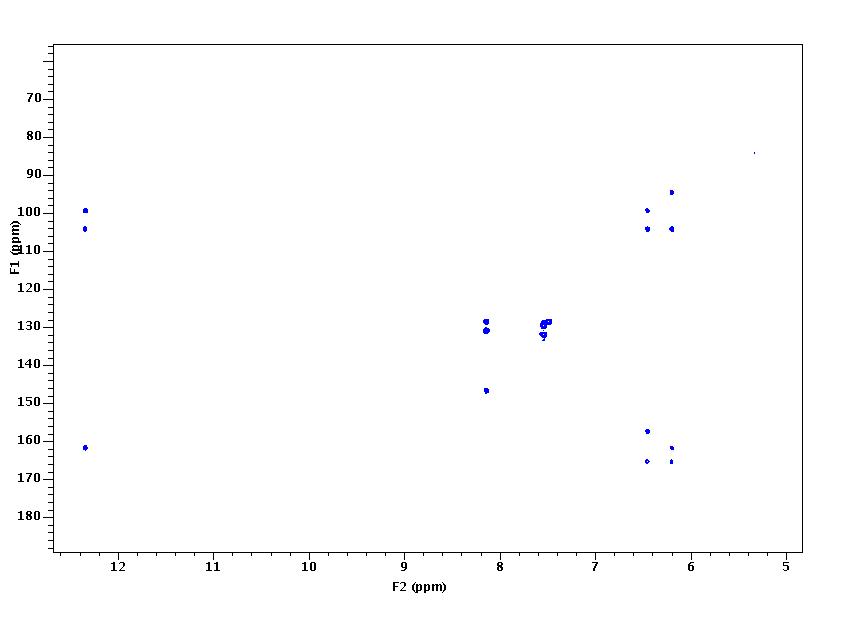
**

**HMBC-NMR**

**Figure S 19.** NMR spectra of galangin

UDC 547.913:539.26


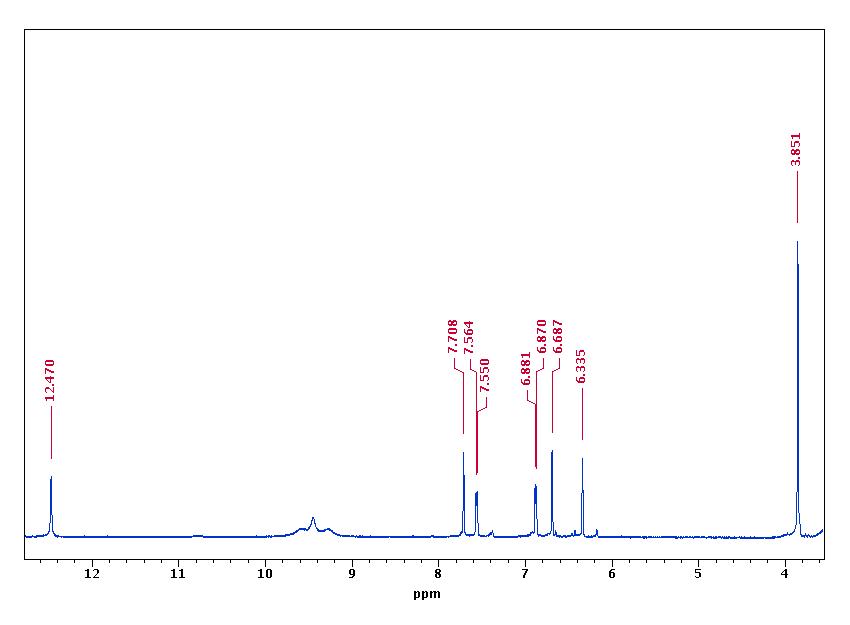


**^1^H-NMR**


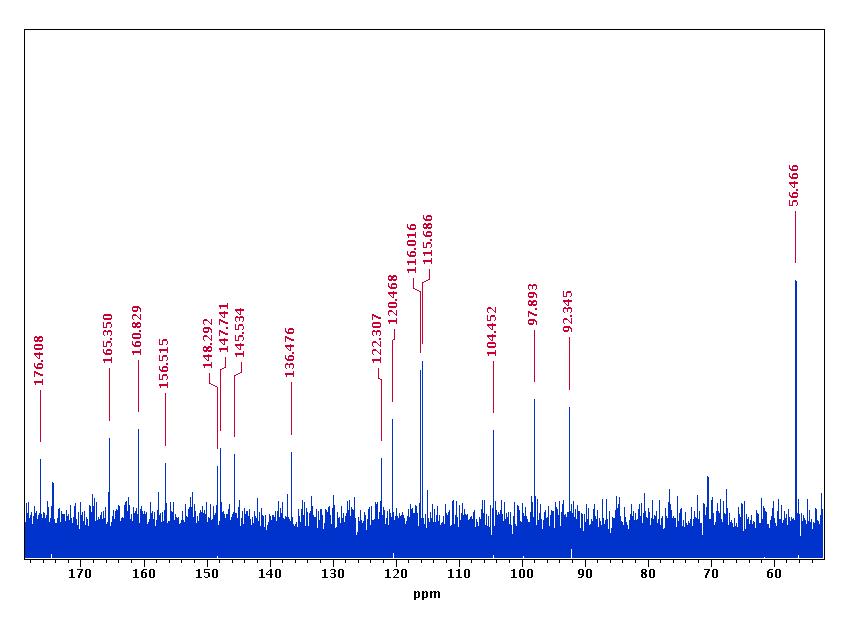


**^13^C-NMR**


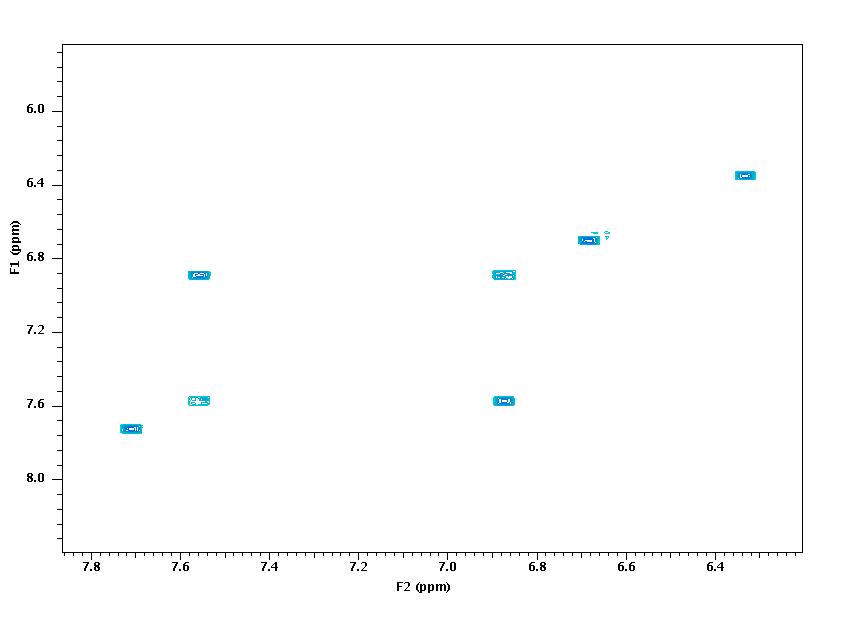


**COSY-NMR**


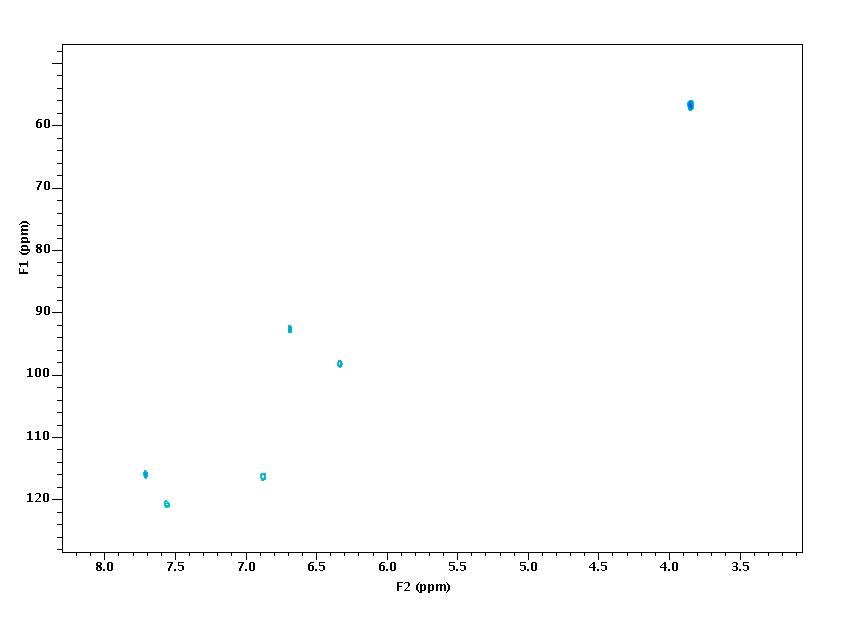


**HSQC-NMR**


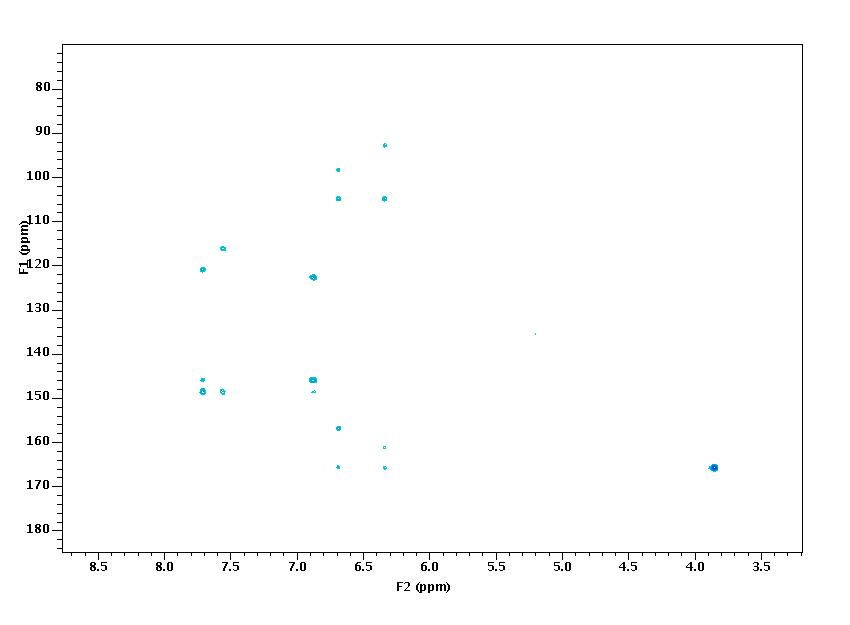


**HMBC-NMR**

**Figure S 20**. NMR spectra of rhamnetin


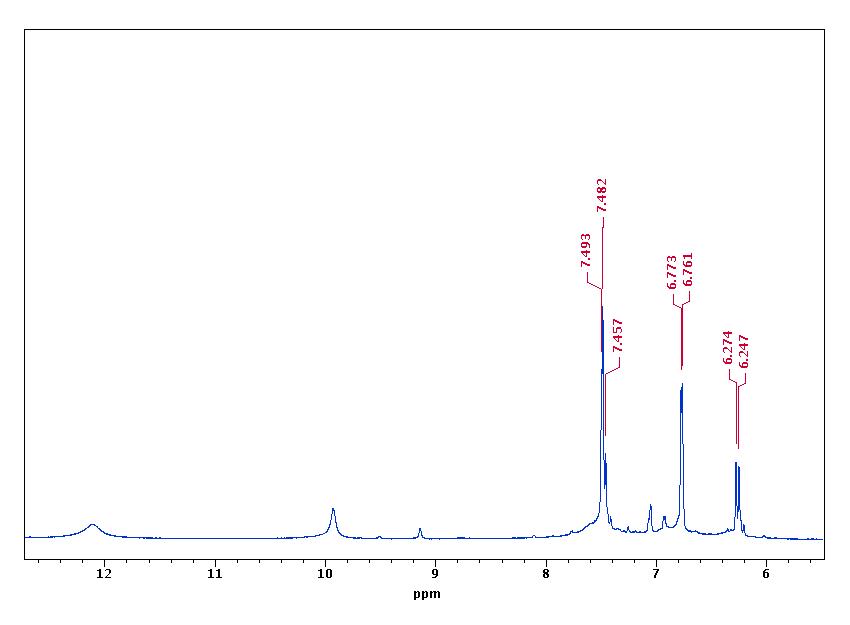


**^1^H-NMR**


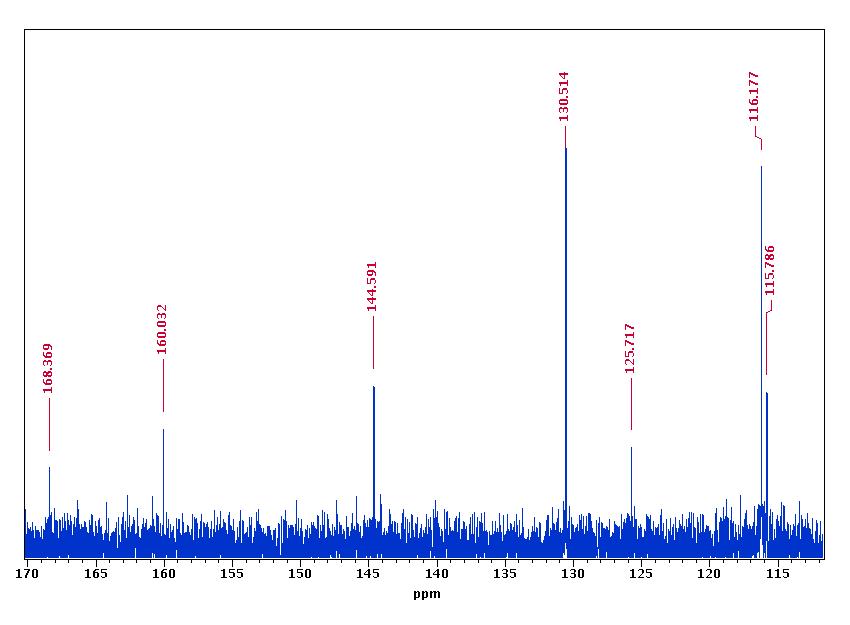


**^13^C-NMR**


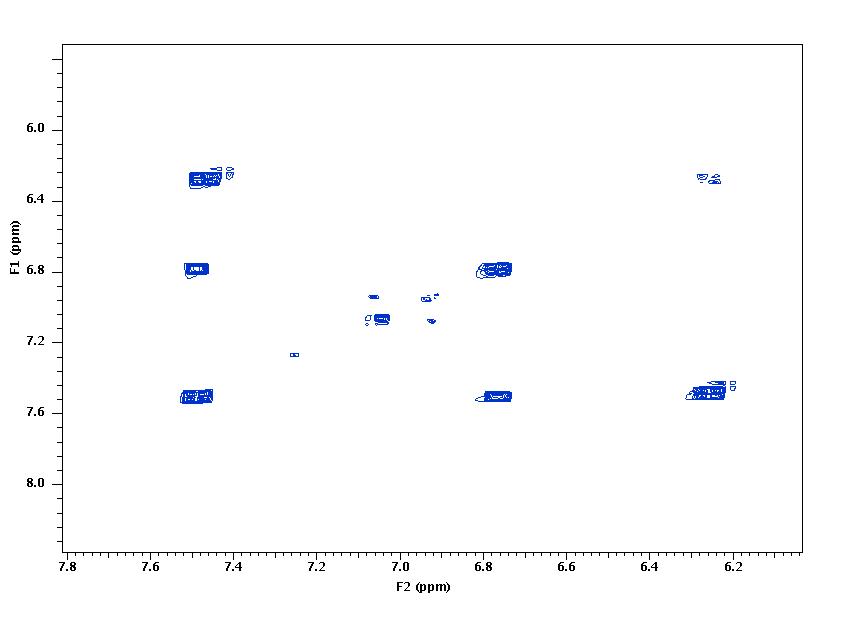


**COSY-NMR**


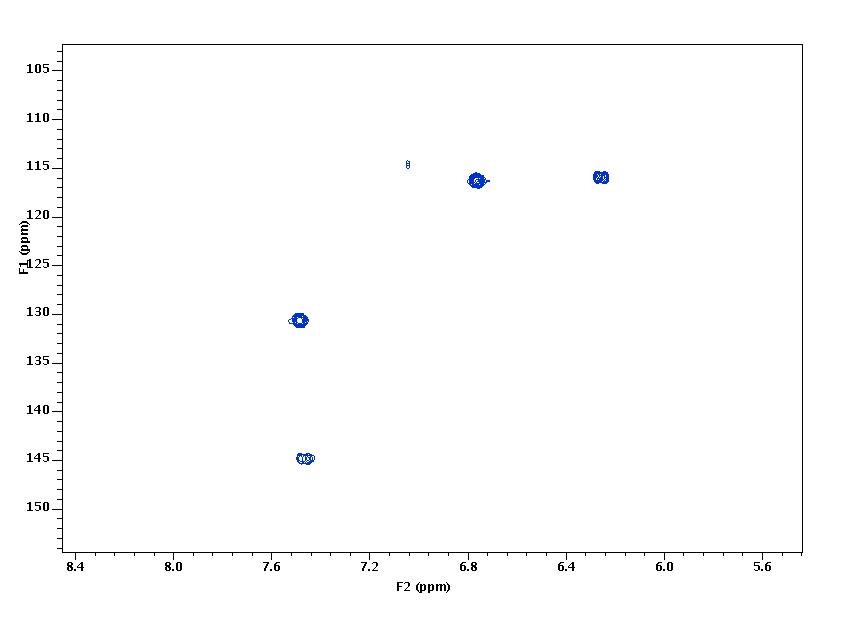


**HSQC-NMR**


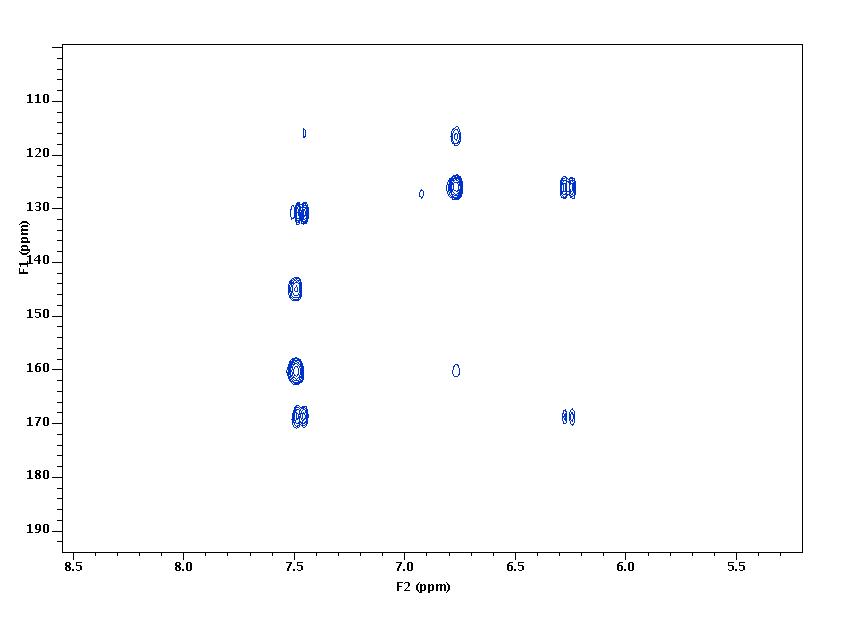


**HMBC-NMR**

**Figure S 21.** NMR spectra of *p*-coumaric acid.


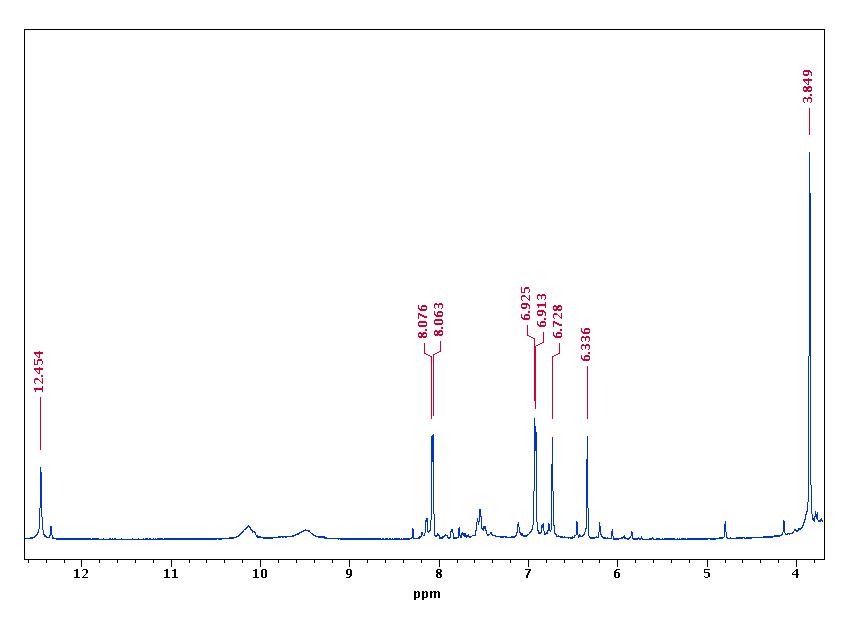


**^1^H-NMR**


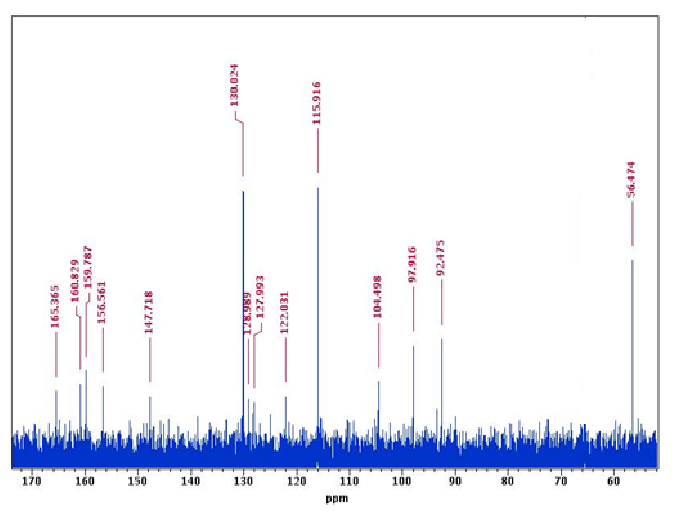


**^13^C-NMR**


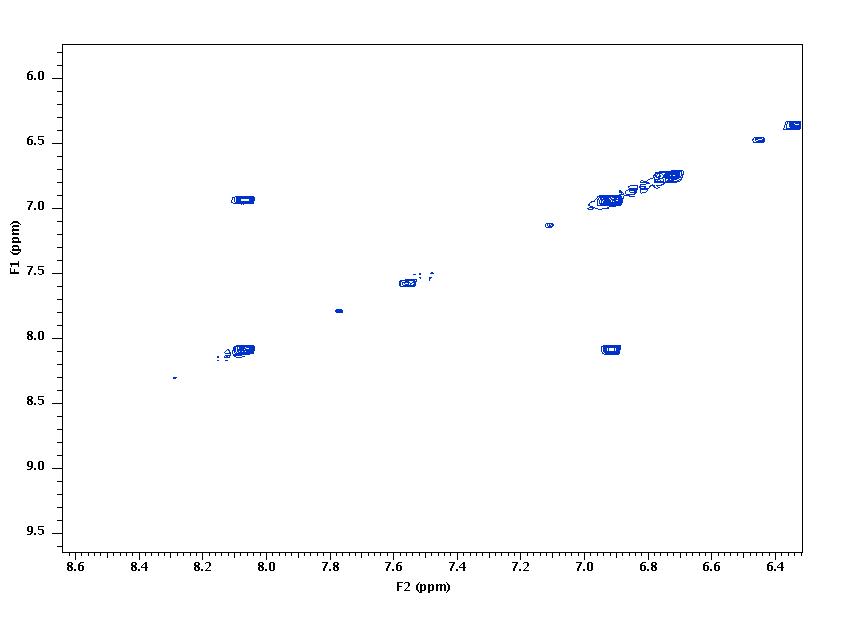


**COSY-NMR**


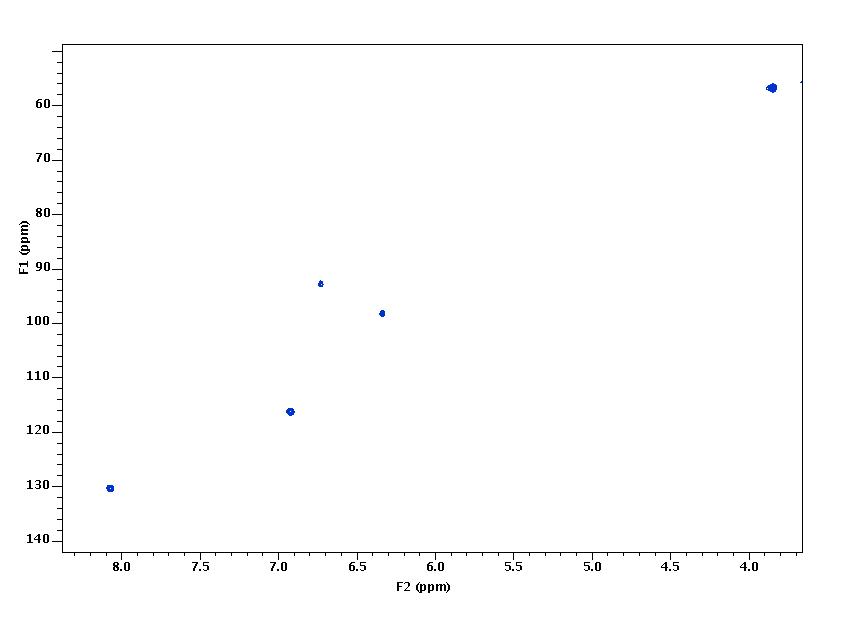


**HSQC-NMR**


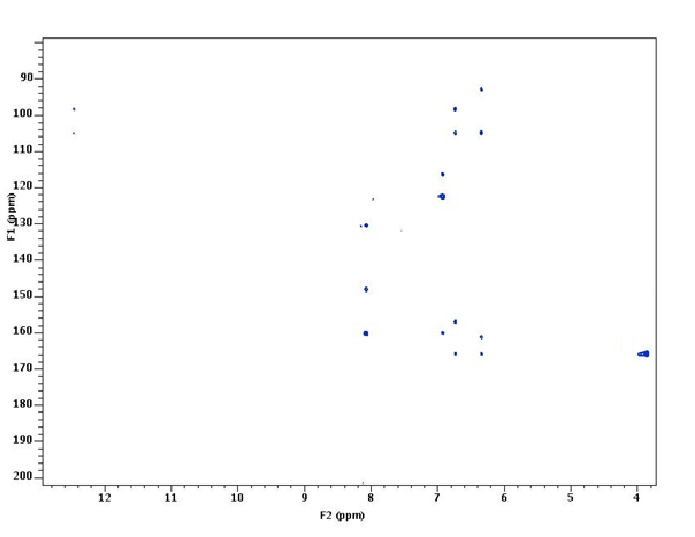


**HMBC-NMR**

**Figure S 22.** NMR spectra of rhamnocitrin

**TABLES**

**Table S 1**

|  | Apigenin [1] | | Galangin [1] | | Pinobanksin [2] | | Chrysin [1] | | Genkwanin [3] | | Rhamnetin [9] | | Tectochrysin [8] | | Rhamnocitrin [5] | | 3,4-Dimethoxy cinnamic acid [11] | |
| --- | --- | --- | --- | --- | --- | --- | --- | --- | --- | --- | --- | --- | --- | --- | --- | --- | --- | --- |
| 2 |  |  |  |  | 5,16 | 5,18 |  |  |  |  |  |  |  |  |  |  | 7,28 | 7,07 |
| 3 | 6,75 | 6,75 |  |  | 4,60 | 4,16 | 6,69 | 6,94 | 6,82 | 6,77 |  |  | 6,61 | 6,68 |  |  |  |  |
| 5 |  |  |  |  |  |  |  |  |  |  |  |  |  |  |  |  | 6,95 | 6,88 |
| 6 | 6,17 | 6,19 | 6,19 | 6,16 | 5,88 | 5,88 | 6,20 | 6,22 | 6,35 | 6,31 | 6,33 | 6,34 | 6,38 | 6,45 | 6,33 | 6,25 | 7,18 | 7,14 |
| 8 | 6,47 | 6,46 | 6,44 | 6,40 | 5,91 | 5,92 | 6,52 | 6,51 | 6,75 | 6,69 | 6,68 | 6,69 | 6,50 | 6,60 | 6,72 | 6,60 | 7,50 | 7,73 |
| 2’ | 7,90 | 7,90 | 8,13 | 8,08 | 7,50 | 7,52 | 8,06 | 8,04 | 7,94 | 7,89 | 7,70 | 7,72 | 7,88 | 7,97 | 8,02 | 8,05 | 6,41 | 6,32 |
| 3’ | 6,90 | 6,92 | 7,54 | 7,44 | 7,40 | 7,40 | 7,57 | 7,58 | 6,91 | 6,88 |  |  | 7,52 | 7,58 | 6,91 | 6,92 |  |  |
| 4’ |  |  | 7,48 | 7,44 | 7,40 | 7,40 | 7,59 | 7,58 |  |  |  |  | 7,54 | 7,58 |  |  |  |  |
| 5’ | 6,90 | 6,92 | 7,54 | 7,44 | 7,40 | 7,40 | 7,57 | 7,58 | 6,91 | 6,88 | 6,88 | 6,89 | 7,52 | 7,58 | 6,91 | 6,92 |  |  |
| 6’ | 7,90 | 7,90 | 8,13 | 8,08 | 7,50 | 7,52 | 8,06 | 8,04 | 7,94 | 7,89 | 7,56 | 7,57 | 7,88 | 7,97 | 8,02 | 8,05 |  |  |
| 3-OMe |  |  |  |  |  |  |  |  |  |  |  |  |  |  |  |  | 3,78 | 3,92 |
| 4-OMe |  |  |  |  |  |  |  |  |  |  |  |  |  |  |  |  | 3,76 | 3,92 |
| 7’’ |  |  |  |  |  |  |  |  | 3,85 | 3,81 | 3,85 | 3,86 | 3,88 | 3,90 | 3,84 | 3,80 |  |  |

**Table S 2**

|  | Pinocembrin [1] | | 3,7-dietoxy quercetin [4] | | 3-methoxy quercetin[6] | | İso-kaempferide[7] | | Pinostrobin [1] | | ferulic acid [1] | | caffeic acid [1] | | p-coumaric acid [1] | | Fraxetin [10] | |
| --- | --- | --- | --- | --- | --- | --- | --- | --- | --- | --- | --- | --- | --- | --- | --- | --- | --- | --- |
| 2 | 5,55 | 5,58 |  |  |  |  |  |  | 5,43 | 5,62 | 7,05 | 7,28 | 7,00 | 7,03 | 7,48 | 7,51 |  |  |
| 3 | 2,76-3,22 | 2,79-3,23 |  |  |  |  |  |  | 3,08-2,83 | 3,29-2,83 |  |  |  |  | 6,72 | 6,81 | 6,19 | 6,23 |
| 4 |  |  |  |  |  |  |  |  |  |  |  |  |  |  |  |  | 7,86 | 7,84 |
| 5 |  |  |  |  |  |  |  |  |  |  | 6,69 | 6,79 | 6,73 | 6,76 | 6,72 | 6,81 | 6,78 | 6,72 |
| 6 | 5,88 | 5,91 | 6,35 | 6,29 | 6,17 | 6,22 | 6,42 | 6,40 | 6,06 | 6,11 | 7,06 | 7,08 | 6,93 | 6,96 | 7,48 | 7,51 |  |  |
| 7 |  |  |  |  |  |  |  |  |  |  | 7,42 | 7,49 | 7,39 | 7,42 | 7,46 | 7,51 |  |  |
| 8 | 5,91 | 5,95 | 6,68 | 6,62 | 6,38 | 6,43 | 6,18 | 6,20 | 6,07 | 6,15 | 6,21 | 6,36 | 6,14 | 6,17 | 6,26 | 6,29 |  |  |
| 2’ | 7,49 | 7,52 | 7,57 | 7,57 | 7,52 | 7,77 | 7,92 | 7,98 | 7,45 | 7,54 |  |  |  |  |  |  |  |  |
| 3’ | 7,41 | 7,41 |  |  |  |  | 6,93 | 6,93 | 7,43 | 7,42 |  |  |  |  |  |  |  |  |
| 4’ | 7,37 | 7,41 |  |  |  |  |  |  | 7,38 | 7,42 |  |  |  |  |  |  |  |  |
| 5’ | 7,41 | 7,41 | 6,89 | 6,90 | 6,88 | 6,92 | 6,93 | 6,93 | 7,43 | 7,42 |  |  |  |  |  |  |  |  |
| 6’ | 7,49 | 7,52 | 7,46 | 7,45 | 7,42 | 7,67 | 7,92 | 7,98 | 7,45 | 7,54 |  |  |  |  |  |  |  |  |
| 3-OMe |  |  | 3,78 | 3,77 | 3,76 |  | 3,76 | 3,78 |  |  | 3,78 | 3,82 |  |  |  |  |  |  |
| 6-OMe |  |  |  |  |  |  |  |  |  |  |  |  |  |  |  |  | 3,79 | 3,87 |
| 7-OMe |  |  | 3,84 | 3,81 |  |  |  |  | 3,81 | 3,80 |  |  |  |  |  |  |  |  |

**Table S 3**

|  | Apigenin [1] | | Galangin [1] | | Pinobanksin [2] | | Chrysin [1] | | Genkwanin [3] | | Pinocembrin [1] | | 3,7-dimetoxy quercetin [4] | | İsokaempferide  [7] | | Rhamnocitrin  [5] | |
| --- | --- | --- | --- | --- | --- | --- | --- | --- | --- | --- | --- | --- | --- | --- | --- | --- | --- | --- |
| 1 |  |  |  |  |  |  |  |  |  |  |  |  |  |  |  |  |  |  |
| 2 | 164,19 | 164,59 | 146,18 | 146,11 | 83,33 | 82,80 | 163,80 | 163,60 | 164,53 | 164,01 | 78,82 | 78,21 | 149,29 | 155,9 | 156,07 | 156,20 | 147,71 | 147,20 |
| 3 | 103,29 | 103,31 | 137,57 | 137,52 | 71,96 | 71,50 | 105,82 | 105,63 | 103,47 | 102,96 | 42,55 | 42,77 | 138,33 | 137,8 | 138,06 | 136,90 | 138,00 | 138,00 |
| 4 | 182,19 | 182,19 | 176,74 | 176,68 | 197,98 | 197,40 | 182,39 | 182,30 | 182,38 | 181,82 | 196,41 | 196,45 | 178,50 | 178,8 | 178,37 | 176,80 | 169,00 | 176,00 |
| 5 | 161,90 | 161,62 | 161,28 | 161,19 | 162,88 | 163,30 | 161,87 | 161,94 | 161,65 | 157,16 | 163,94 | 164,10 | 161,40 | 160,9 | 161,70 | 160,60 | 160,82 | 160,40 |
| 6 | 99,28 | 99,30 | 98,82 | 98,74 | 95,53 | 96,10 | 99,17 | 99,49 | 98,40 | 97,86 | 96,38 | 96,50 | 98,19 | 97,6 | 99,01 | 92,30 | 97,91 | 97,40 |
| 7 | 164,60 | 164,19 | 164,80 | 164,65 | 163,77 | 167,00 | 165,03 | 164,91 | 165,58 | 165,05 | 167,15 | 167,23 | 165,56 | 165,00 | 164,57 | 163,60 | 165,35 | 164,70 |
| 8 | 94,40 | 94,42 | 94,06 | 93,99 | 96,61 | 95,10 | 94,73 | 94,58 | 93,14 | 92,60 | 95,50 | 95,62 | 92,68 | 92,10 | 94,16 | 97,30 | 92,47 | 92,00 |
| 9 | 157,76 | 157,77 | 156,89 | 156,83 | 163,77 | 162,40 | 157,81 | 157,91 | 157,68 | 161,18 | 163,17 | 163,65 | 156,72 | 156,10 | 156,82 | 156,00 | 156,56 | 156,20 |
| 10 | 104,14 | 104,18 | 103,66 | 103,65 | 100,90 | 100,30 | 104,48 | 104,44 | 105,11 | 104,61 | 102,26 | 102,30 | 105,65 | 105,10 | 104,66 | 103,40 | 104,44 | 104,00 |
| 1’ | 121,63 | 121,66 | 131,43 | 131,38 | 137,74 | 137,20 | 131,13 | 131,13 | 121,51 | 121,01 | 139,14 | 139,64 | 121,50 | 120,70 | 121,02 | 120,10 | 122,03 | 121,60 |
| 2’ | 128,91 | 128,90 | 128,00 | 127,94 | 128,50 | 128,00 | 126,81 | 126,84 | 129,02 | 128,45 | 127,05 | 127,12 | 116,00 | 115,50 | 130,59 | 128,90 | 130,02 | 129,60 |
| 3’ | 116,40 | 116,41 | 128,99 | 128,88 | 128,64 | 128,10 | 129,71 | 129,56 | 116,42 | 115,89 | 128,99 | 129,13 | 145,72 | 145,20 | 116,10 | 114,10 | 115,90 | 115,50 |
| 4’ | 161,62 | 161,93 | 130,39 | 130,28 | 129,03 | 128,60 | 132,61 | 132,42 | 161,76 | 161,18 | 128,99 | 129,13 | 156,43 | 148,80 | 160,61 | 159,30 | 159,78 | 159,30 |
| 5’ | 116,40 | 116,41 | 128,99 | 128,88 | 128,64 | 128,10 | 129,71 | 129,56 | 116,42 | 115,89 | 128,99 | 129,13 | 116,17 | 115,70 | 116,10 | 114,10 | 115,90 | 115,50 |
| 6’ | 128,91 | 128,90 | 128,00 | 127,94 | 128,50 | 128,00 | 126,81 | 126,84 | 129,02 | 128,45 | 127,05 | 127,12 | 121,10 | 120,60 | 130,59 | 128,90 | 130,02 | 129,60 |
| 3-OMe |  |  |  |  |  |  |  |  |  |  |  |  | 60,12 | 59,50 | 60,15 | 59,50 |  |  |
| 7-OMe |  |  |  |  |  |  |  |  | 56,49 | 55,95 |  |  | 56,52 | 56,00 |  |  | 56,47 | 56,00 |

**Table S 4**

|  | 3-methoxy  Quercetin [6] | | Fraxetin [10] | | Pinostrobin [1] | | Tectochrysin [8] | | ferulic acid [1] | | caffeic acid [1] | | p-coumaric  acid [1] | | 3,4-Dimethoxy  cinnamic acid [11] | | Rhamnetin [9] | |
| --- | --- | --- | --- | --- | --- | --- | --- | --- | --- | --- | --- | --- | --- | --- | --- | --- | --- | --- |
| 1 |  |  |  |  |  |  |  |  | 127,52 | 126,24 | 126,13 | 126,16 | 125,71 | 130,53 | 127,51 | 127,20 |  |  |
| 2 | 156,05 | 147,60 | 160,95 | 164,00 | 79,22 | 79,03 | 163,99 | 162,72 | 114,52 | 111,95 | 115,05 | 115,09 | 130,51 | 125,74 | 110,79 | 110,22 | 147,74 | 147,40 |
| 3 | 138,11 | 135,60 | 112,27 | 112,60 | 43,39 | 42,63 | 105,90 | 108,73 | 147,12 | 148,38 | 145,99 | 146,02 | 116,17 | 116,20 | 149,43 | 149,40 | 136,47 | 136,10 |
| 4 | 178,34 | 175,70 | 145,79 | 146,70 | 195,74 | 196,92 | 182,50 | 180,23 | 150,30 | 149,55 | 148,56 | 148,59 | 160,03 | 160,04 | 151,23 | 151,20 | 176,40 | 176,00 |
| 5 | 161,71 | 160,70 | 100,79 | 101,30 | 164,16 | 163,71 | 162,21 | 163,32 | 112,44 | 115,99 | 116,18 | 116,21 | 116,17 | 116,20 | 112,01 | 109,99 | 160,82 | 160,40 |
| 6 | 99,35 | 98,20 | 145,48 | 140,80 | 94,26 | 95,24 | 98,18 | 97,87 | 121,44 | 123,27 | 121,58 | 121,60 | 130,51 | 125,74 | 123,07 | 123,20 | 97,97 | 97,50 |
| 7 | 164,55 | 163,90 | 139,82 | 147,00 | 168,02 | 167,94 | 165,71 | 165,57 | 144,62 | 144,95 | 145,01 | 145,04 | 144,59 | 144,60 | 144,63 | 147,10 | 165,35 | 165,00 |
| 8 | 94,03 | 93,40 | 133,30 | 140,70 | 95,14 | 94,34 | 92,69 | 96,66 | 116,69 | 116,09 | 115,55 | 115,58 | 115,76 | 115,81 | 117,18 | 115,00 | 92,23 | 92,00 |
| 9 | 156,77 | 156,20 | 139,72 | 134,00 | 162,78 | 163,09 | 157,81 | 161,58 | 168,20 | 168,42 | 168,02 | 168,34 | 168,36 | 168,38 | 168,30 | 172,50 | 156,51 | 156,10 |
| 10 | 104,62 | 103,00 | 110,68 | 112,20 | 103,15 | 103,10 | 105,90 | 108,50 |  |  |  |  |  |  |  |  | 104,45 | 104,10 |
| 1’ | 121,23 | 122,00 |  |  | 138,37 | 138,99 | 131,35 | 132,68 |  |  |  |  |  |  |  |  | 122,30 | 121,90 |
| 2’ | 115,76 | 115,30 |  |  | 126,05 | 127,06 | 126,29 | 130,37 |  |  |  |  |  |  |  |  | 115,63 | 115,30 |
| 3’ | 149,15 | 145,00 |  |  | 128,86 | 129,06 | 129,07 | 127,35 |  |  |  |  |  |  |  |  | 145,53 | 145,10 |
| 4’ | 145,68 | 146,90 |  |  | 128,86 | 129,06 | 131,35 | 132,88 |  |  |  |  |  |  |  |  | 148,29 | 147,90 |
| 5’ | 116,20 | 115,60 |  |  | 128,86 | 129,06 | 129,07 | 127,35 |  |  |  |  |  |  |  |  | 116,01 | 115,60 |
| 6’ | 121,02 | 120,00 |  |  | 126,05 | 127,06 | 126,29 | 130,37 |  |  |  |  |  |  |  |  | 120,46 | 120,10 |
| 3-OMe | 60,11 | 60,15 |  |  |  |  |  |  | 56,07 | 56,17 |  |  |  |  | 56,07 | 56,10 |  |  |
| 4-OMe |  |  |  |  |  |  |  |  |  |  |  |  |  |  | 56,01 | 56,00 |  |  |
| 6-OMe |  |  | 56,50 | 56,80 |  |  |  |  |  |  |  |  |  |  |  |  |  |  |
| 7-OMe |  |  |  |  | 55,67 | 56,33 | 55,79 | 56,64 |  |  |  |  |  |  |  |  | 56,46 | 56,10 |

**REFERENCES**

**[1]** Bertelli, D., Papotti, G., Bortolotti, L., Marcazzan, G. L., & Plessi, M. (2012). 1H‐NMR Simultaneous Identification of Health‐Relevant Compounds in Propolis Extracts. *Phytochemical Analysis*, *23*(3), 260-266.

### [2] Komoda, Y. (1989). Isolation of flavonoids from Populus nigra as Δ4-3-ketosteroid (5α) reductase inhibitors. *Chemical and pharmaceutical bulletin*, *37*(11), 3128-3130.

### [3] Isaev, I. M., Agzamova, M. A., & Isaev, M. I. (2011). Genkwanin and iridoid glycosides from Leonurus turkestanicus. *Chemistry of Natural Compounds*, *47*(1), 132.

**[4]** Guerrero, M. F., Puebla, P., Carrón, R., Martin, M. L., & Román, L. S. (2002). Quercetin 3, 7‐dimethyl ether: a vasorelaxant flavonoid isolated from Croton schiedeanus Schlecht. *Journal of pharmacy and pharmacology*, *54*(10), 1373-1378.

**[5]** Tu, Y. C., Lian, T. W., Yen, J. H., Chen, Z. T., & Wu, M. J. (2007). Antiatherogenic effects of kaempferol and rhamnocitrin. *Journal of agricultural and food chemistry*, *55*(24), 9969-9976.

**[7]** Mai, L. H., Chabot, G. G., Grellier, P., Quentin, L., Dumontet, V., Poulain, C., ... & Grougnet, R. (2015). Antivascular and anti-parasite activities of natural and hemisynthetic flavonoids from New Caledonian Gardenia species (Rubiaceae). *European journal of medicinal chemistry*, *93*, 93-100.

**[8]** Hasan, M. M., Ahmed, Q. U., Soad, S. Z. M., Latip, J., Taher, M., Syafiq, T. M. F., ... & Zakaria, Z. A. (2017). Flavonoids from Tetracera indica Merr. induce adipogenesis and exert glucose uptake activities in 3T3-L1 adipocyte cells. *BMC complementary and alternative medicine*, *17*(1), 431.

**[9]** Lee, E. J., Moon, B. H., Park, Y., Hong, S. W., Lee, S. H., Lee, Y. G., & Lim, Y. H. (2008). Effects of hydroxy and methoxy substituents on NMR data in flavonols. *Bulletin of the Korean Chemical Society*, *29*(2), 507-510.

### [10] Liu, R., Sun, Q., Sun, A., & Cui, J. (2005). Isolation and purification of coumarin compounds from Cortex fraxinus by high-speed counter-current chromatography. *Journal of chromatography A*, *1072*(2), 195-199.

**[11]** Guzmán-Gutiérrez, S. L., Nieto-Camacho, A., Castillo-Arellano, J. I., Huerta-Salazar, E., Hernández-Pasteur, G., Silva-Miranda, M., ... & Reyes-Chilpa, R. (2018). Mexican propolis: A source of antioxidants and anti-inflammatory compounds, and isolation of a novel chalcone and ε-caprolactone derivative. *Molecules*, *23*(2), 334.
